# Prognostic accuracy and clinical utility of psychometric instruments for individuals at clinical high-risk of psychosis: a systematic review and meta-analysis

**DOI:** 10.1038/s41380-022-01611-w

**Published:** 2022-06-03

**Authors:** Dominic Oliver, Maite Arribas, Joaquim Radua, Gonzalo Salazar de Pablo, Andrea De Micheli, Giulia Spada, Martina Maria Mensi, Magdalena Kotlicka-Antczak, Renato Borgatti, Marco Solmi, Jae Il Shin, Scott W. Woods, Jean Addington, Philip McGuire, Paolo Fusar-Poli

**Affiliations:** 1 Early Psychosis: Interventions and Clinical-detection (EPIC) Lab, Department of Psychosis Studies, Institute of Psychiatry, Psychology & Neuroscience, King’s College London, London, UK; 2 Imaging of Mood- and Anxiety-Related Disorders (IMARD) Group, Institut d’Investigacions Biomèdiques August Pi i Sunyer (IDIBAPS), CIBERSAM, Barcelona, Spain; 3 Department of Clinical Neuroscience, Centre for Psychiatry Research, Karolinska Institute, Stockholm, Sweden; 4 Child and Adolescent Mental Health Services, South London & Maudsley NHS Trust, London, UK; 5 Department of Child and Adolescent Psychiatry, Institute of Psychiatry, Psychology & Neuroscience, King’s College London, London, UK; 6 OASIS Service, South London and Maudsley National Health Service (NHS) Foundation Trust, London, UK; 7 Department of Brain and Behavioral Sciences, University of Pavia, Pavia, Italy; 8 IRCCS Mondino Foundation, Childhood and Adolescent Neuropsychiatry Unit, Pavia, Italy; 9 Early Psychosis Diagnosis and Treatment Lab, Department of Affective and Psychotic Disorders, Medical University of Lodz, Lodz, Poland; 10 Department of Psychiatry, University of Ottawa, Ottawa, ON Canada; 11 Department of Mental Health, The Ottawa Hospital, Ottawa, ON Canada; 12 Clinical Epidemiology Program, Ottawa Hospital Research Institute (OHRI), University of Ottawa, Ottawa, ON Canada; 13 Department of Pediatrics, Yonsei University College of Medicine, Seoul, South Korea; 14 Department of Psychiatry, Yale University, New Haven, CT USA; 15 Department of Psychiatry, Hotchkiss Brain Institute, University of Calgary, Calgary, AB Canada; 16 Department of Psychosis Studies, Institute of Psychiatry, Psychology & Neuroscience, King’s College London, London, UK; 17 National Institute for Health Research, Maudsley Biomedical Research Centre, South London and Maudsley National Health Service (NHS) Foundation Trust, London, UK

**Keywords:** Prognostic markers, Schizophrenia

## Abstract

Accurate prognostication of individuals at clinical high-risk for psychosis (CHR-P) is an essential initial step for effective primary indicated prevention. We aimed to summarise the prognostic accuracy and clinical utility of CHR-P assessments for primary indicated psychosis prevention. Web of Knowledge databases were searched until 1st January 2022 for longitudinal studies following-up individuals undergoing a psychometric or diagnostic CHR-P assessment, reporting transition to psychotic disorders in both those who meet CHR-P criteria (CHR-P + ) or not (CHR-P−). Prognostic accuracy meta-analysis was conducted following relevant guidelines. Primary outcome was prognostic accuracy, indexed by area-under-the-curve (AUC), sensitivity and specificity, estimated by the number of true positives, false positives, false negatives and true negatives at the longest available follow-up time. Clinical utility analyses included: likelihood ratios, Fagan’s nomogram, and population-level preventive capacity (Population Attributable Fraction, PAF). A total of 22 studies (*n* = 4 966, 47.5% female, age range 12–40) were included. There were not enough meta-analysable studies on CHR-P diagnostic criteria (DSM-5 Attenuated Psychosis Syndrome) or non-clinical samples. Prognostic accuracy of CHR-P psychometric instruments in clinical samples (individuals referred to CHR-P services or diagnosed with 22q.11.2 deletion syndrome) was excellent: AUC = 0.85 (95% CI: 0.81–0.88) at a mean follow-up time of 34 months. This result was driven by outstanding sensitivity (0.93, 95% CI: 0.87–0.96) and poor specificity (0.58, 95% CI: 0.50–0.66). Being CHR-P + was associated with a small likelihood ratio LR + (2.17, 95% CI: 1.81–2.60) for developing psychosis. Being CHR-P- was associated with a large LR- (0.11, 95%CI: 0.06−0.21) for developing psychosis. Fagan’s nomogram indicated a low positive (0.0017%) and negative (0.0001%) post-test risk in non-clinical general population samples. The PAF of the CHR-P state is 10.9% (95% CI: 4.1–25.5%). These findings consolidate the use of psychometric instruments for CHR-P in clinical samples for primary indicated prevention of psychosis. Future research should improve the ability to rule in psychosis risk.

## Introduction

Reducing the duration of untreated psychosis [1] is a mainstream strategy to improve clinical outcomes. Primary indicated prevention in help-seeking young people displaying attenuated symptoms (at Clinical High-Risk for Psychosis, CHR-P) [2, 3] holds the greatest potential to reduce the duration of untreated psychosis [4]. The impact of the CHR-P paradigm is dependent on the accurate prognostication of their outcomes [5].

Unlike other areas of medicine where biological tests are available, CHR-P prognostication is entirely conducted through psychometric instruments such as the Comprehensive Assessment for At Risk Mental States (CAARMS) [6] and the Structured Interview for Psychosis Risk Syndromes (SIPS) [7] (for the assessment of Ultra High Risk [UHR] criteria [8]); and the Bonn Scale for the Assessment of Basic Symptoms (BSABS) [9] and Schizophrenia Proneness Instruments - Adult (SPI-A) [10] and Child & Youth (SPI-CY) [11] versions (for the assessment of Basic Symptom criteria) [12]. Furthermore, in 2013, diagnostic criteria for Attenuated Psychosis Syndrome were introduced to the DSM-5 (DSM-5-APS) [13] (for comparative analyses see [14] and eIntroduction).

In a previous meta-analysis (including studies until March 2015), we synthesised the prognostic accuracy of CHR-P instruments (*n* = 11 studies) as excellent (area-under-the-curve, AUC = 0.90, 95% CI: 0.87–0.93) [15]. Ever since, numerous new CHR-P prognostic accuracy studies have been published, making an update necessary. This is particularly essential given the recently updated transition risk in CHR-P individuals [16, 17] and new diagnostic criteria (DSM-5-APS) [14]. This study primarily aims to produce a prognostic accuracy meta-analysis for CHR-P assessments, complementing it with an investigation of its clinical utility.

## Methods

The study protocol was pre-registered and made publicly available on the PROSPERO database (CRD42021249341) and followed the Preferred Reporting Items for Systematic Reviews and Meta-analyses (PRISMA) 2020 reporting guidelines [18] (eTable 1), the Meta-analysis of Observational Studies in Epidemiology (MOOSE) 2000 reporting guidelines [19] (eTable 2).

### Search strategy

Two investigators (DO, MA) independently conducted a two-step literature search. As a first step, the Web of Knowledge database (Web of Science and MEDLINE) was searched from inception to 1st January 2022, using several combinations of the keywords reported in eMethods 1. The second step involved the use of Scopus to investigate citations of previous systematic reviews on transition outcomes in CHR-P samples and a manual search of the reference lists of the retrieved articles. The abstracts of articles identified were then screened for the selection criteria. The full-text articles surviving this selection were assessed for eligibility.

### Selection criteria

Studies were eligible for inclusion if they: (a) were reported in original articles, written in English; (b) had used an established CHR-P psychometric instrument as index test (UHR: CAARMS, SIPS, Brief Psychiatric Rating Scale (BPRS) [20], Basel Screening Instrument for Psychosis (BSIP) [21], Early Recognition Inventory (ERIraos) [22], Positive and Negative Syndrome Scale [23]; BS: BSABS, SPI-A/SPI-CY) or diagnostic criteria (DSM-5 APS); (c) had followed up both individuals meeting CHR-P criteria (CHR-P + ) and not (CHR-P−) using established international diagnostic manuals (ICD or DSM) or CHR-P psychometric criteria for psychosis onset (reference standard) and; (d) had reported sufficient prognostic accuracy data (i.e. transitions over time in CHR-P + and CHR-P− subjects). When data were not directly presented, corresponding authors were contacted.

We excluded: (a) abstracts, pilot datasets, reviews, articles in a language other than English; (b) studies in which CHR-P interviews were not conducted in the same pool of referrals or that used an external CHR-P- group of healthy controls; (c) studies with overlapping datasets. In case of overlapping samples, we selected the article reporting the largest and most recent dataset.

### Recorded variables

Data extraction was independently performed by two investigators (DO, MA). Data included author, year of publication, characteristics of subject samples (the predictor [index test], psychosis diagnosis [reference standard], age, gender [% females]), baseline exposure to antipsychotics, pre-screening, follow-up time, baseline number of CHR-P + and CHR-P− subjects, prognostic accuracy data (number of true and false positives, true and false negatives). Transition to psychosis was operationalised as defined by each study involving either CHR-P psychometric operationalisations or international diagnostic manuals (ICD/DSM, any version). Quality assessment was conducted independently by two investigators (DO, MA) with the Quality Assessment of Diagnostic Accuracy Studies 2 (QUADAS-2) checklist [24].

### Statistical analysis

The statistical analysis followed the Cochrane Guidelines for Systematic Reviews of Diagnostic Test Accuracy, Version 1.0 [25] and the Methods Guide for Authors of Systematic Reviews of Medical Tests by the Agency for Healthcare Research and Quality (chapter 8) [26].

#### Prognostic accuracy meta-analysis

For each study, we constructed a two-by-two table, which included true positive, false positive, true negative, and false negative values, using data from the longest follow-up. Drop-outs in each group (CHR-P + and CHR-P−) were assumed to have equal transition risk of non-drop-outs in those groups, following previously established methods [17] (but see sensitivity analyses) [27, 28]. Studies (a) using psychometric instruments (CHR-P) and diagnostic criteria (DSM-5 APS), and (b) with clinical and non-clinical samples [29] were analysed separately when at least three studies were available. The index tests and reference standards of transition to psychosis were dichotomous. Prognostic accuracy values of 0.9–1.0 are considered outstanding, of 0.8–0.9 excellent and of 0.7–0.8 acceptable [30] (see eMethods 2).

Sensitivity analyses were conducted: (1) to test the impact of variable follow-up times by stratifying the data at 6, 12, 24 and ≥30 months, (2) to estimate the effect of drop-out assumptions by 2a) excluding all drop-outs; 2b) assuming no drop-outs transitioned and; 2c) assuming all drop-outs transitioned, in line with our previous study [17], (3) to test the impact of single studies (leave-one-out analyses).

Heterogeneity across studies was assessed using the I^2^, with values of 25%, 50% and 75% representing mild, moderate and severe inconsistency, respectively [31]. Meta-regressions were used to examine the influence of known predictors: CHR-P instruments, mean age, gender (% females), follow-up time, sample size, baseline exposure to antipsychotics and use of pre-screening. Publication bias was investigated using Deeks’ funnel plot by conducting a sample size-weighted regression of the log odds ratio against the inverse of the square root of the sample size [26]. Meta-analytical Integration of Diagnostic Accuracy Studies (MIDAS) [32] package in STATA 14 was employed. Statistical tests were two-sided, and the threshold for statistical significance was *p* < 0.05.

#### Clinical utility

Studies (a) using psychometric instruments (CHR-P) and diagnostic criteria (DSM-5 APS); and (b) with clinical and non-clinical samples [29] were again analysed separately. We evaluated the positive and negative likelihood ratios (LR + and LR−) to calculate post-test probability (PostTP) based on Bayes’ theorem (with pre-test probability, PreTP, being the prevalence of the condition in the target population), as follows: PostTP = LR × PreTP/[(1−PreTP) + (PreTP × LR)] [33]. This is displayed through the probability-modifying plot [32] as a graphical sensitivity analysis. It depicts separate curves for positive and negative tests and uses general summary statistics (i.e. unconditional positive and negative predictive values, PPV and NPV, which permit underlying psychosis risk heterogeneity) to evaluate the prognostic utility of the index test [34]. The PreTP probability of psychosis risk was computed in the current dataset using random-effects meta-analysis with the metaprop function in the meta (version 4.15-1) package in R (version 3.6.3) as the proportion of subjects developing psychosis on the total baseline sample (CHR-P + plus CHR-P−) [32].

We also used Fagan’s nomogram, a two-dimensional graphical tool for estimating how much the result of a test changes the PreTP that a CHR-P + individual will develop psychosis. The PostTP was calculated using the LR + and LR− obtained from the current meta-analysis [35] and using the PreTP in the general population as estimated from the available literature [36].

Preventive capacity was assessed using the population attributable fraction (PAF) [37] of the CHR-P state, calculated from the prevalence of CHR-P individuals in the general population (estimated in a recent epidemiological meta-analysis [38]) and the relative risk of its association with psychosis onset. The latter was calculated using the current dataset and random-effects meta-analysis with the metabin function in the meta (version 4.15-1) package in R (version 3.6.3). PAF analysis was then performed using Levin’s formula [37]. Statistical tests were two-sided, and the threshold for statistical significance was *p* < 0.05.

## Results

### Database

**Fig. 1:**
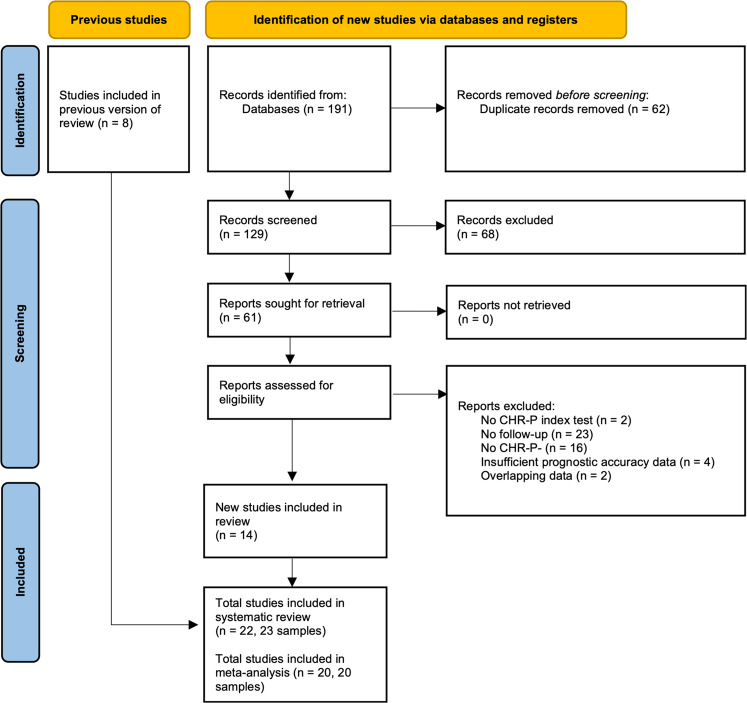
Study selection and inclusion for the current meta-analysis. *n* = 8 of the *n* = 11 studies from the previous meta-analysis were included in this analysis [53–60], with the other *n* = 3 samples [21, 42, 100] being replaced by more recent publications with larger overlapping samples and/or longer follow-up of the original sample [42, 46, 50].

A total of 14 independent studies reporting new data met inclusion criteria [39–52], in addition to 8 further independent studies [53–60] previously identified [15]. This resulted in 22 studies (23 samples, 4 966 individuals, CHR-P + = 2381; CHR-P− = 2 687, the proportion of CHR-P + = 47.9%, Fig. 1, Table 1), with 64% of studies contributing new data not previously analysed. 20 clinical samples [39–50, 53–60] contributed data on CHR-P psychometric instruments for a total of 4819 individuals (CHR-P + = 2333; CHR-P−= 2486, proportion of CHR-P + = 48%). These samples all consisted of individuals referred to CHR-P services or diagnosed with a 22q11.2 deletion syndrome. In terms of specific CHR-P psychometric instruments, seven samples were assessed with the CAARMS [42–44, 48, 50, 54, 59], eight the SIPS [39–41, 45, 47, 55–57], one used the BSIP [46], one the BSABS [53], and two used both the SIPS and SPI-A [58, 60]. One non-clinical sample [51] contributed data on CHR-P psychometric instruments (total *n* = 52; CHR-P + = 7; CHR-P− = 45; Table 1). Two samples [50, 52] contributed data on diagnostic criteria (total *n* = 354; CHR-P + = 161; CHR-P− = 193). Risk of bias and applicability concerns are shown in eTable 3 and eFig. 1.

**Table 1:** Studies included in systematic review.

Author	Country	Index test	Reference standard	Age, years (mean ± SD, range)	Gender (% females)	Baseline exposure to antipsychotics (%)	Pre-screening assessment	Follow-up time, months (longest)	CHR-P + subjects, *n*	CHR-P− subjects, *n*
Klosterkötter, 2001 [53]^a^	Germany	BSABS^d^	DSM-IV	29.3 ± 10.0 (15–53)	47.5	No	No	0, ≥30 (115)	110	50
Kobayashi, 2008 [39]	Japan	SIPS^e^	DSM-IV	23.6 ± 4.2 (NR)	59.7	NR	No	0, 6 (6)	19	94
Yung, 2008 [54]^a^	Australia	CAARMS^e^	CAARMS	18.1 (15–24)	51	Yes (NR)	No	0, 6, 24 (24)	119	173
Woods, 2009 [55]^a,b^	USA	SIPS^e^	DSM-IV	17.8 ± 4.4 (12–36)	39.5	Yes (11.6%)	No	0, 6, 12, 24, ≥30 (37)	259	111
Liu, 2011 [56]^a^	Taiwan	SIPS^e^	DSM-IV	21.4 ± 4.0 (16–24)	47.7	Yes (79.7%)	No	0, 24 (24)	59	48
Addington, 2012 [57]^a,b^	Canada	SIPS^e^	DSM-IV	19.8 ± 4.5 (12–31)	47.8	Yes (1.8%)	No	0, 6, 12, 24, ≥30 (37)	172	100
Simon, 2012 [58]^a^	Switzerland	SIPS/SPI-A^d^	DSM-IV	21.0 (14–40)	32.4	No	No	0, 12, 24 (24)	99	49
Lee, 2013 [59]^a,b^	Singapore	CAARMS^e^	DSM-IV	21.6 ± 3.5 (14–29)	39.9	No	No	0, 6, 12, 24, ≥30 (30)	173	494
Lindgren, 2014 [40]	Finland	SIPS^e^	DSM-IV	16.6 ± 0.9 (15–18)	78.9	NR	No	0, 12, ≥30 (60)	54	97
Schultze-Lutter, 2014 [60]^a^	Switzerland	SIPS/SPI-A^d,e^	DSM-IV	24.9 ± 6.0 (15–39)	37	Yes (13.8%)	No	0, 6, 12, 24, ≥30 (48)	194	52
Kline, 2015 [41]	USA	SIPS^e^	SIPS	16.2 ± 3.1 (12–24)	65	NR	No	0, 6 (6)	21	33
Kotlicka-Antczak, 2015 [42]^a,b^	Poland	CAARMS^e^	ICD-10	18.8 ± 3.6 (15–29)	49.2	Yes (10.8%)	No	0, ≥30 (46)	122	92
Francesconi, 2017 [43]	Italy	CAARMS^e^	CAARMS	24.3 ± 3.5 (NR)	47	No	No	0, 12 (12)	67	71
Fusar-Poli, 2017 [44]	UK	CAARMS^e^	ICD-10	23.0 ± 5.4 (12–44)	43.8	No	No	0, 6, 12, 24, ≥30 (36)	402	299
Masillo, 2018 [45]	Italy	SIPS^e^	DSM-IV	16.4 ± 5.1 (NR)	47.1	No	Yes	0, 18 (18)	29	55
Papmeyer, 2018 [46]	Switzerland	BSIP^e^	BPRS	27.6 ± 8.1 (NR)	42.9	No	No	0, 24 (24)	117	39
Xu, 2018 [47]	China	SIPS^e^	SIPS	22.9 ± 6.6 (NR)	57.1	No	Yes	0, 24 (24)	112	345
Pelizza, 2019 [48]	Italy	CAARMS^e^	CAARMS	19.3 ± 5.8 (NR)	45.4	No	No	0, 12 (12)	55	64
Schneider, 2019 [49]	Italy, Switzerland	SIPS^e^	DSM-IV	15.7 ± 4.7 (8–33)	53.2	Yes (6.3%)	No	0, ≥30 (33)	30	81
Mensi, 2021 [50]^b,c^	Italy	CAARMS^e^	DSM-V	15.4 ± 5.9 (12–17)	64.2	Yes (4.7%)	No	0, 6, 12, 24, ≥30 (72)	110	102
Manninen, 2014 [51]^f^	Finland	SIPS^e^	ICD-10	NR (15–18)	59.7	NR	No	0, ≥30 (60)	7	45
Fusar-Poli, 2018 [52]	UK	DSM-5 APS^g^	CAARMS	25.0 ± 5.4 (NR)	35.9	No	No	0, 3, 6, 12, 18, 24 (24)	51	91
Mensi, 2021 [50]^b,c^	Italy	DSM-5 APS^g^	DSM-V	15.4 ± 5.9 (12–17)	64.2	Yes (4.7%)	No	0, 6, 12, 24, ≥30 (72)	110	102

*BPRS* Brief Psychiatric Rating Scale, *BSABS* Bonn Scale for the Assessment of Basic Symptoms, *BSIP* Basel Screening Instrument for Psychosis, *CAARMS* Comprehensive Assessment for At-Risk Mental States, *CHR-P* clinical high risk for psychosis, *POPS* Presence of Psychotic Syndrome, *SIPS* Structured Interview for Prodromal Syndromes, *SPI-A* Schizophrenia Proneness Instrument, *CHR-P* clinical high risk for psychosis, *NR* not reported.

^a^Included in previous meta-analysis [15].

^b^Author submitted additional data.

^c^Study used both psychometric and clinical instruments.

^d^Basic Symptoms (BS) Criteria.

^e^Ultra High Risk (UHR) Criteria.

^f^Non-clinical sample.

^g^Diagnostic criteria.

### Prognostic accuracy of CHR-P psychometric instruments

**Fig. 2:**
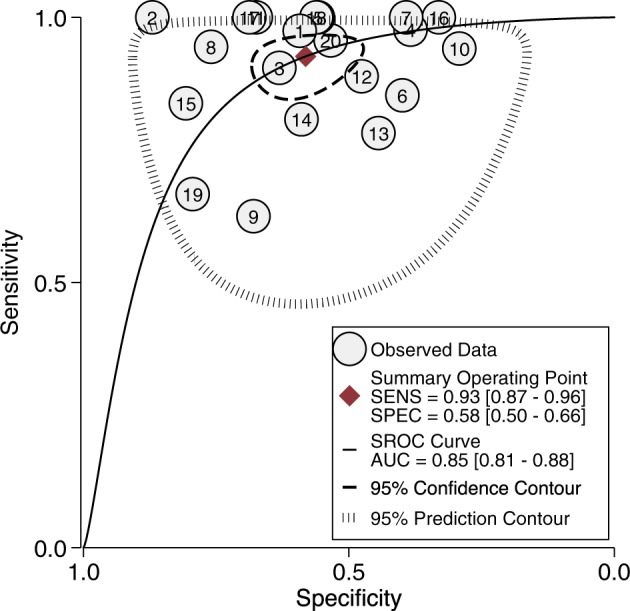
Meta-analytical summary receiver operating characteristic (SROC) curve. Summarises the prognostic accuracy of clinical high risk for psychosis (CHR-P) psychometric instruments in clinical samples at an average follow-up time of 34 months. N.B. *x*-axis for Sp runs reversed. Se – sensitivity, Sp – specificity, AUC – area under the curve, 1 – Klosterkötter et al. [53], 2 - Kobayashi et al. [39], 3 – Yung et al. [54], 4 – Woods et al. [55], 5– Liu et al. [56], 6 – Addington et al. [57], 7 – Simon et al. [58], 8 – Lee et al. [59], 9 – Lindgren et al. [40], 10 – Schultze-Lutter et al. [60], 11 – Kline et al. [41], 12 – Kotlicka-Antczak et al. [42], 13 - Fusar-Poli et al. [44], 14 – Francesconi et al. [43], 15 – Pelizza et al. [48], 16 – Xu et al. [47], 17 – Papmeyer et al. [46], 18 - Masillo et al. [45], 19 – Schneider et al. [49], 20 - Mensi et al. [50].

Across the 20 clinical samples employing CHR-P psychometric instruments [39–50, 53–60], the meta-analytical prognostic accuracy was excellent for AUC 0.85 (95% CI: 0.81–0.88), outstanding for sensitivity (Se) 0.93 (95% CI: 0.87–0.96), while specificity (Sp) was poor: 0.58 (95%CI: 0.50–0.66; Fig. 2, eFig. 2) at a mean follow-up time of 34.4 months (SD = 25.5, median = 27.0). There was severe heterogeneity for Se (*I*^2^ = 79.9, 95% CI: 71.6–88.1) and Sp (*I*^2^ = 96.1, 95% CI: 95.1–97.1), 18% of which was due to threshold effects. Model diagnostics revealed a good fit of the model, with one study [39] reaching the high influence and outlier thresholds (eFig. 3, eFig. 4). There was no significant evidence of small study effects (*p* = 0.54; eFig. 5). Sensitivity analyses addressed the impact of follow-up time at 6, 12, 24 and ≥30 months (eResults 1, eTable 4) and drop-out assumptions (eTable 4, eFig. 6); the overall estimates were not substantially influenced by single studies (eTable 5). There were not sufficient studies to meta-analyse diagnostic criteria or non-clinical samples.

Meta-regression showed no significant effects of age, gender, follow-up time, sample size, baseline exposure to antipsychotics, pre-screening (eFig. 7) or CHR-P instruments (SIPS vs. CAARMS, eFig. 8).

### Clinical utility of CHR-P psychometric instruments

**Fig. 3:**
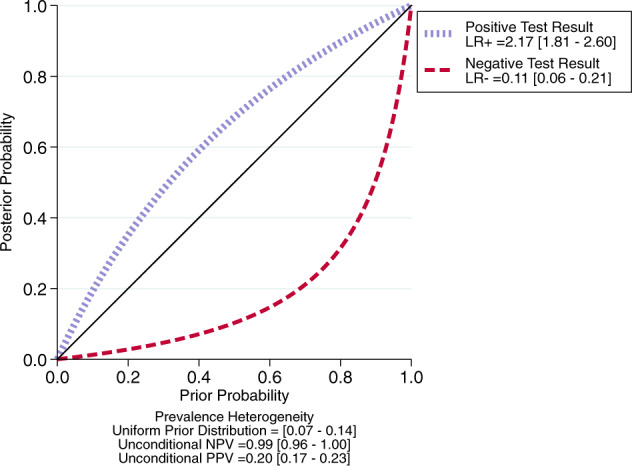
Meta-analytical probability-modifying plot. This plot llustrates the relationship between pre-test probability (PreTP) (6.7–14.1% psychosis risk at 34 months in clinical samples) and post-test probability (PostTP) (psychosis risk at 34 months in clinical samples based on clinical high risk psychometric interviews), computed as the likelihood of a positive (above diagonal line; LR + ) or negative (below diagonal line, LR−) test result over the 0–1 range of PreTP.

The 34-month psychosis risk in the 4819 subjects in clinical samples tested with CHR-P psychometric instruments was 9.8% (95% CI: 6.7–14.1%). The continuous relationship between PreTP and PostTP is summarised in Fig. 3. Being CHR-P + was associated with a 19.7% (95% CI: 14.6–26.1%) risk of developing psychosis within 34 months, yet a small LR + of 2.17 (95% CI: 1.81–2.60), while being CHR-P- was associated with a 1.5% (95% CI: 0.8–2.7%) risk of developing psychosis and a large LR- of 0.11 (95% CI: 0.06–0.21; Fig. 3).

**Fig. 4:**
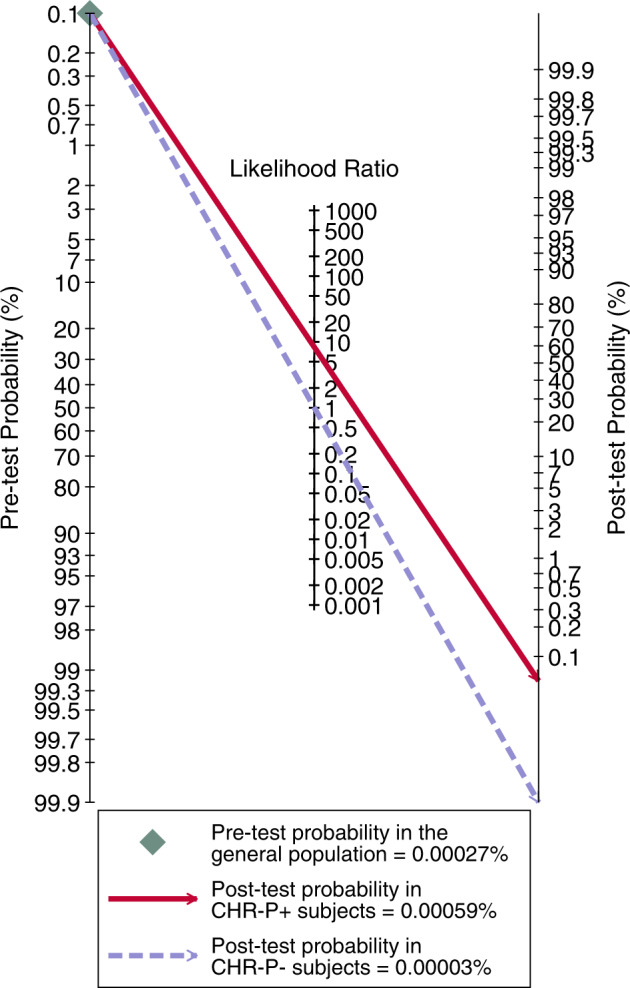
Fagan’s nomogram. This plot illustrates the meta-analytical clinical value (post-test probability) of clinical high risk for psychosis (CHR-P) psychometric instruments in order to predict risk of psychosis at 34 months in the general population.

Based on an annualised incidence of all non-organic psychotic disorders of 0.00027% [36] (resulting in an incidence over 34 months of 0.00077%) and the above LRs, Fagan’s nomogram revealed only limited clinical utility for CHR-P psychometric instruments in the general population (Fig. 4). Testing positive for CHR-P was associated with a 0.0017% risk of developing psychosis within 34 months, while testing negative was associated with extremely low risk (0.0001%).

### Preventive capacity of CHR-P psychometric instruments

Based on the meta-analytic prevalence of the CHR-P state in the general population [38] (1.7%, 95% CI: 1.0–2.9%) and the risk ratio associated with CHR-P + individuals for psychosis onset calculated from the current dataset (RR = 8.22, 95% CI: 5.28–12.80), the PAF of the CHR-P state, ascertained with psychometric instruments, is 10.9% (95% CI: 4.1–25.5%).

## Discussion

This study presents the most up-to-date and well-powered meta-analytical estimate of the prognostic accuracy of CHR-P psychometric instruments and diagnostic criteria for primary indicated prevention of psychotic disorders. Using CHR-P psychometric instruments to assess the CHR-P state in clinical samples, including those referred to high-risk services or diagnosed with 22q.11.2 deletion syndrome, is associated with an excellent overall prognostic performance. There is only emerging evidence on the DSM-5-APS. CHR-P psychometric instruments show clinical utility in clinical populations but not in the general population.

The primary aim of this study was reached by meta-synthesising the available evidence to estimate the prognostic accuracy of CHR-P psychometric instruments in clinical samples, either referred to CHR-P services or diagnosed with 22q.11.2 deletion syndrome. CHR-P services are increasingly being implemented worldwide with a growing testing capacity [61, 62]. The prognostic performance of CHR-P psychometric instruments was ascertained in the long-term (at 34 months), showing an excellent AUC = 0.85. The overall AUC value is comparable to other risk assessment tools based on sociodemographic or questionnaire data used in somatic medicine [63]. However, the AUC was unbalanced and while sensitivity was high (0.93), specificity was inadequate (0.58) indicating a need to improve specificity in future research. The solid prognostic accuracy of CHR-P psychometric instruments may partially originate from the extensive training required to administer them and indicates that forecasting the onset of psychosis in clinical samples is possible [64, 65]. This achievement represents one of the few successful implementations of prognostic medicine in psychiatry [66], a field that is characterised by a replication crisis [67–69] and profound translational gaps [70].

Our findings additionally support the prognostic validity of CHR-P psychometric assessment in individuals affected with 22q11.2 deletion syndrome [49], which represents the most solid genetic biomarker of an impending psychosis risk to date. We previously validated Fagan’s nomogram in 22q11.2 deletion syndrome samples, confirming the clinical utility of testing these individuals [71]. Approximately 27% of individuals with 22q11.2 deletion syndrome meet CHR-P criteria with psychometric instruments [49, 72], compared to 1.7% in the general population [38] and 19.2% in clinical populations [38]. Psychotic disorders are present in up to 41% of adults with 22q11.2 deletion syndrome [73].

However, the Se and Sp are unbalanced in CHR-P psychometric instruments, with Se being 0.36 higher than Sp, compared to a difference of 0.14 between Se and Sp in the other somatic medicine prognostic assessments such as the Cambridge Risk Score for diabetes [63]. There is, therefore, a clear need to focus efforts on improving the ability of these instruments to rule in psychosis (i.e. increase Sp and LR + ) while maintaining their outstanding ability of ruling out psychosis (i.e. high Se and low LR-). This limitation is in part due to the intrinsic inability to refine the current group-level prognostic estimates beyond the subgroup stratification (APS, BLIPS or GRD) [74]. To refine estimates to the individual level, CHR-P psychometric instruments should be supplemented with information from other modalities beyond symptomatology (e.g. proteomics [75], neuroimaging [76] and clinical/neurocognitive [77] data). Symptoms are not the underlying cause of psychosis but are instead epiphenomena of underlying gene-by-environment interactions [78]. Genetic and environmental factors are therefore more closely linked to aetiopathology and may be more robust indicators of underlying psychosis risk. For example, the assessment of environmental risk and protective factors (e.g. Psychosis Polyrisk Score [PPS]) [79, 80] could integrate the CHR-P testing and mitigate these issues by addressing underlying aetiopathology [79, 80]. Longitudinal, multisite studies through international consortia are key to providing the platform for this [81, 82].

There is also high heterogeneity in recruitment strategies for high-risk services, and therefore PreTP and transition risk [17, 29]. Extensive outreach campaigns lead to more individuals with negligible psychosis risk being assessed, thereby diluting PreTP and subsequently PostTP [29]. Methods to enrich the PreTP of samples assessed with CHR-P psychometric instruments would have a significant impact on increasing PostTP [28, 83], improving Sp and global prognostic accuracy. This can be achieved through several different strategies that can be performed in isolation or in combination, focusing on the community, primary care and secondary mental healthcare [84]. Firstly, our results have shown that assessing an un-enriched community sample has low clinical utility. Instead, self-report pre-screening tools assessing psychotic-like symptoms (e.g. Prodromal Questionnaire (PQ-16) [85] or the PRIME Screen – Revised) [47] can identify individuals who have an enriched psychosis risk to be assessed with CHR-P psychometric instruments. Secondly, while primary care is a common source of referrals for assessment with CHR-P psychometric instruments [86], many general practitioners are not familiar or confident with recognising the CHR-P state [87]. While use of CHR-P psychometric instruments as a systematic screening method to all individuals accessing primary care settings is logistically untenable and psychometrically not desirable due to the modest pre-test risk enrichment [28, 79], an alternative may be to leverage automated individualised risk calculators based on electronic health records to support referral decisions from primary care while retaining risk enrichment [88, 89]. Following this initial screening, patients detected could be assessed with CHR-P psychometric instruments in a specialised psychiatric setting to validate the presence of at-risk symptoms. Thirdly, automated screening of electronic health records based on readily available information could similarly aid the identification of individuals at-risk already accessing secondary mental healthcare. Clinically-based, individualised, automated, transdiagnostic risk calculator for psychosis in secondary mental healthcare with good performance has been developed [90], replicated across several national [90–92] and international [93] replications, and already implemented in clinical routine [70, 94, 95].

The clinical utility of psychometric CHR-P instruments is similarly predicated on enriching PreTP, as shown by the low PostTPs following their use in general population samples. Regardless of the outcome of the assessment, the risk of an individual in the 3 years following is negligible. However, when used in clinical samples, either from high-risk services or with 22q11.2 deletion syndrome, whose PreTP is enriched but less certain, the preventive capacity of these instruments is relatively high. We updated our recent PAF meta-analysis by showing that if the risk of developing psychosis from a CHR-P state was completely eradicated, 10.9% of psychosis cases in the population would be prevented. It is important to acknowledge that this estimate is only representing a hypothetical ideal scenario, which assumes complete detection of CHR-P cases and preventive interventions that can fully abate the likelihood of developing psychosis in CHR-P individuals. Currently, both detection and effective prevention of psychosis in the CHR-P field remain suboptimal [69, 96, 97].

This study has some limitations. Firstly, we could not conduct a meta-analysis of prognostic accuracy on diagnostic criteria (i.e. DSM-5-APS) because there were only two eligible studies (eDiscussion) [50, 52]. While transition risk in those meeting DSM-5-APS criteria are well reported, the risk of developing psychosis among those testing negative on these criteria should be better addressed by future research [14]. Furthermore, the follow-up times of the included studies varied. However, there was no significant effect of follow-up time through meta-regression; interestingly, our mean follow-up time of 34 months coincides with the start of the plateau in psychosis risk recently reported [98]. Despite this plateauing, risk continues to increase up to 36.5% at 10 and 11 years [99]: future research should investigate the long-term prognostic accuracy of CHR-P assessments.

This updated meta-analysis of prognostic accuracy consolidates the use of psychometric instruments for CHR-P for primary indicated prevention of psychosis in individuals referred to CHR-P services or with 22q.11.2 deletion syndrome. Future research should improve ability to rule in psychosis risk.

## Supplementary information

Supplementary
