# Supplementary material for: Prognostic accuracy and clinical utility of psychometric instruments for individuals at clinical high-risk of psychosis: a systematic review and meta-analysis
Source: Mol Psychiatry. 2022 Jun 3;27(9):3670–8. doi: 10.1038/s41380-022-01611-w (PMC9708585; doi:10.1038/s41380-022-01611-w)
Supplement: Supplementary file 1 — Supplementary [file 41380_2022_1611_MOESM1_ESM.docx]

**Supplementary Online Content**

Oliver D, Arribas M, Radua J, et al. Prognostic accuracy and clinical utility of psychometric instruments for individuals at clinical high risk of psychosis: A systematic review and meta-analysis

**eIntroduction** Differences in operationalisation of CHR-P and DSM-5-APS criteria

**eTable 1** PRISMA checklist

**eTable 2** MOOSE checklist

**eMethods 1** Electronic search

**eMethods 2** Prognostic accuracy meta-analysis methods

**eTable 3** Individual study-level scores for risk of bias and applicability concerns as measured by QUADAS-2

**eFigure 1** Summary of risk of bias and applicability concerns as measured by QUADAS-2

**eFigure 2** Forest plot showing study-specific and meta-analytic summary sensitivity (Se) and specificity (Sp) with corresponding heterogeneity statistics

**eFigure 3** Model diagnostics

**eFigure 4** SROC with outliers removed

**eFigure 5** Deeks’ funnel plot for small-study effects/publication bias

**eResults 1** Sensitivity analyses assessing impact of follow-up time

**eTable 4** Sensitivity analyses for different assumptions regarding drop-outs

**eFigure 6** Sensitivity analyses for different assumptions regarding drop-outs

**eTable 5** Leave-one-out sensitivity analyses

**eFigure 7** Meta-regression results for age, gender, follow-up time, sample size, antipsychotics and use of pre-screening instruments

**eFigure 8** Meta-regression results for CHR-P psychometric instrument

**eDiscussion** Prognostic accuracy in DSM-5-APS samples

**eReferences**

**eIntroduction** Differences in operationalisation of CHR-P and DSM-5-APS criteria

The two most important distinctions are that: 1) while CHR-P assessments are semi-structured psychometric interviews, DSM-5-APS criteria are ascertained through an unstructured clinical assessment, similar to any other psychiatric diagnosis [1]; and 2) CHR-P criteria include individuals meeting criteria for attenuated psychotic symptoms (APS), brief limited intermittent psychosis syndrome (BLIPS) and genetic risk and/or deterioration (GRD) subgroups [2]. There is substantial heterogeneity between these three subgroups in terms of transition risk, with BLIPS criteria being associated with the highest risk (38% within 4 years), followed by APS (24%) and GRD with the lowest risk (8%) [3]. DSM-5-APS only aligns with CHR-P-defined APS, therefore disregarding both the highest and lowest transition risk groups, which likely has an impact on its inherent prognostic accuracy. At the time of publication of our earlier meta-analysis [4], no studies were available to ascertain the prognostic accuracy of DSM-5-APS.

| **eTable 1** PRISMA checklist | | | |
| --- | --- | --- | --- |
| **Section and Topic** | **Item #** | **Checklist item** | **Location where item is reported** |
| **TITLE** | | |  |
| Title | 1 | Identify the report as a systematic review. | 1 |
| **ABSTRACT** | | |  |
| Abstract | 2 | See the PRISMA 2020 for Abstracts checklist. | 3 |
| **INTRODUCTION** | | |  |
| Rationale | 3 | Describe the rationale for the review in the context of existing knowledge. | 4 |
| Objectives | 4 | Provide an explicit statement of the objective(s) or question(s) the review addresses. | 4 |
| **METHODS** | | |  |
| Eligibility criteria | 5 | Specify the inclusion and exclusion criteria for the review and how studies were grouped for the syntheses. | 5 |
| Information sources | 6 | Specify all databases, registers, websites, organisations, reference lists and other sources searched or consulted to identify studies. Specify the date when each source was last searched or consulted. | 5 |
| Search strategy | 7 | Present the full search strategies for all databases, registers and websites, including any filters and limits used. | eMethods 1 |
| Selection process | 8 | Specify the methods used to decide whether a study met the inclusion criteria of the review, including how many reviewers screened each record and each report retrieved, whether they worked independently, and if applicable, details of automation tools used in the process. | 5 |
| Data collection process | 9 | Specify the methods used to collect data from reports, including how many reviewers collected data from each report, whether they worked independently, any processes for obtaining or confirming data from study investigators, and if applicable, details of automation tools used in the process. | 5-6 |
| Data items | 10a | List and define all outcomes for which data were sought. Specify whether all results that were compatible with each outcome domain in each study were sought (e.g. for all measures, time points, analyses), and if not, the methods used to decide which results to collect. | 5-6 |
|  | 10b | List and define all other variables for which data were sought (e.g. participant and intervention characteristics, funding sources). Describe any assumptions made about any missing or unclear information. | 5-6 |
| Study risk of bias assessment | 11 | Specify the methods used to assess risk of bias in the included studies, including details of the tool(s) used, how many reviewers assessed each study and whether they worked independently, and if applicable, details of automation tools used in the process. | 6, eTable 3, eFigure 1 |
| Effect measures | 12 | Specify for each outcome the effect measure(s) (e.g. risk ratio, mean difference) used in the synthesis or presentation of results. | 6-7 |
| Synthesis methods | 13a | Describe the processes used to decide which studies were eligible for each synthesis (e.g. tabulating the study intervention characteristics and comparing against the planned groups for each synthesis (item #5)). | 6-7 |
|  | 13b | Describe any methods required to prepare the data for presentation or synthesis, such as handling of missing summary statistics, or data conversions. | N/A |
|  | 13c | Describe any methods used to tabulate or visually display results of individual studies and syntheses. | 6-7 |
|  | 13d | Describe any methods used to synthesize results and provide a rationale for the choice(s). If meta-analysis was performed, describe the model(s), method(s) to identify the presence and extent of statistical heterogeneity, and software package(s) used. | 6-7, eMethods 2 |
|  | 13e | Describe any methods used to explore possible causes of heterogeneity among study results (e.g. subgroup analysis, meta-regression). | 6 |
|  | 13f | Describe any sensitivity analyses conducted to assess robustness of the synthesized results. | 6 |
| Reporting bias assessment | 14 | Describe any methods used to assess risk of bias due to missing results in a synthesis (arising from reporting biases). | N/A |
| Certainty assessment | 15 | Describe any methods used to assess certainty (or confidence) in the body of evidence for an outcome. | 6-7 |
| **RESULTS** | | |  |
| Study selection | 16a | Describe the results of the search and selection process, from the number of records identified in the search to the number of studies included in the review, ideally using a flow diagram. | 8, Figure 1 |
|  | 16b | Cite studies that might appear to meet the inclusion criteria, but which were excluded, and explain why they were excluded. | N/A |
| Study characteristics | 17 | Cite each included study and present its characteristics. | 8, Table 1 |
| Risk of bias in studies | 18 | Present assessments of risk of bias for each included study. | eTable 3 |
| Results of individual studies | 19 | For all outcomes, present, for each study: (a) summary statistics for each group (where appropriate) and (b) an effect estimate and its precision (e.g. confidence/credible interval), ideally using structured tables or plots. | 8, Figure 2, eFigure 2 |
| Results of syntheses | 20a | For each synthesis, briefly summarise the characteristics and risk of bias among contributing studies. | Table 1, eTable 3 |
|  | 20b | Present results of all statistical syntheses conducted. If meta-analysis was done, present for each the summary estimate and its precision (e.g. confidence/credible interval) and measures of statistical heterogeneity. If comparing groups, describe the direction of the effect. | 8-9 |
|  | 20c | Present results of all investigations of possible causes of heterogeneity among study results. | 9, eFigure 7, eFigure 8 |
|  | 20d | Present results of all sensitivity analyses conducted to assess the robustness of the synthesized results. | 8, eFigure 4-6, eTable 4-5 |
| Reporting biases | 21 | Present assessments of risk of bias due to missing results (arising from reporting biases) for each synthesis assessed. | eTable 3, eFigure 1 |
| Certainty of evidence | 22 | Present assessments of certainty (or confidence) in the body of evidence for each outcome assessed. | N/A |
| **DISCUSSION** | | |  |
| Discussion | 23a | Provide a general interpretation of the results in the context of other evidence. | 9 |
|  | 23b | Discuss any limitations of the evidence included in the review. | 12 |
|  | 23c | Discuss any limitations of the review processes used. | 12 |
|  | 23d | Discuss implications of the results for practice, policy, and future research. | 9-12 |
| **OTHER INFORMATION** | | |  |
| Registration and protocol | 24a | Provide registration information for the review, including register name and registration number, or state that the review was not registered. | 4 |
|  | 24b | Indicate where the review protocol can be accessed, or state that a protocol was not prepared. | 4 |
|  | 24c | Describe and explain any amendments to information provided at registration or in the protocol. | 4 |
| Support | 25 | Describe sources of financial or non-financial support for the review, and the role of the funders or sponsors in the review. | 12 |
| Competing interests | 26 | Declare any competing interests of review authors. | 12 |
| Availability of data, code and other materials | 27 | Report which of the following are publicly available and where they can be found: template data collection forms; data extracted from included studies; data used for all analyses; analytic code; any other materials used in the review. | N/A |

| **eTable 2** MOOSE checklist | | |
| --- | --- | --- |
| **Item No** | **Recommendation** | **Reported on Page No** |
| Reporting of background should include | | |
| 1 | Problem definition | 4 |
| 2 | Hypothesis statement | - |
| 3 | Description of study outcome(s) | 5 |
| 4 | Type of exposure or intervention used | N/A |
| 5 | Type of study designs used | 5 |
| 6 | Study population | 5 |
| Reporting of search strategy should include | | |
| 7 | Qualifications of searchers (eg, librarians and investigators) | 5, Title page |
| 8 | Search strategy, including time period included in the synthesis and key words | 5, eMethods 1 |
| 9 | Effort to include all available studies, including contact with authors | 5 |
| 10 | Databases and registries searched | 5 |
| 11 | Search software used, name and version, including special features used (eg, explosion) | N/A |
| 12 | Use of hand searching (eg, reference lists of obtained articles) | 5 |
| 13 | List of citations located and those excluded, including justification | Table 1, Figure 1 |
| 14 | Method of addressing articles published in languages other than English | 5 |
| 15 | Method of handling abstracts and unpublished studies | 5 |
| 16 | Description of any contact with authors | 5 |
| Reporting of methods should include | | |
| 17 | Description of relevance or appropriateness of studies assembled for assessing the hypothesis to be tested | 4-7 |
| 18 | Rationale for the selection and coding of data (eg, sound clinical principles or convenience) | 4-7 |
| 19 | Documentation of how data were classified and coded (eg, multiple raters, blinding and interrater reliability) | 4-7 |
| 20 | Assessment of confounding (eg, comparability of cases and controls in studies where appropriate) | 6-7 |
| 21 | Assessment of study quality, including blinding of quality assessors, stratification or regression on possible predictors of study results | 6 |
| 22 | Assessment of heterogeneity | 6 |
| 23 | Description of statistical methods (eg, complete description of fixed or random effects models, justification of whether the chosen models account for predictors of study results, dose-response models, or cumulative meta-analysis) in sufficient detail to be replicated | 6-7 |
| 24 | Provision of appropriate tables and graphics | Figure 2-4, eFigure 1-8, Table 1, eTable 3-5 |
| Reporting of results should include | | |
| 25 | Graphic summarizing individual study estimates and overall estimate | Figure 2, eFigure 2 |
| 26 | Table giving descriptive information for each study included | Table 1 |
| 27 | Results of sensitivity testing (eg, subgroup analysis) | eTable 4-5, eFigure 6-8, eResults 1 |
| 28 | Indication of statistical uncertainty of findings | 12 |
| Reporting of discussion should include | | |
| 29 | Quantitative assessment of bias (eg, publication bias) | eFigure 5 |
| 30 | Justification for exclusion (eg, exclusion of non-English language citations) | 5 |
| 31 | Assessment of quality of included studies | eTable 3, eFigure 1 |
| Reporting of conclusions should include | | |
| 32 | Consideration of alternative explanations for observed results | 9-12 |
| 33 | Generalization of the conclusions (ie, appropriate for the data presented and within the domain of the literature review) | 9 |
| 34 | Guidelines for future research | 10-11 |
| 35 | Disclosure of funding source | 12 |

**eMethods 1** Electronic search keywords

“at risk mental state”, “psychosis risk”, “prodrome”, “prodromal psychosis”, "clinical high risk", “ultra-high risk”, “high risk”, "UHR", "CHR", "CHR-P", “DSM-5-APS”, “DSM-5 attenuated psychosis syndrome”, “help-seeking”, “diagnostic accuracy”, “sensitivity”, “specificity”, “psychosis prediction”, “psychosis onset”

**eMethods 2** Prognostic accuracy meta-analysis methods

Data synthesis was performed fitting advanced hierarchical random effects [5]. Specifically, the bivariate mixed-effects regression framework for the logit transforms of Se and Sp was employed [6] to account for study size, and to incorporate the intrinsic negative correlation that may arise between Se and Sp within studies (threshold effect) [7], as a result of differences in the test threshold between studies [8]. The bivariate model also allows for heterogeneity beyond chance as a result of clinical and methodological differences between studies [8]. As a global measure of prognostic accuracy, we estimated the AUC, a global measure of test performance. Values in the range of 0.9–1 are considered outstanding, between 0.8 and 0.9 excellent, between 0.7 and 0.8 acceptable [9]. We also estimated the summary Se and Sp and the hierarchical Summary Receiver Operating Characteristic (SROC) curves [5, 10], plotting a 95% confidence region and a 95% prediction region around the summary estimates (confidence ellipse of a mean), and showing the amount of between-study variation (prediction ellipse; the likely range of values for a new study).

|  |  |  |  |  |  |  |  |  |
| --- | --- | --- | --- | --- | --- | --- | --- | --- |
| **eTable 3** Individual study-level scores for risk of bias and applicability concerns as measured by QUADAS-2 | | | | | | | | |
|  | | **Risk of bias** | | | | **Applicability concerns** | | |
| **Author** | | **Patient selection** | **Index test** | **Reference standard** | **Flow and timing** | **Patient selection** | **Index test** | **Reference standard** |
| **PSYCHOMETRIC INSTRUMENTS** | | | | | | | | |
| **CLINICAL SAMPLES** | |  |  |  |  |  |  |  |
| **Klosterkötter, 2001** [11] | | 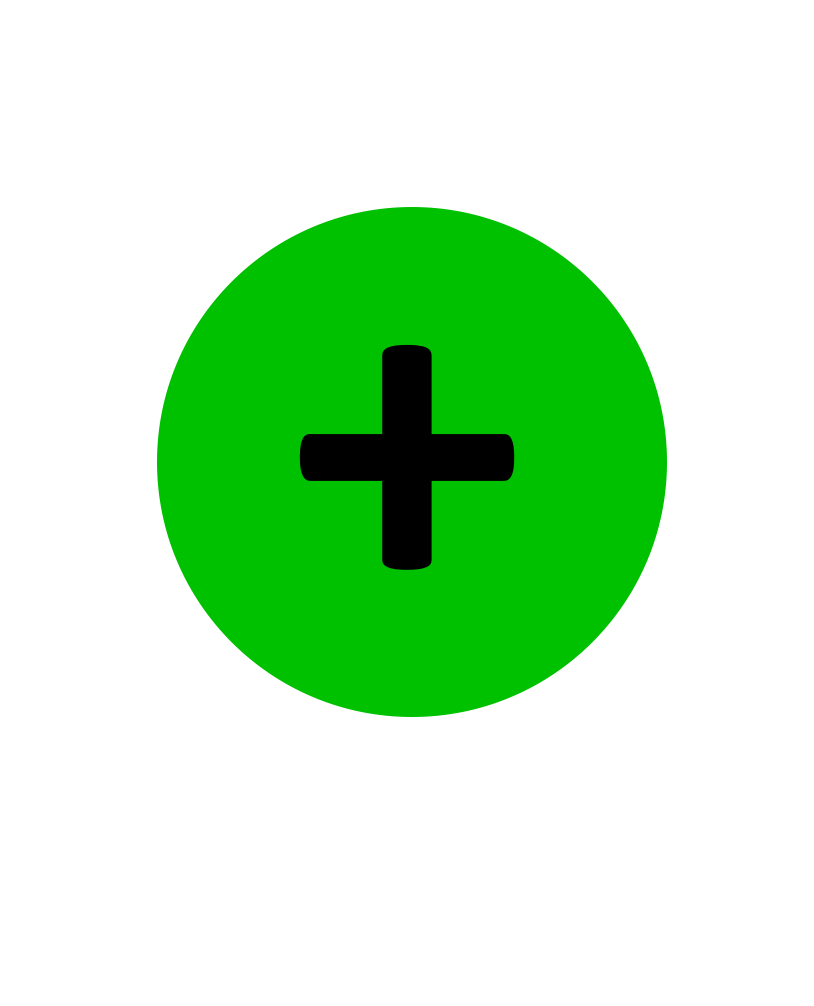 | 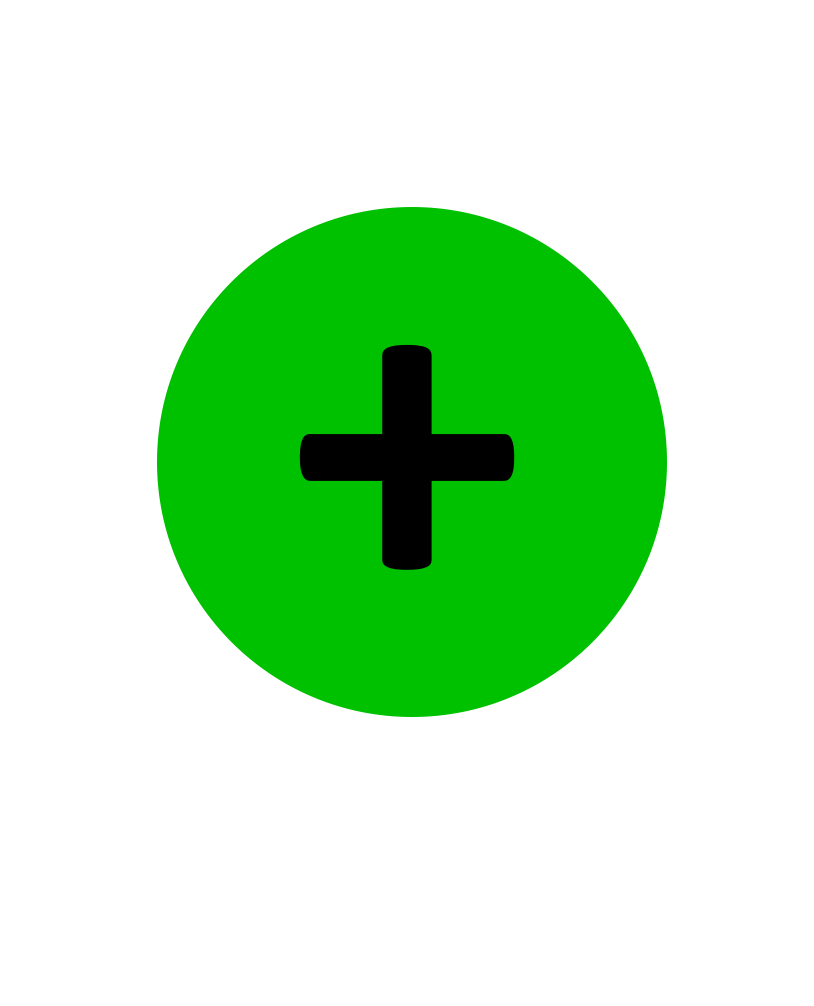 | 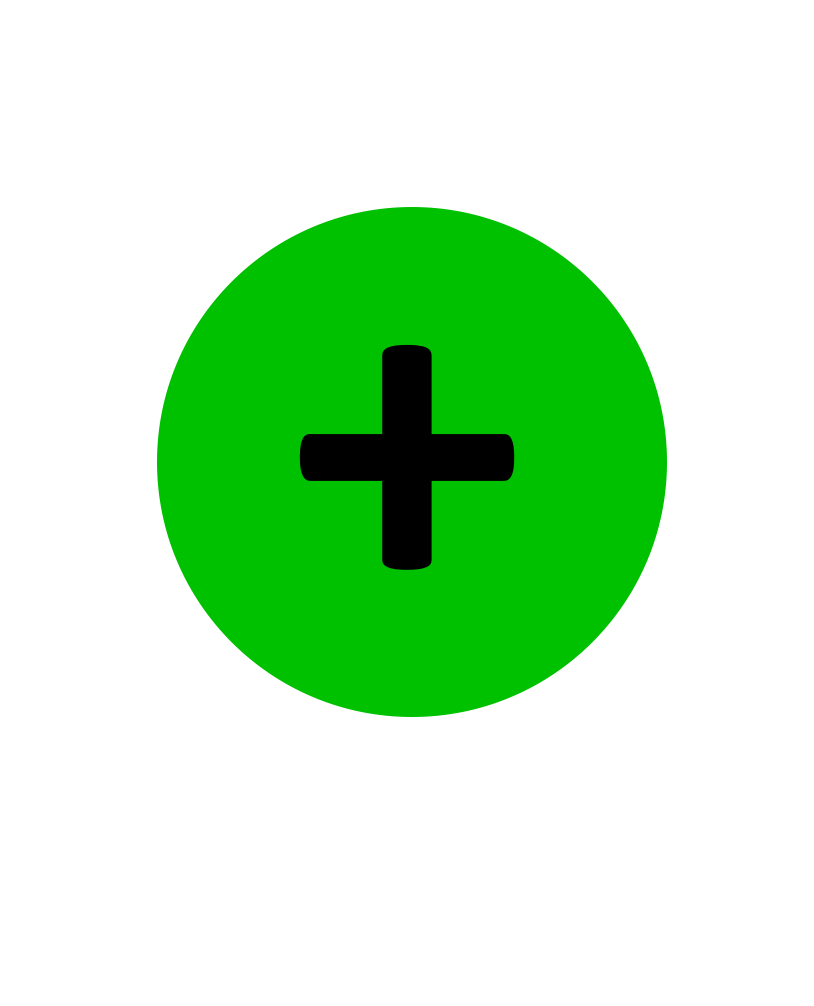 | 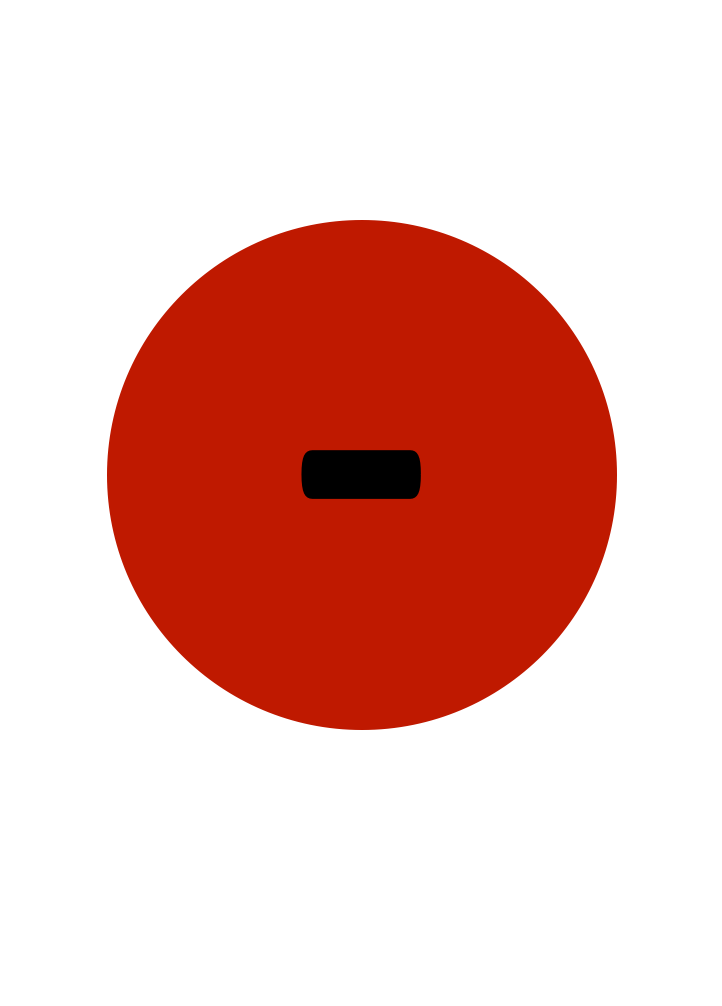 | 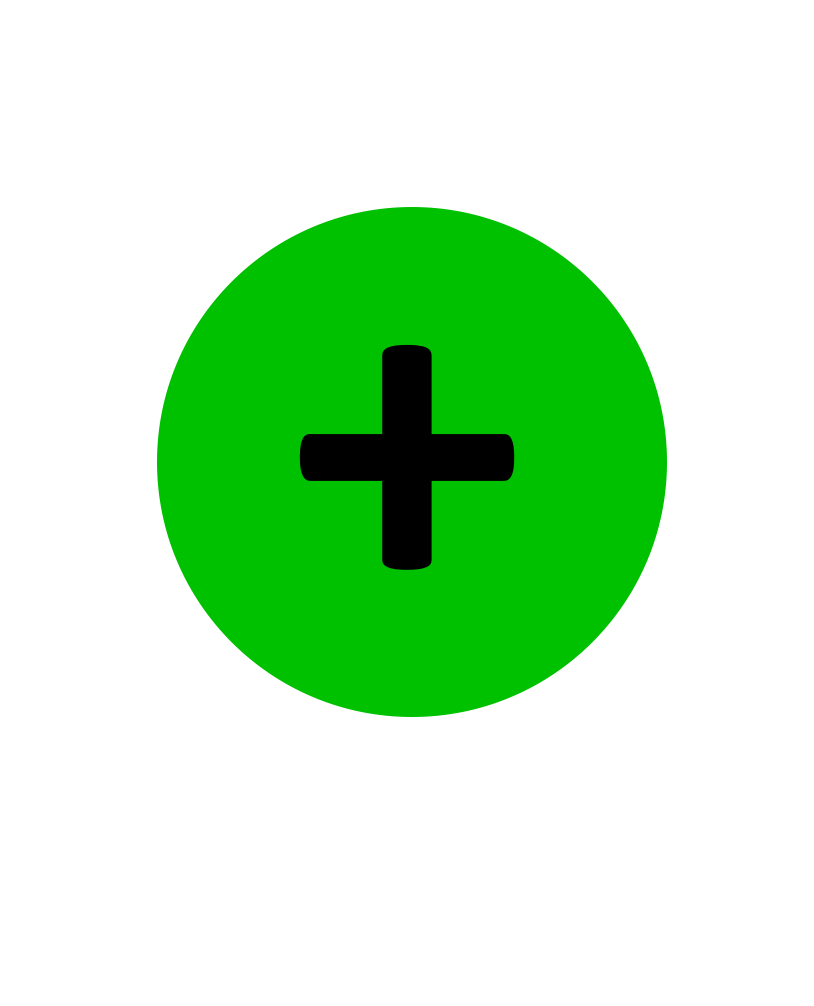 | 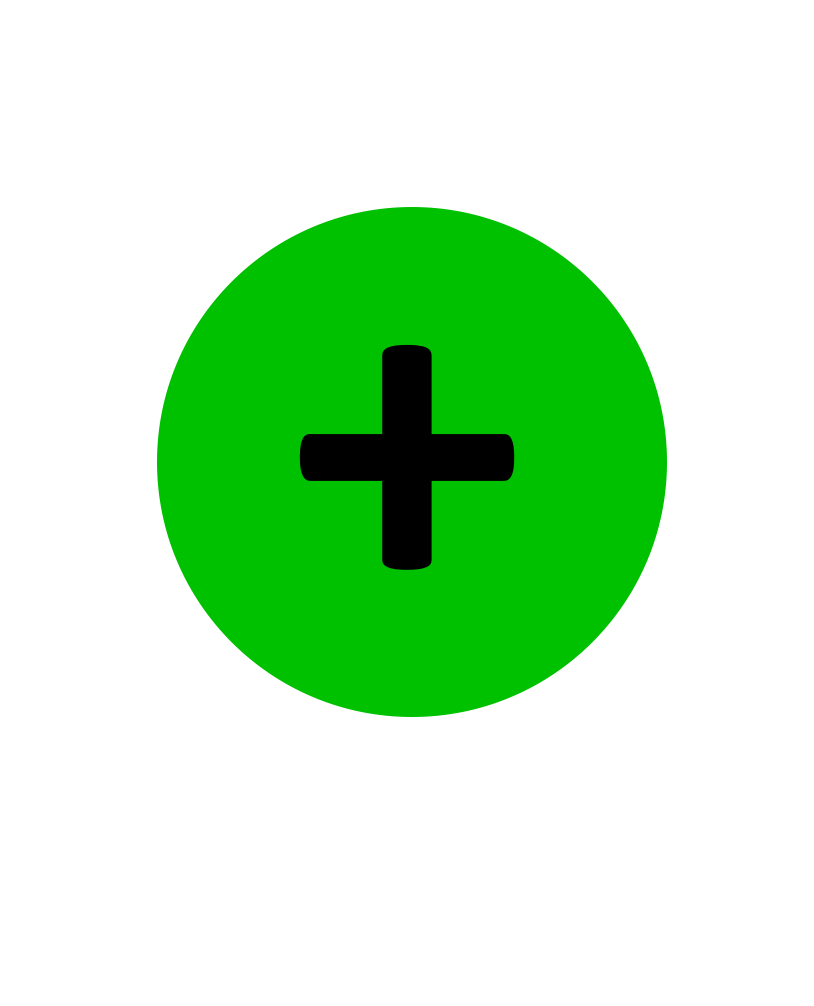 | 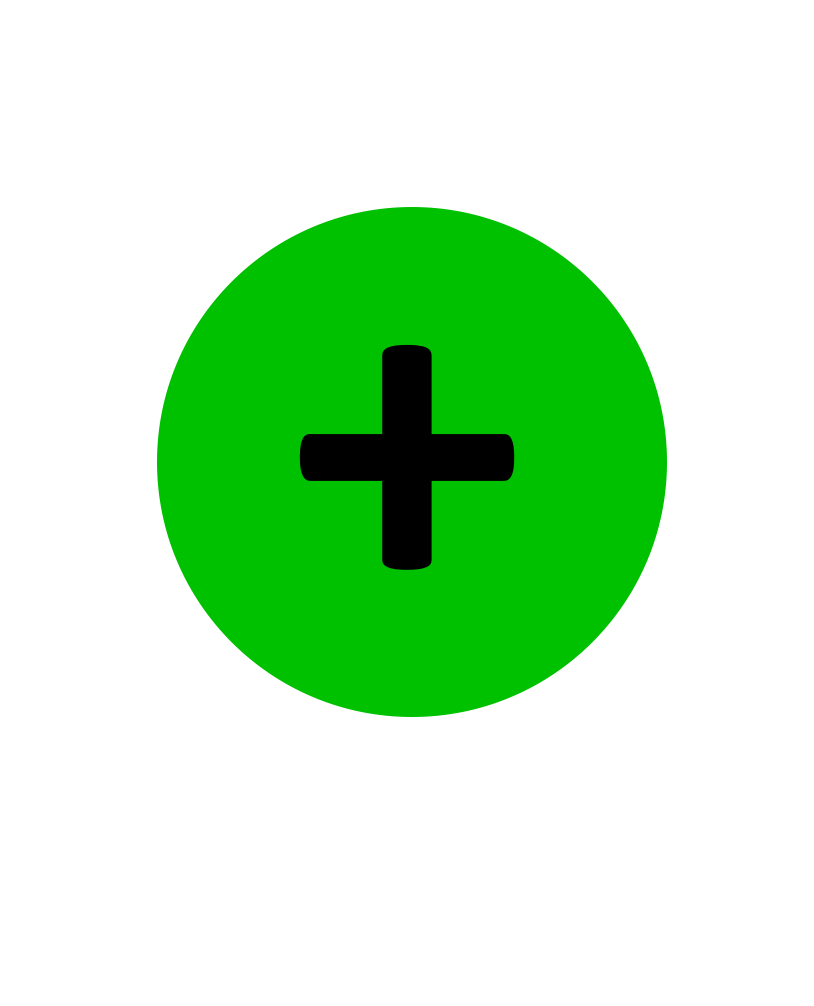 |
| **Kobayashi, 2008** [12] | | 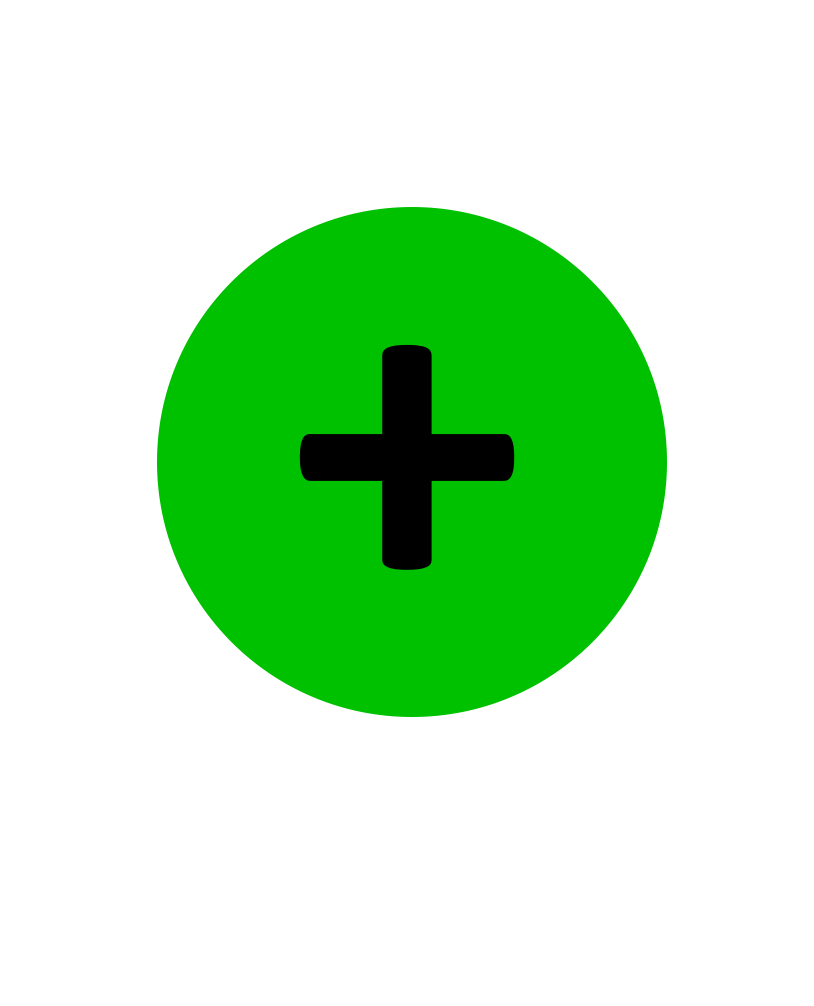 | 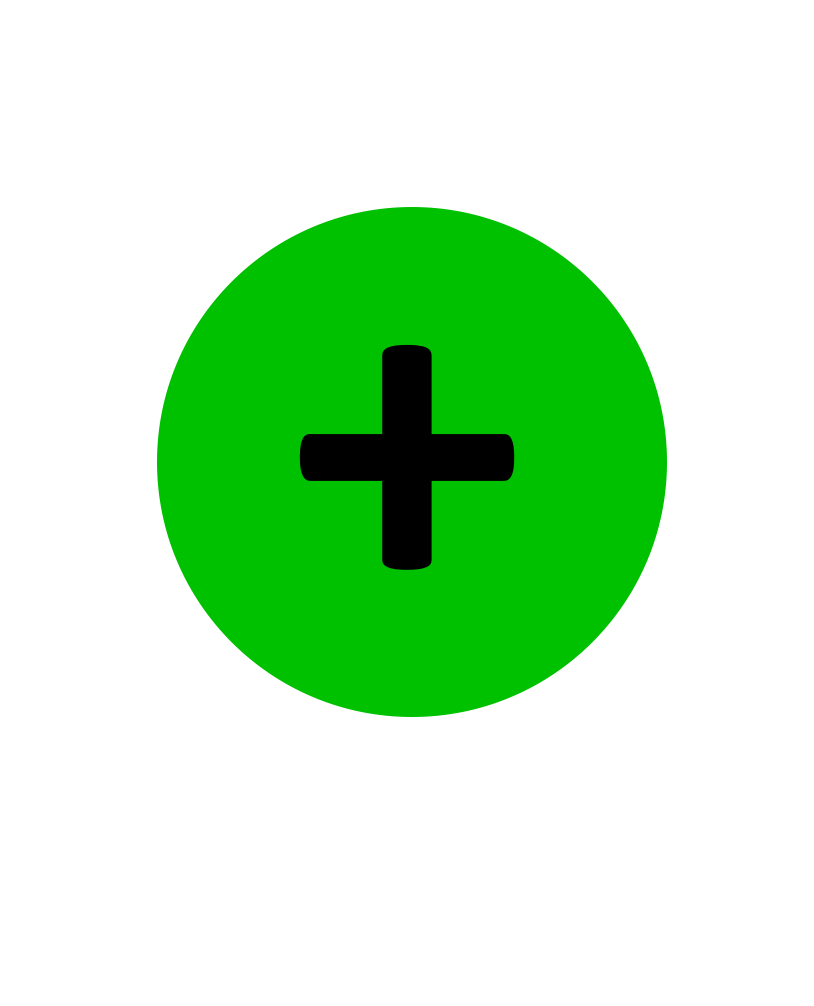 | 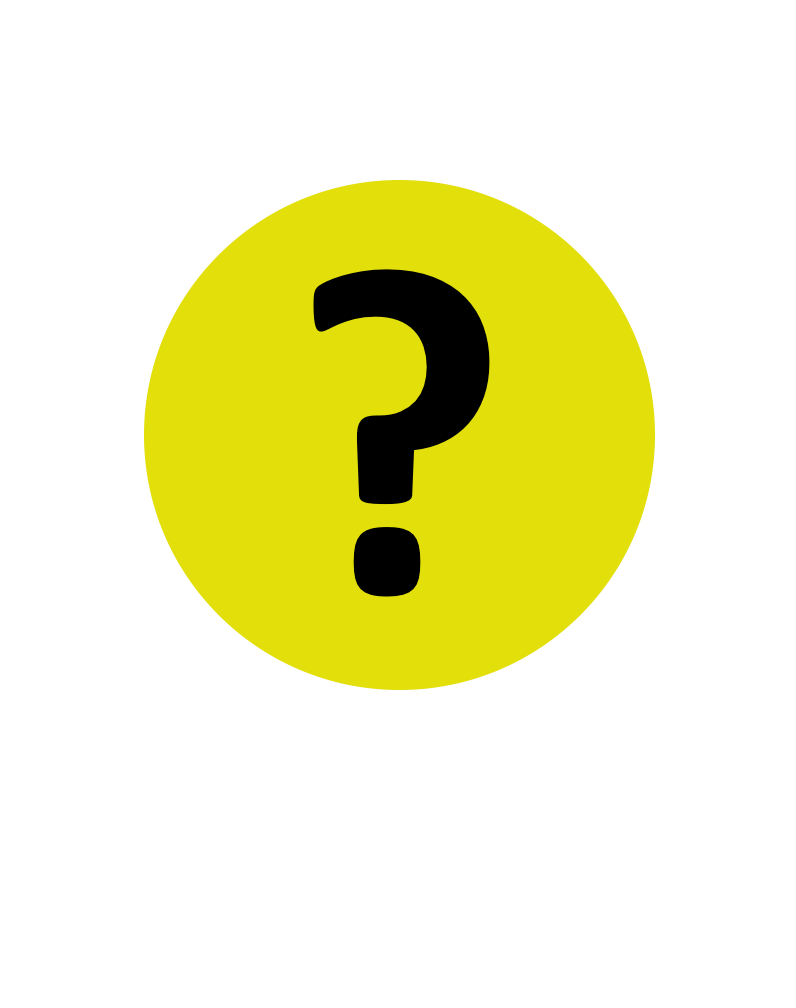 | 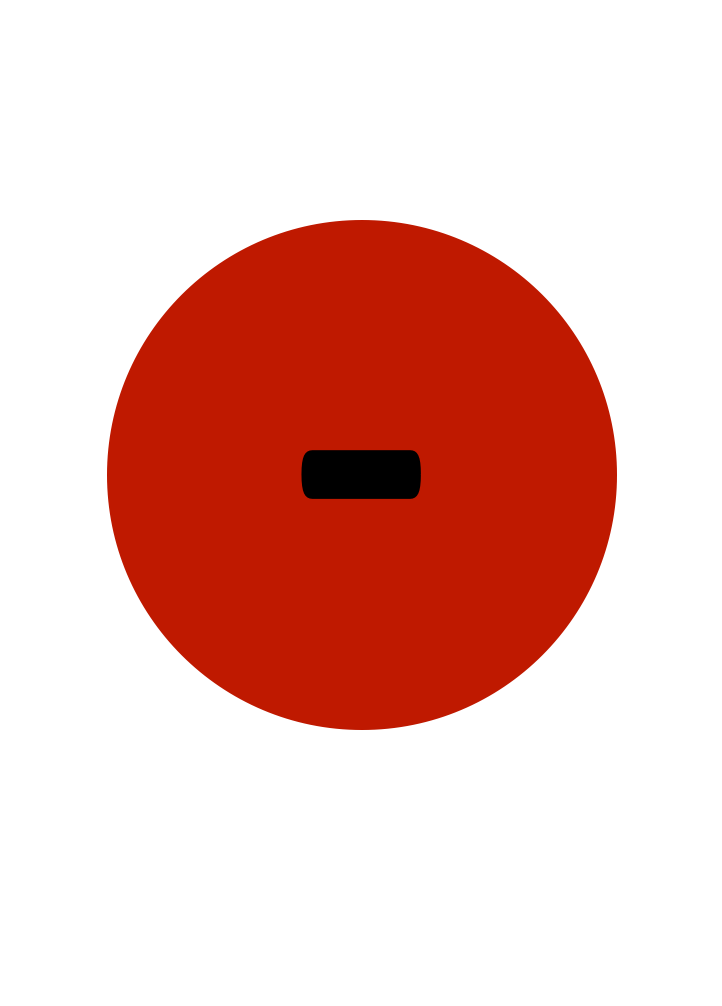 | 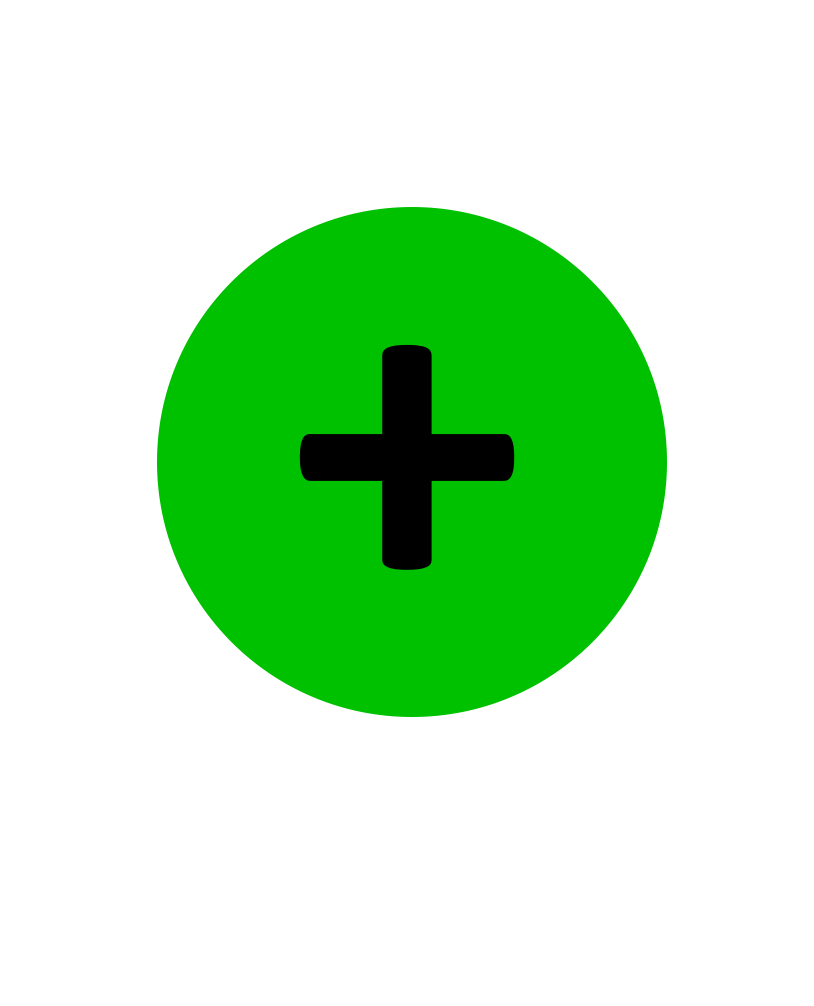 | 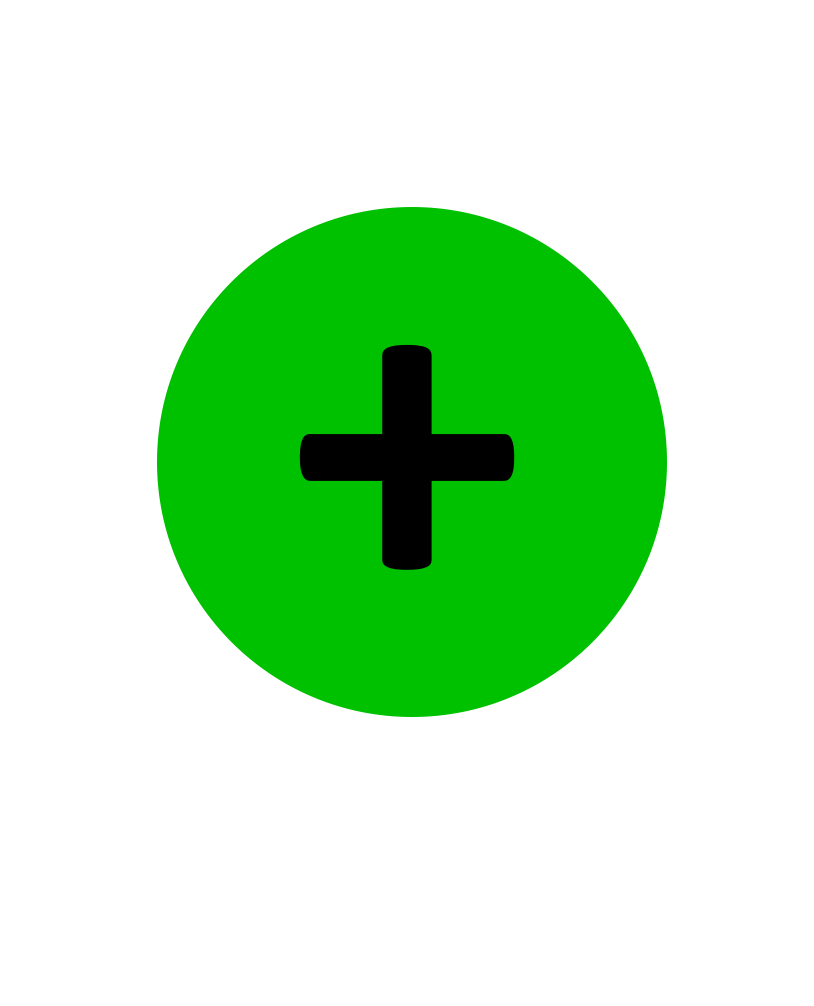 | 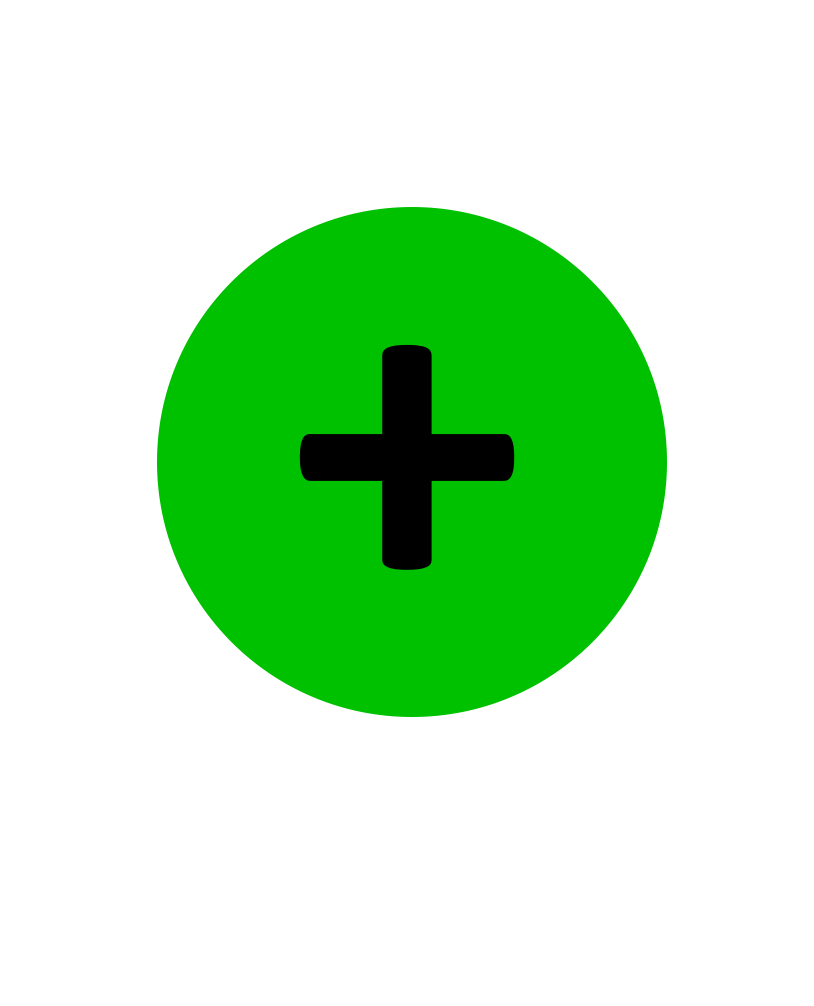 |
| **Yung, 2008** [13] | | 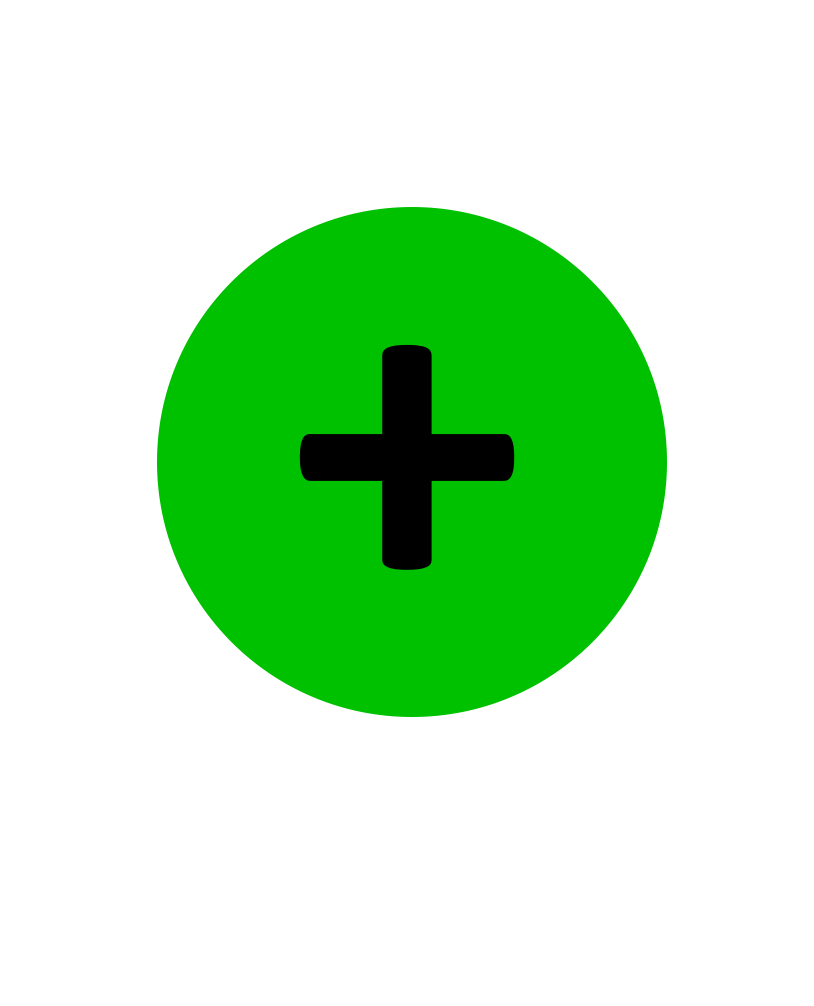 | 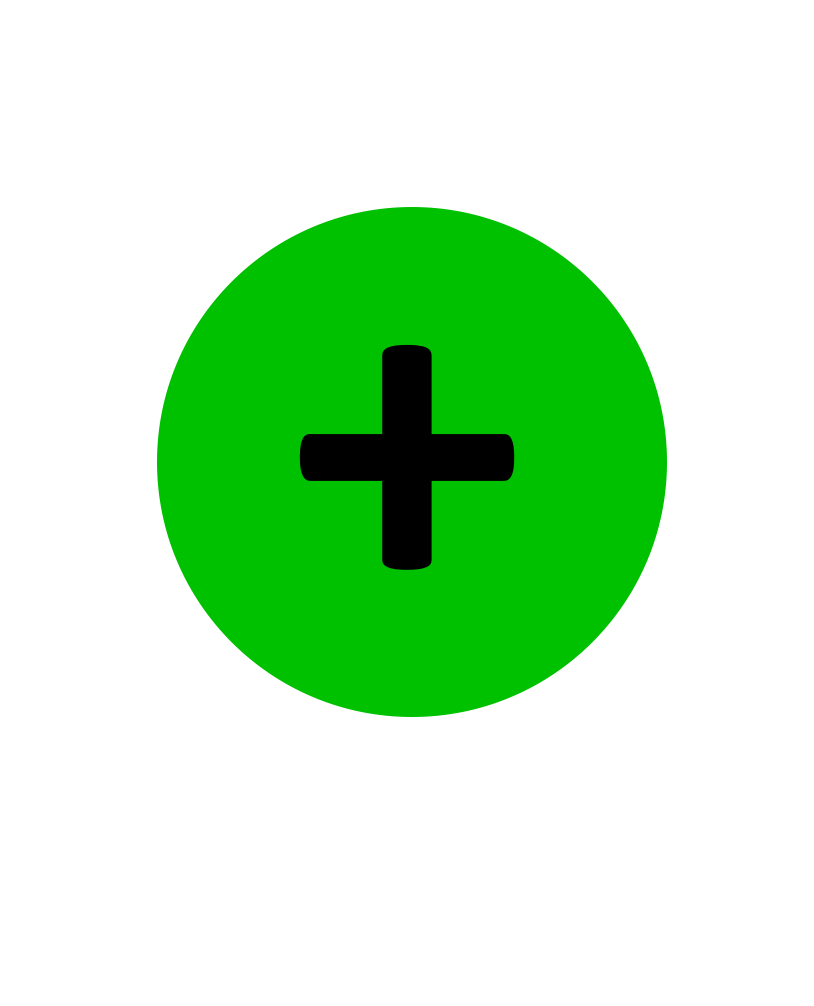 | 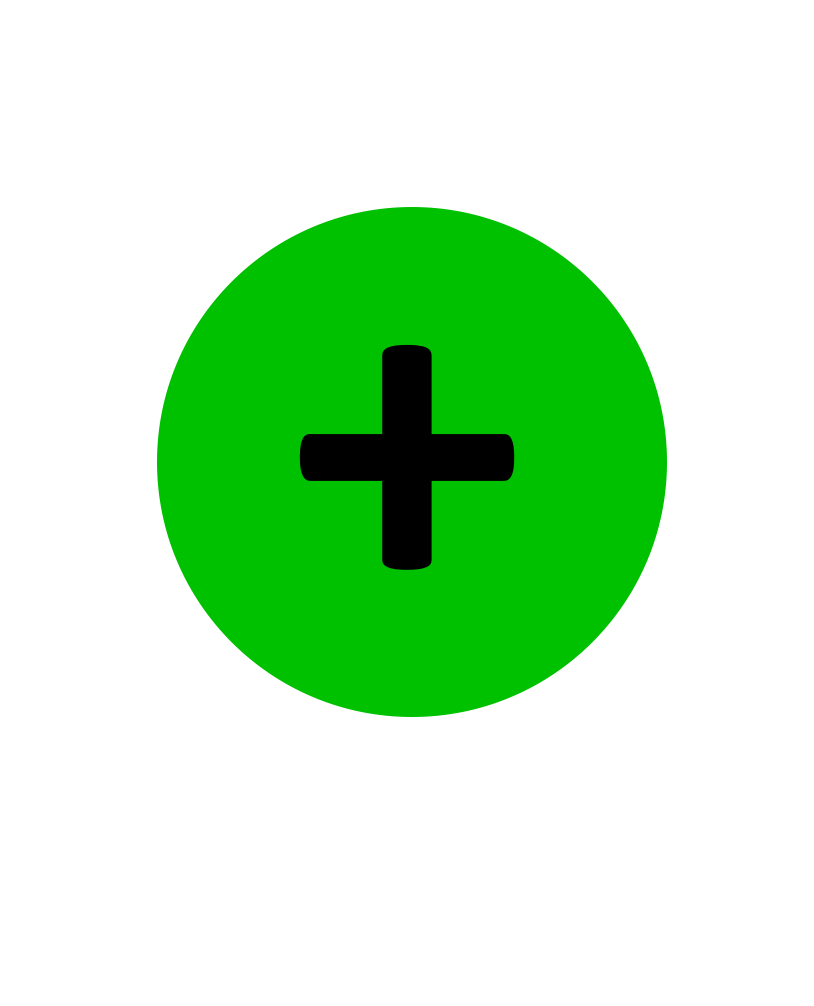 | 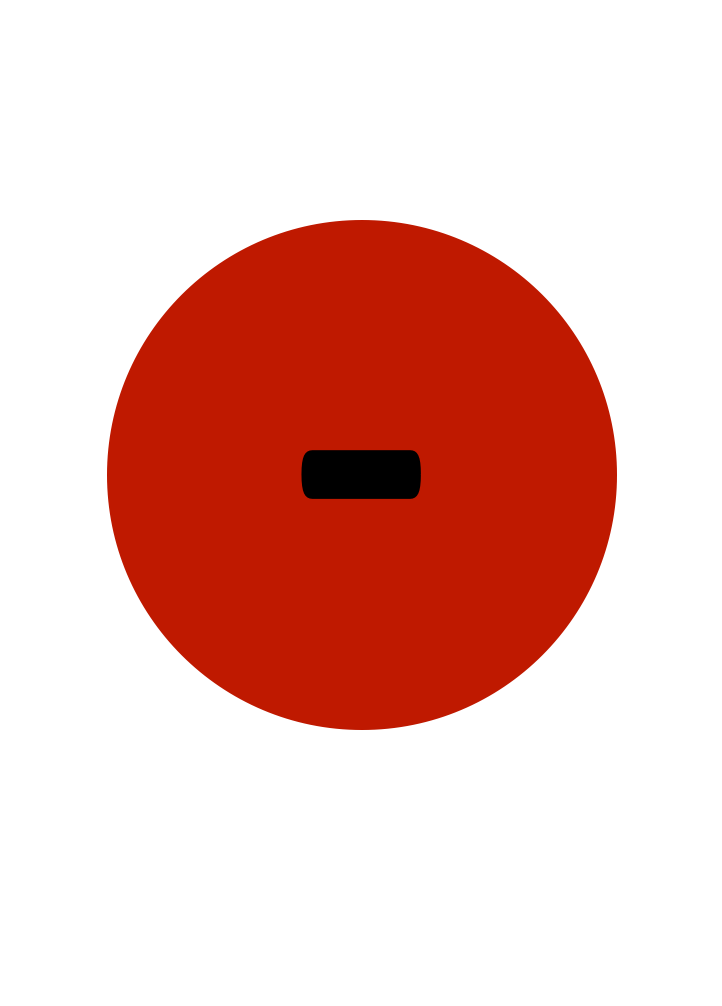 | 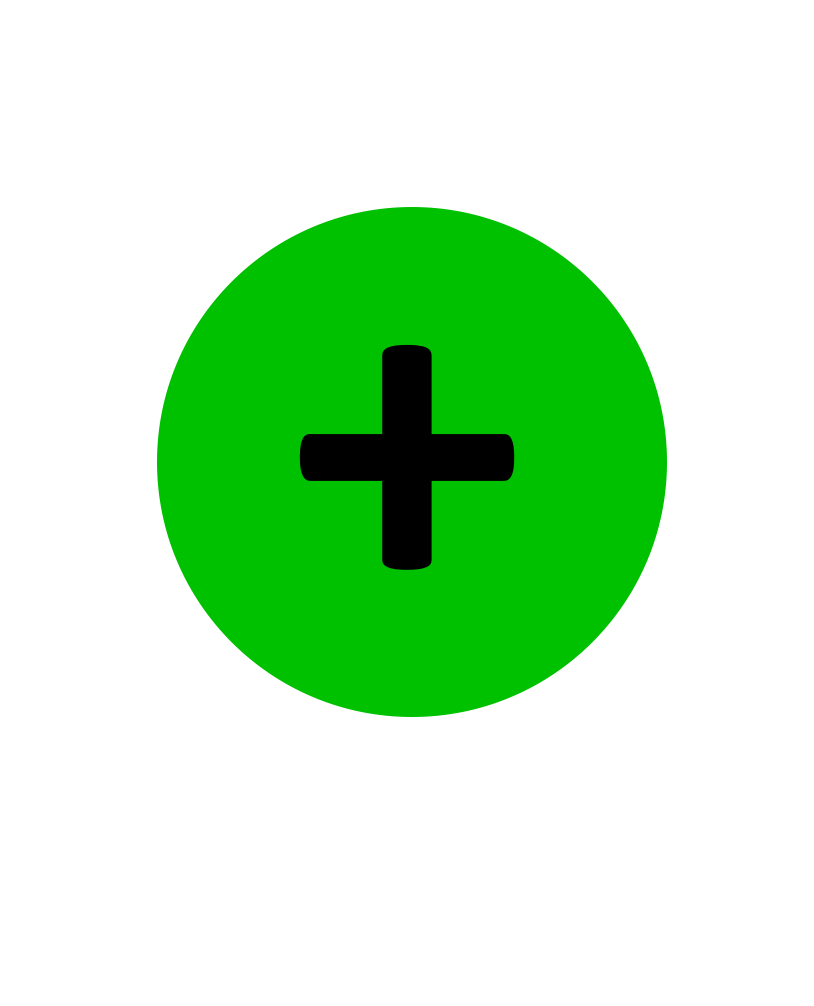 | 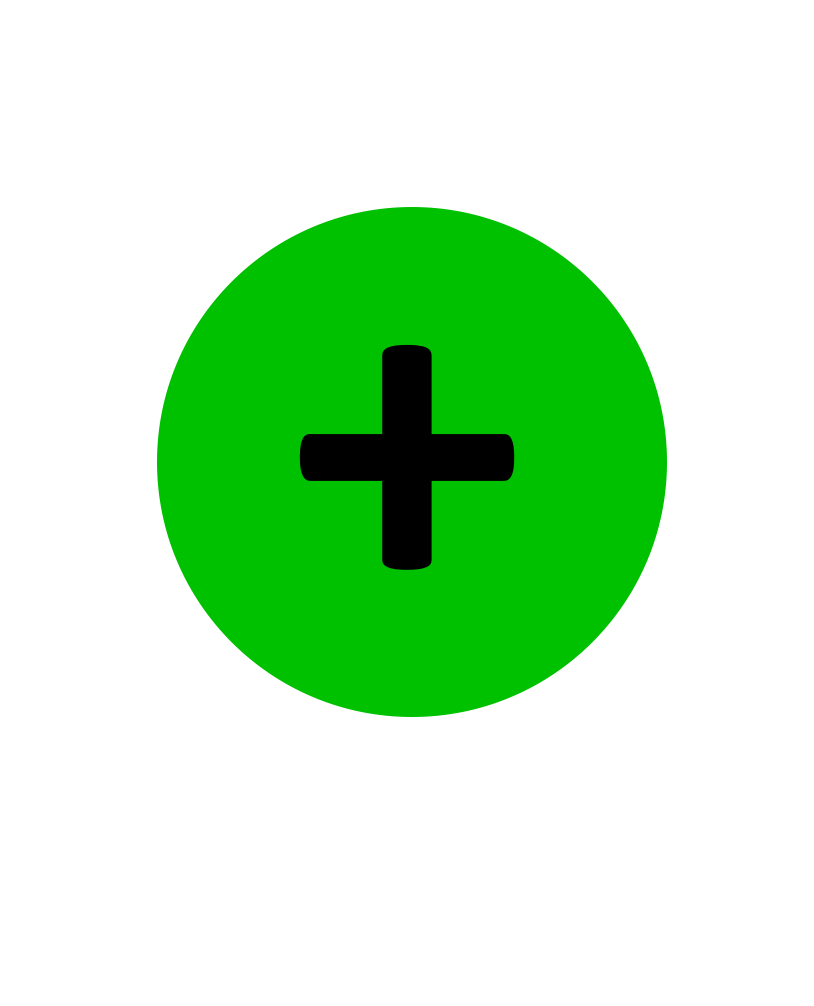 | 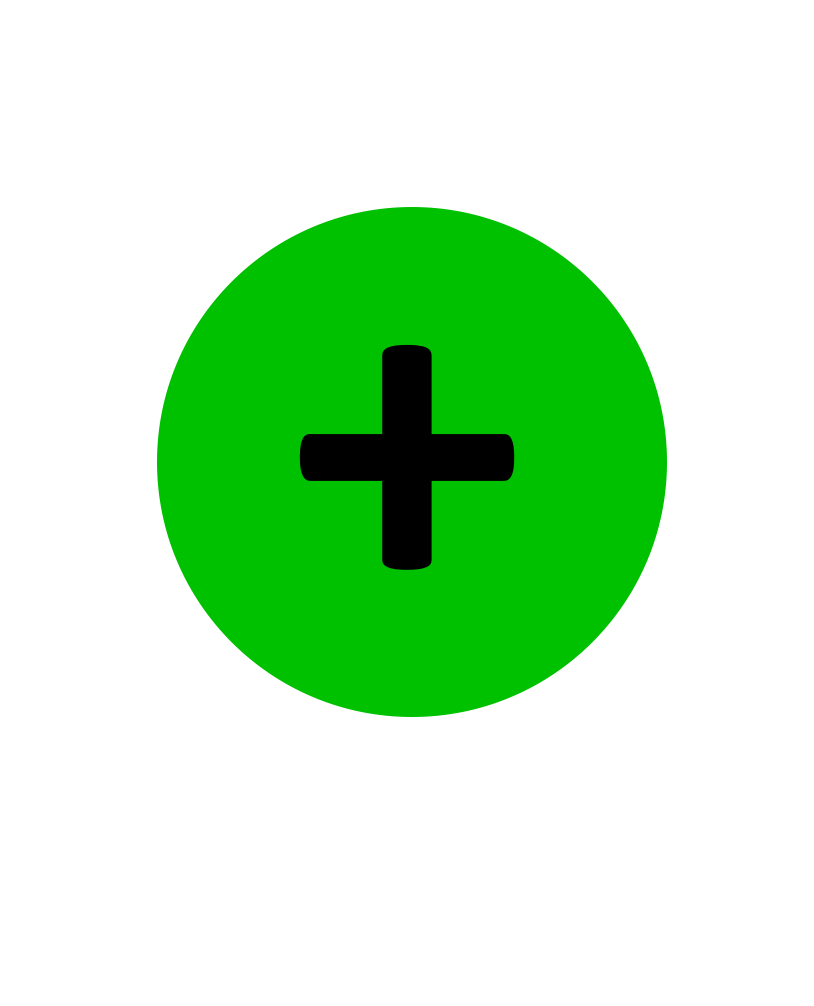 |
| **Woods, 2009** [14] | | 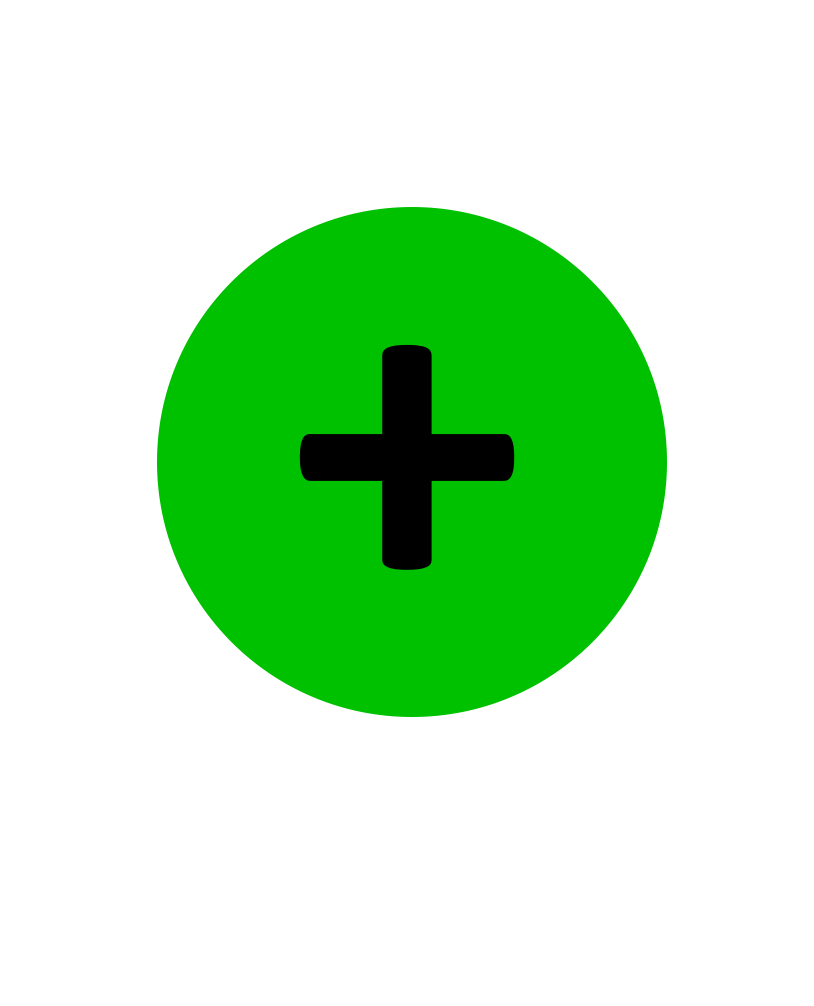 | 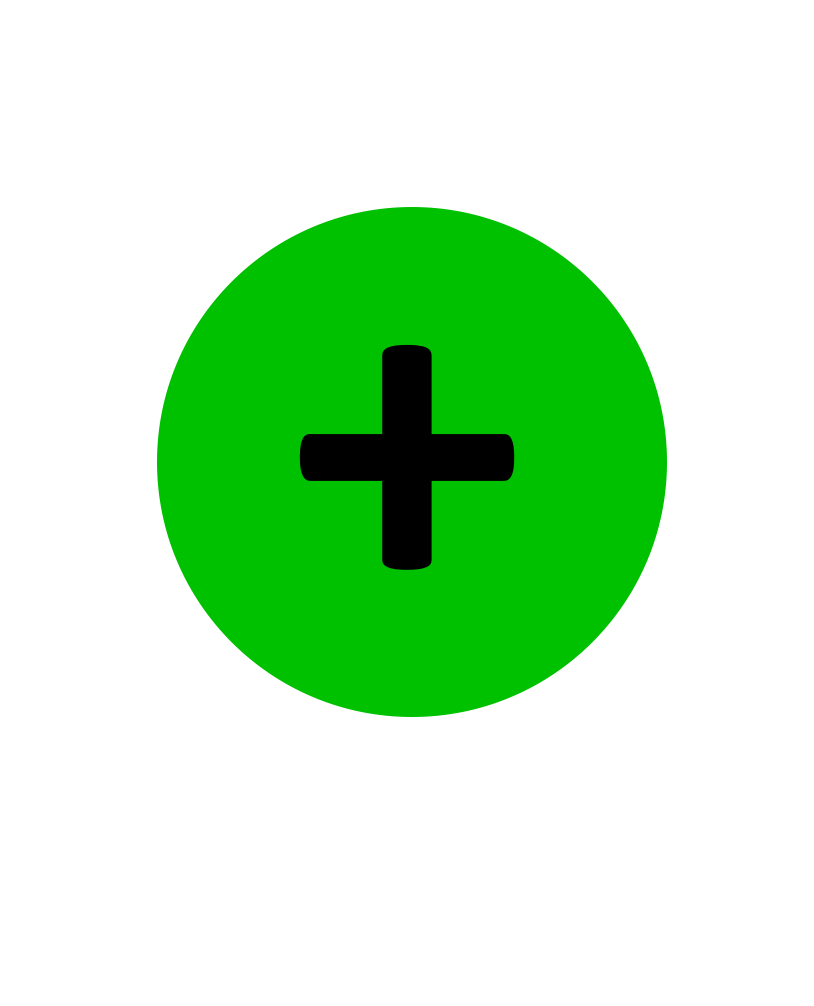 | 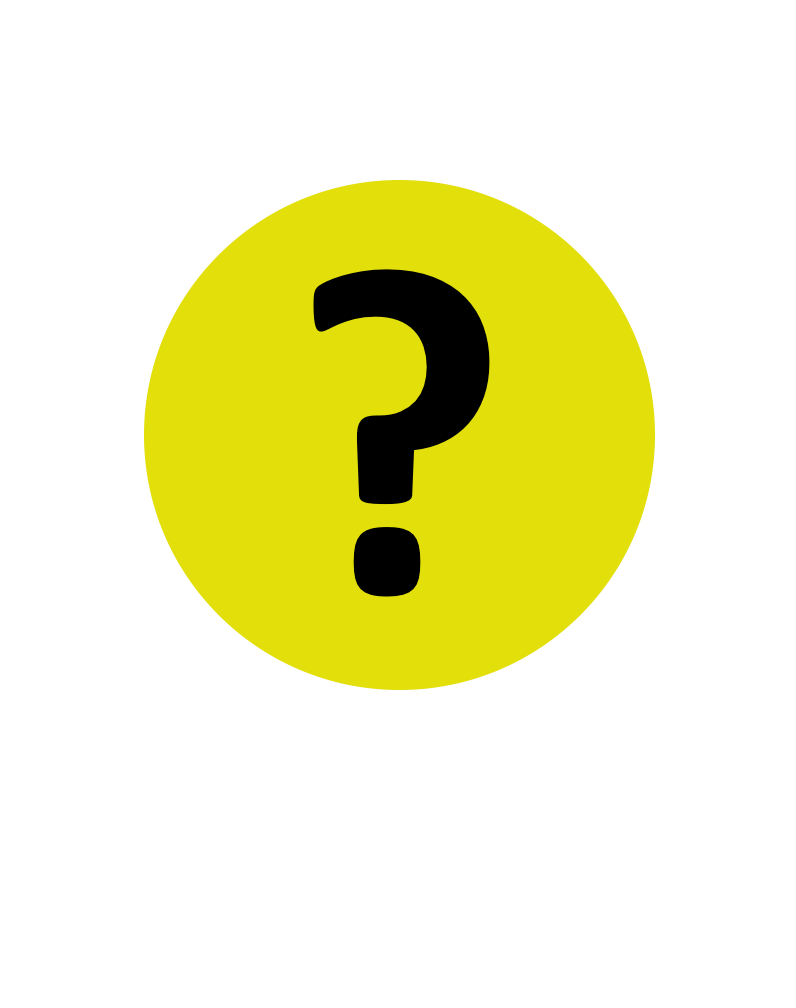 | 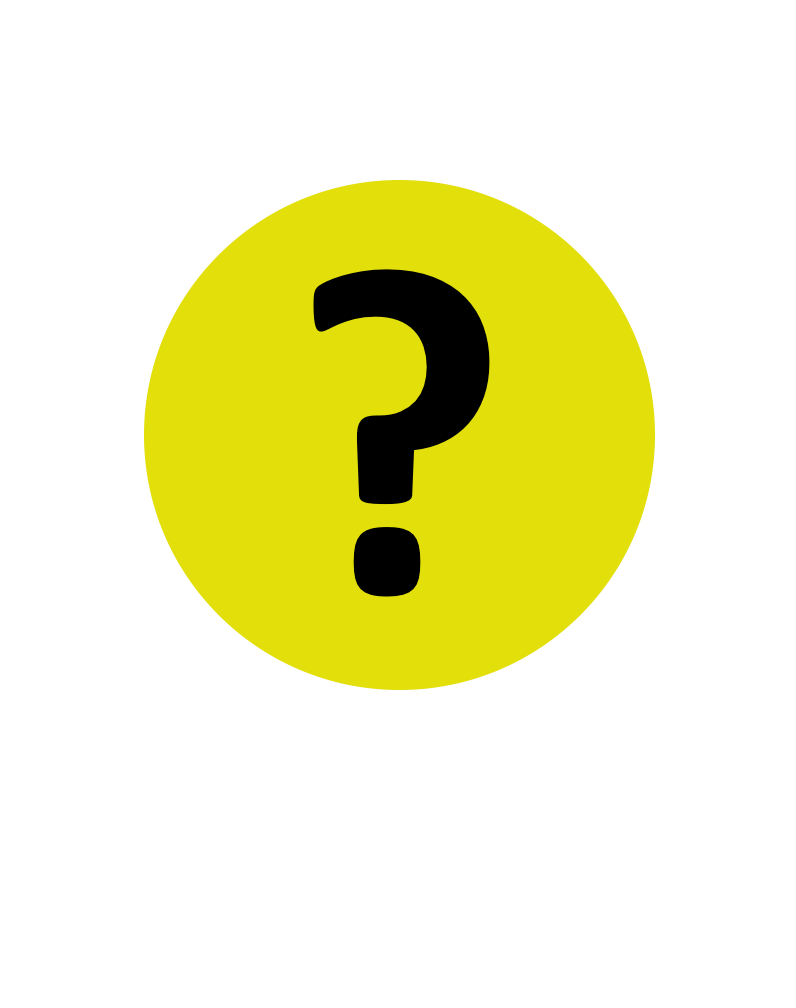 | 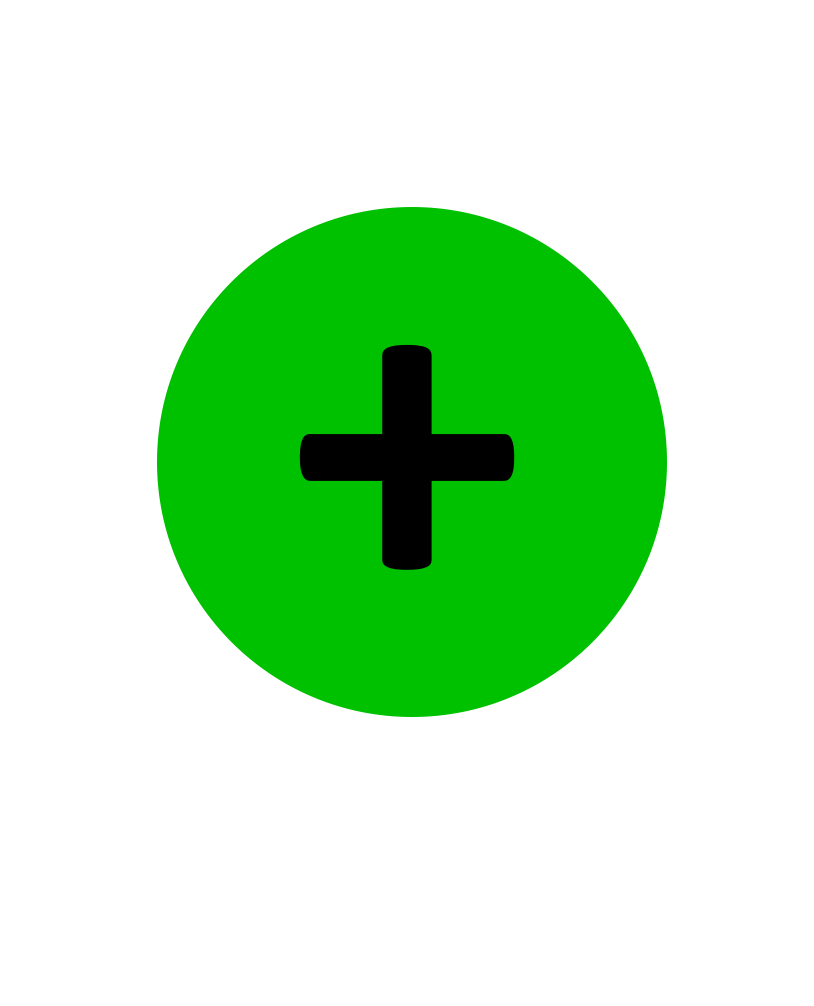 | 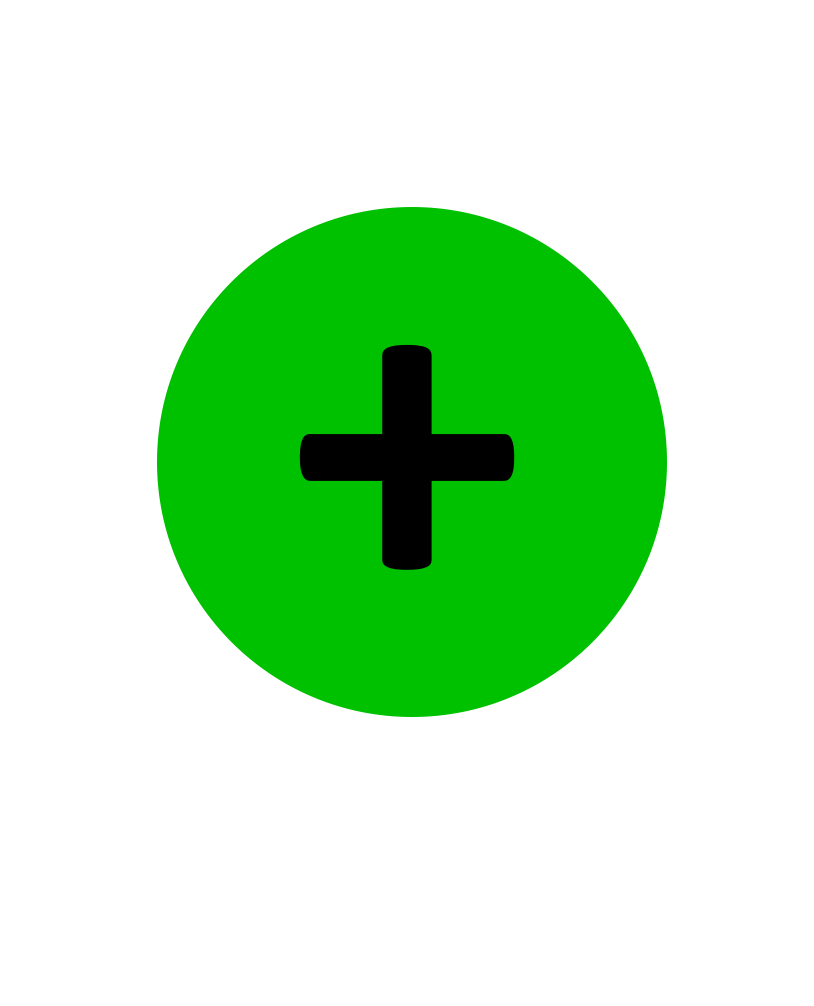 | 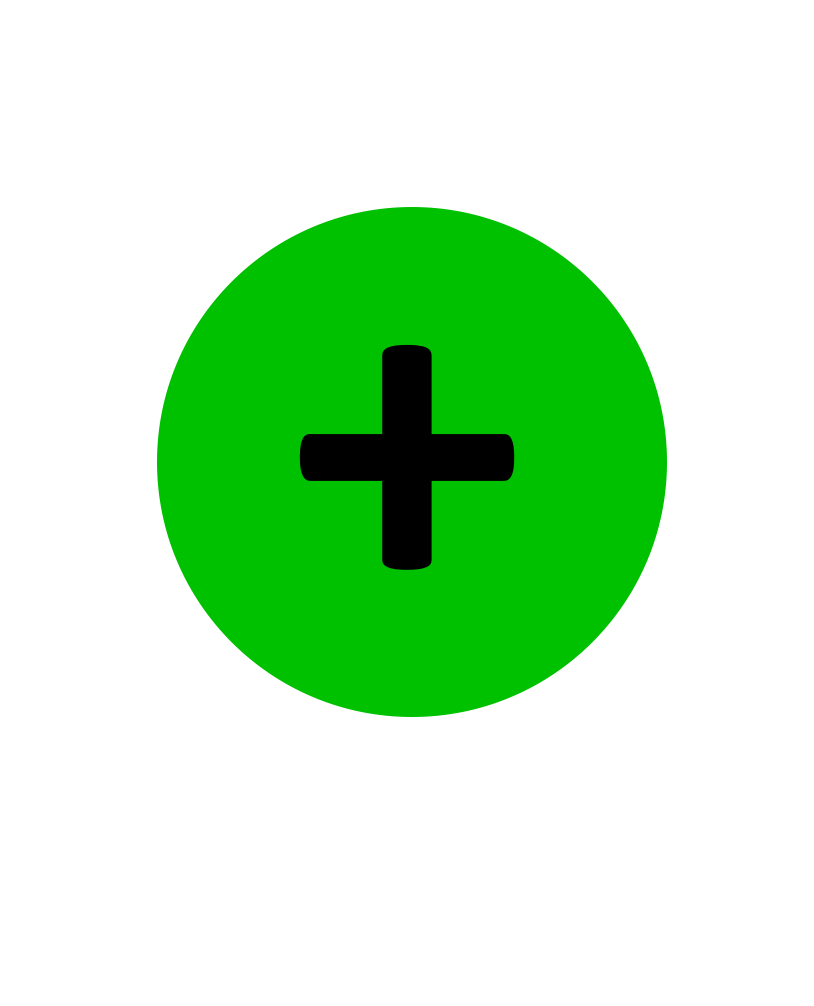 |
| **Liu, 2011** [15] | | 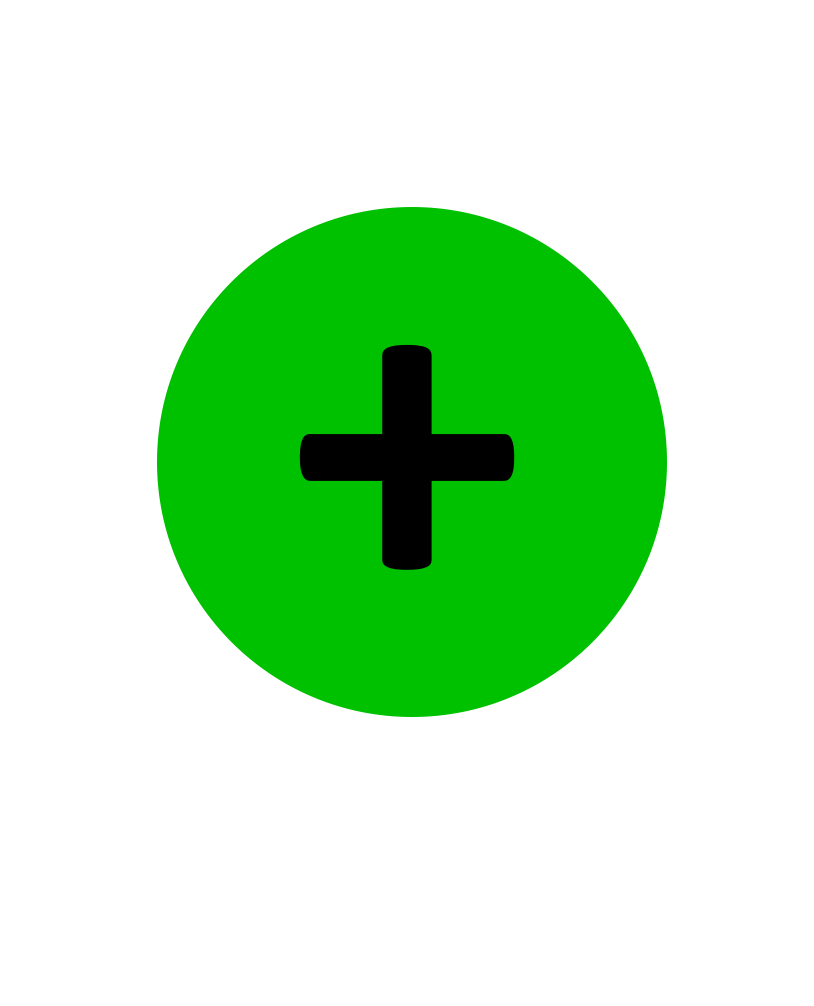 | 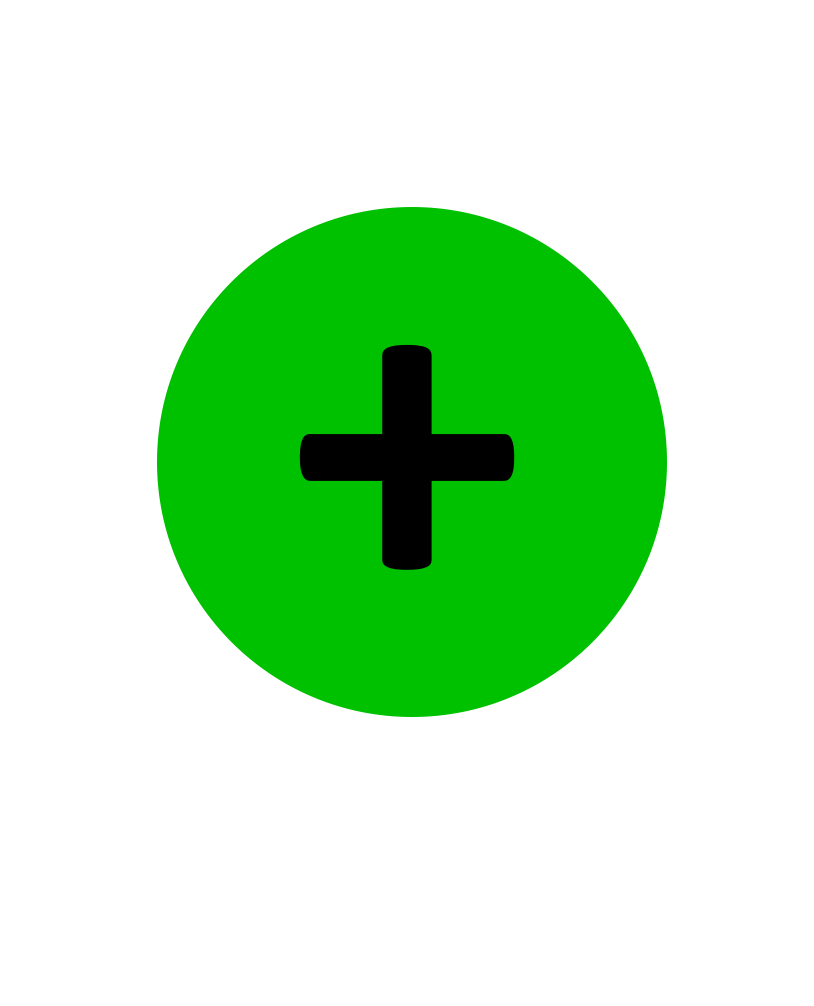 | 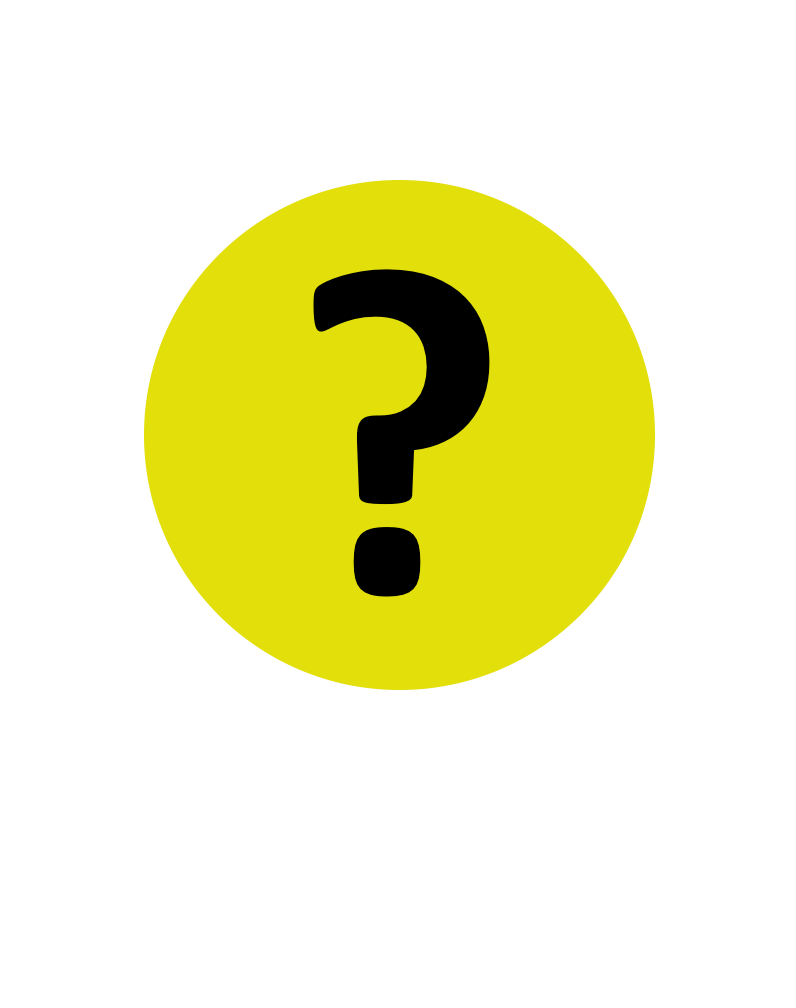 | 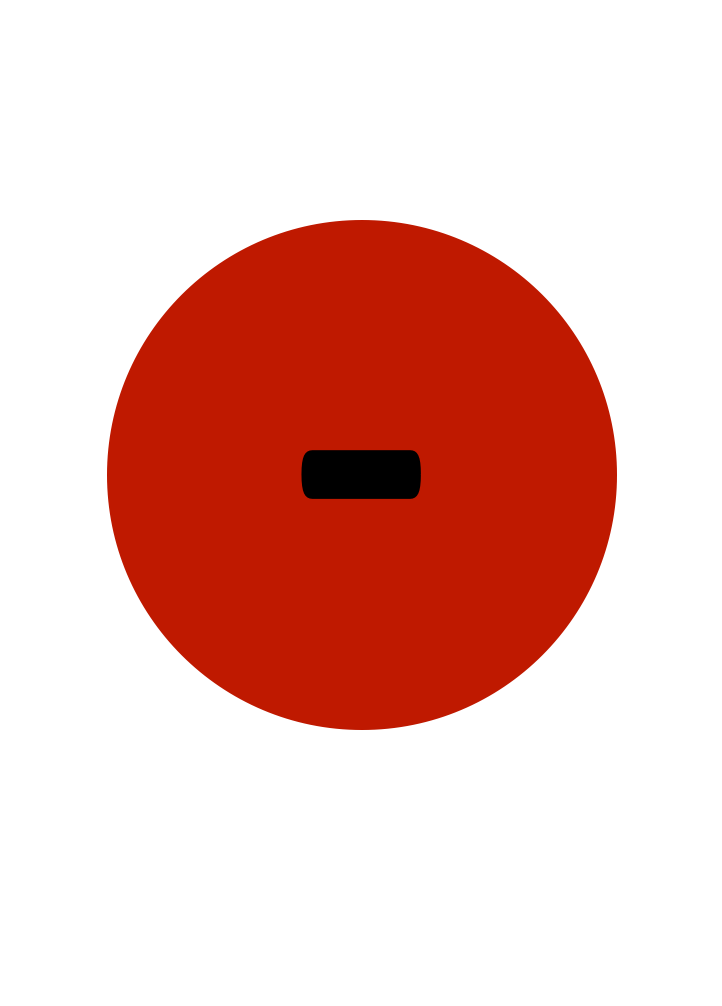 | 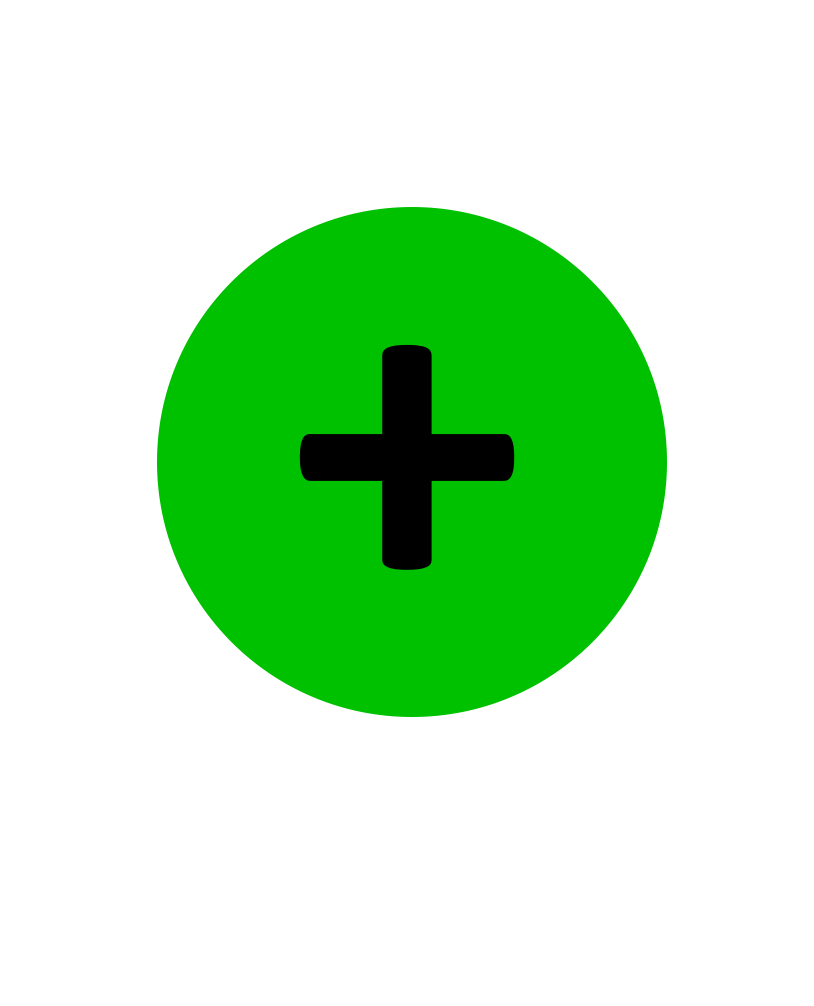 | 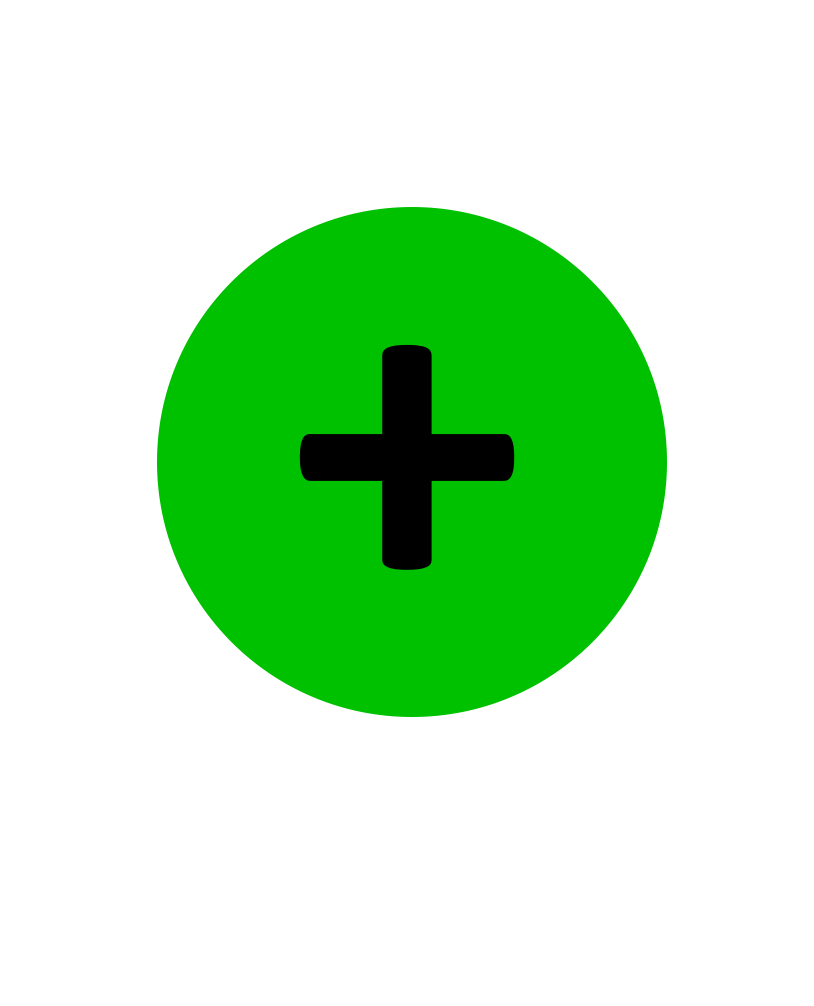 | 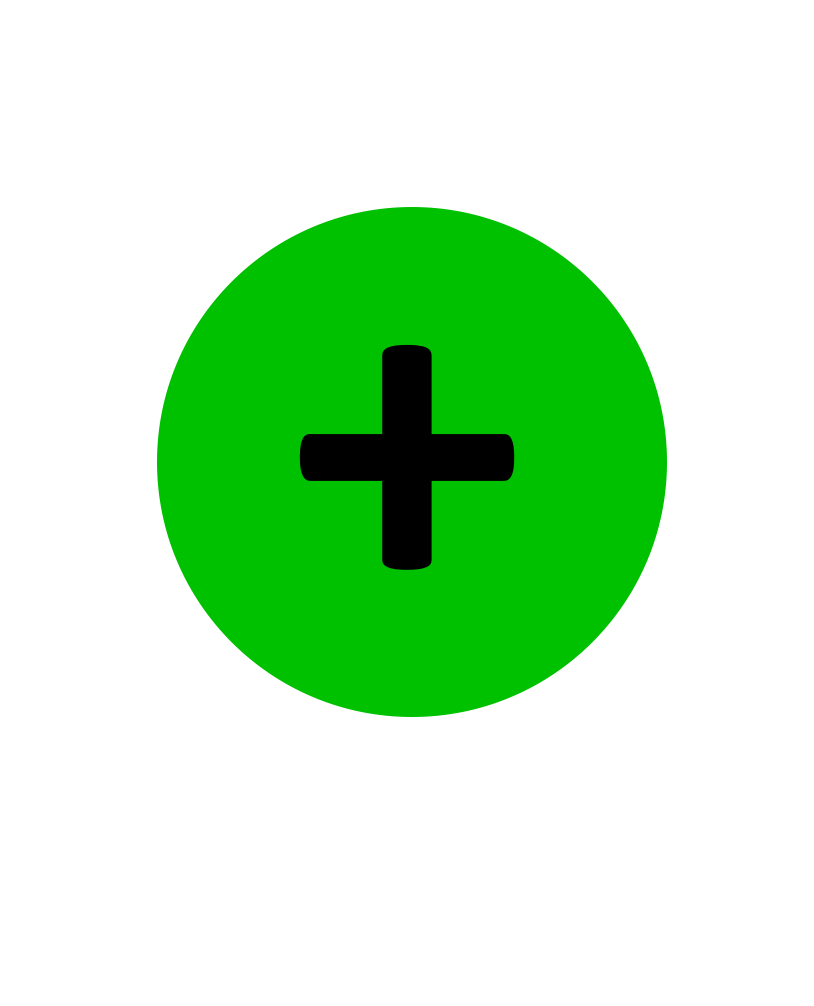 |
| **Addington, 2012** [16] | | 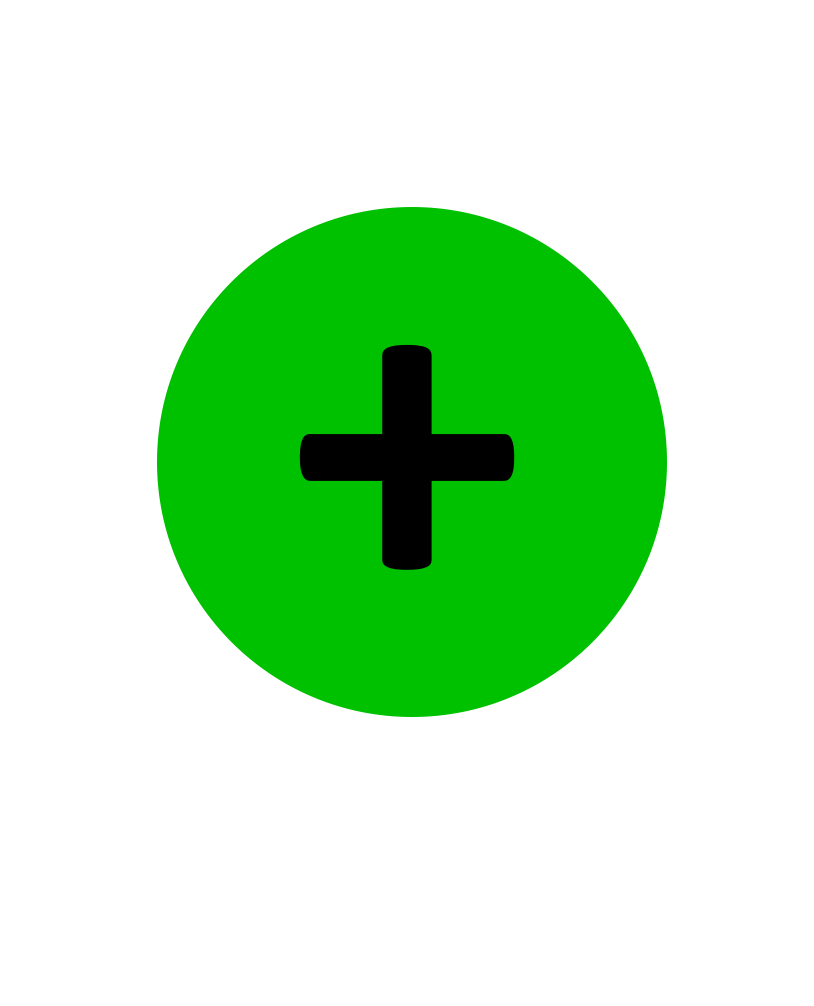 | 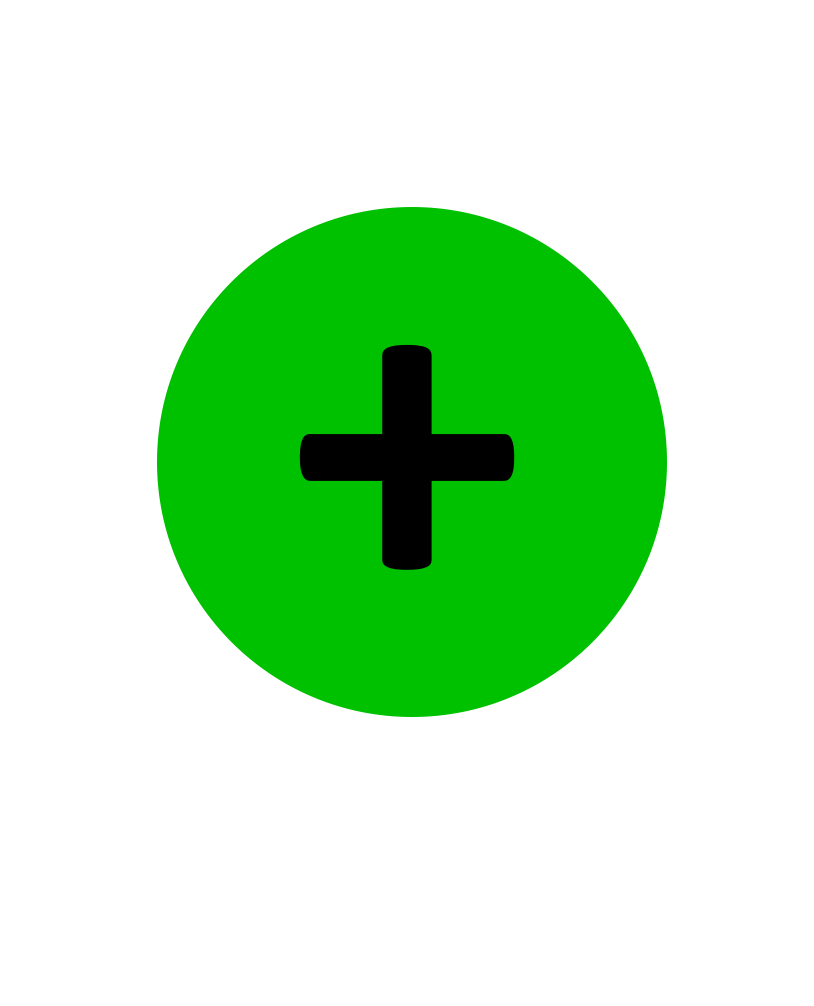 | 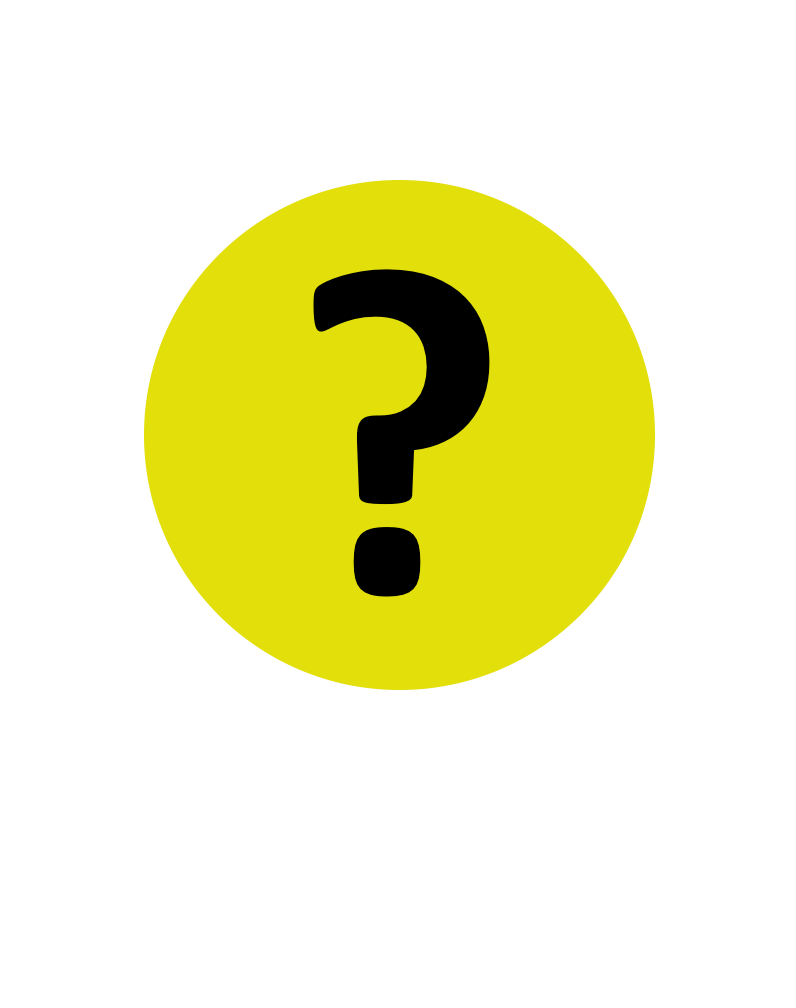 | 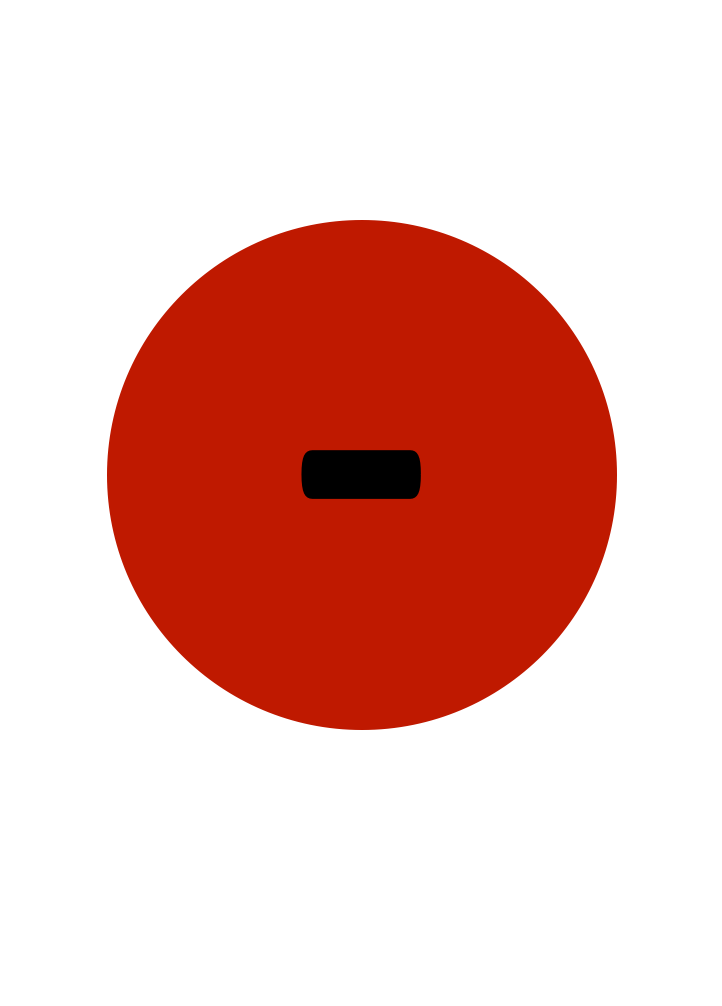 | 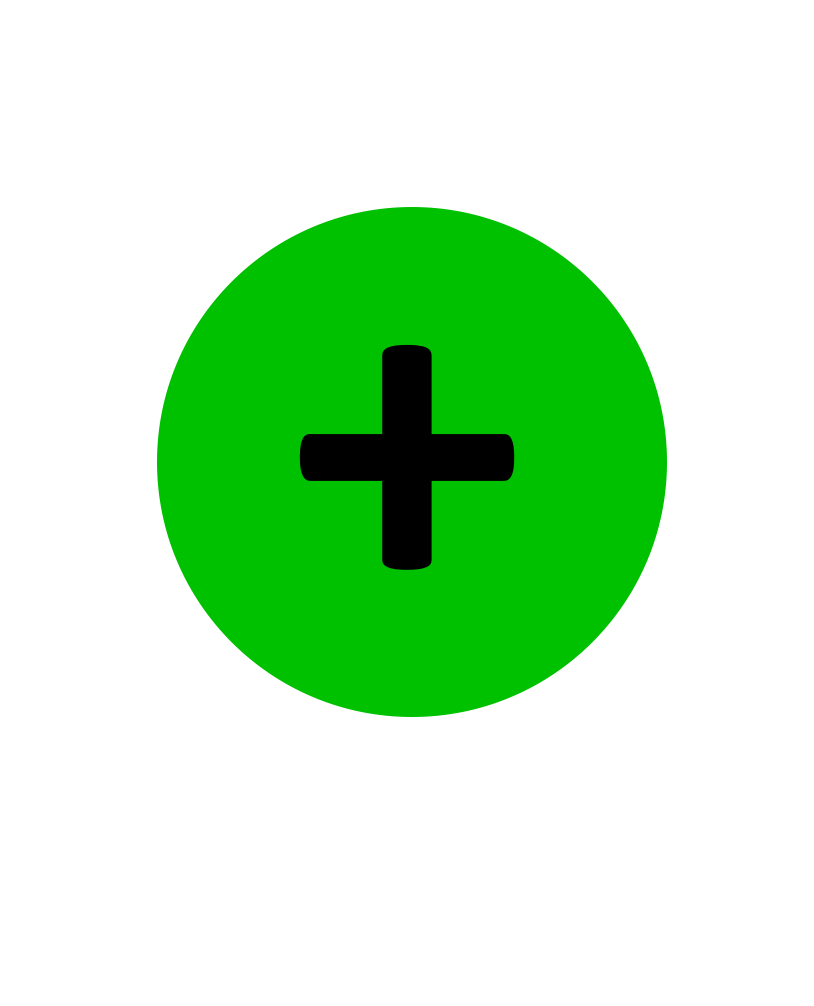 | 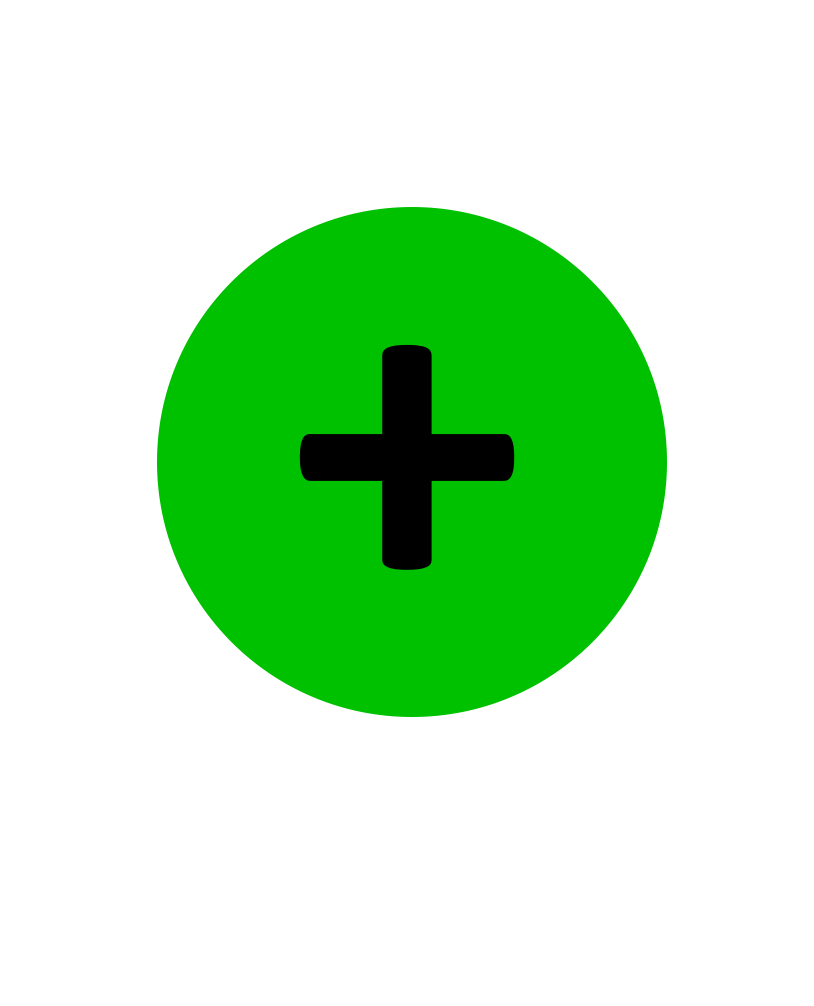 | 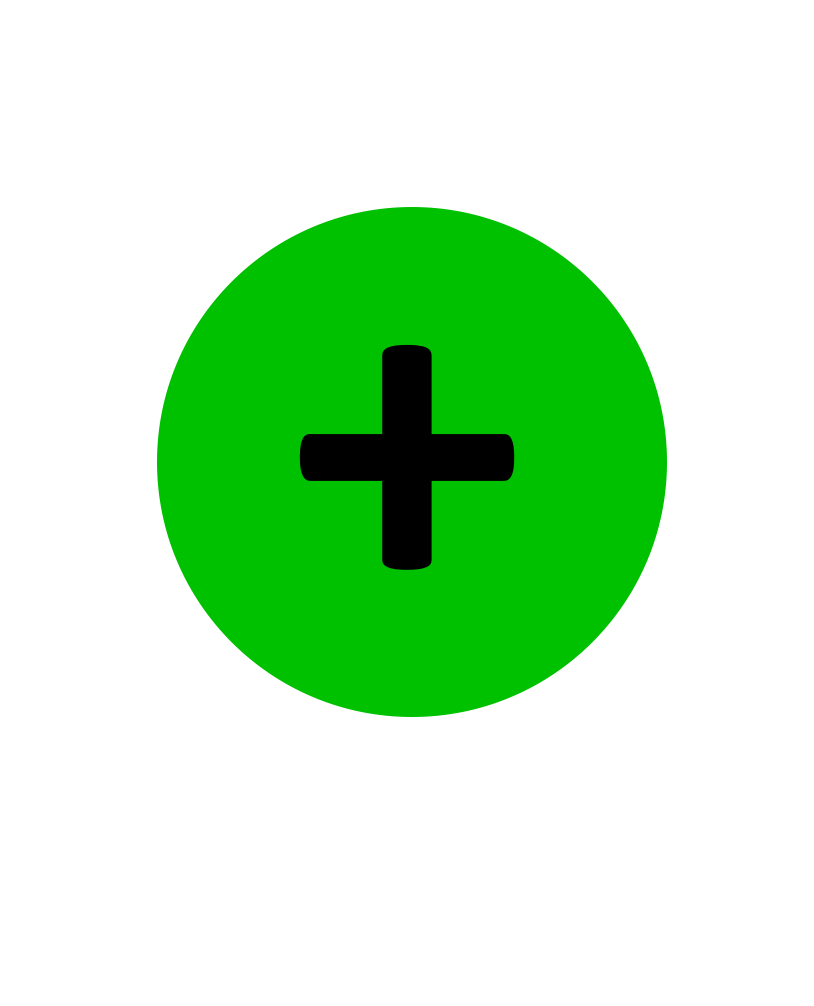 |
| **Simon, 2012** [17] | | 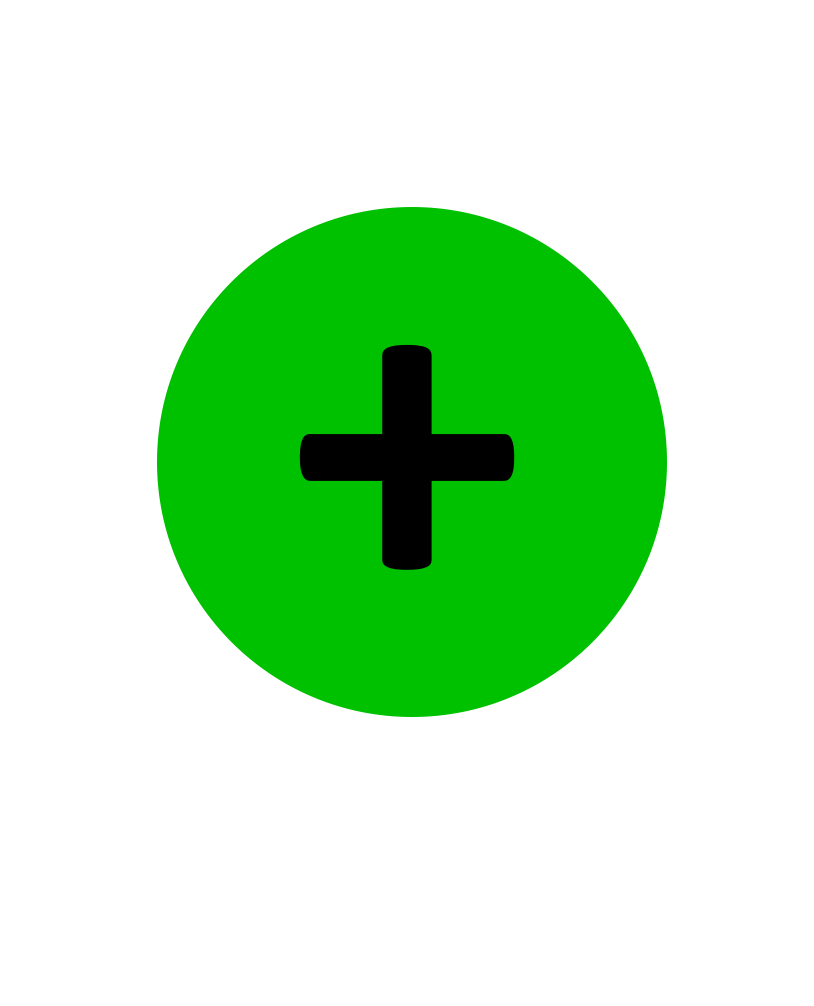 | 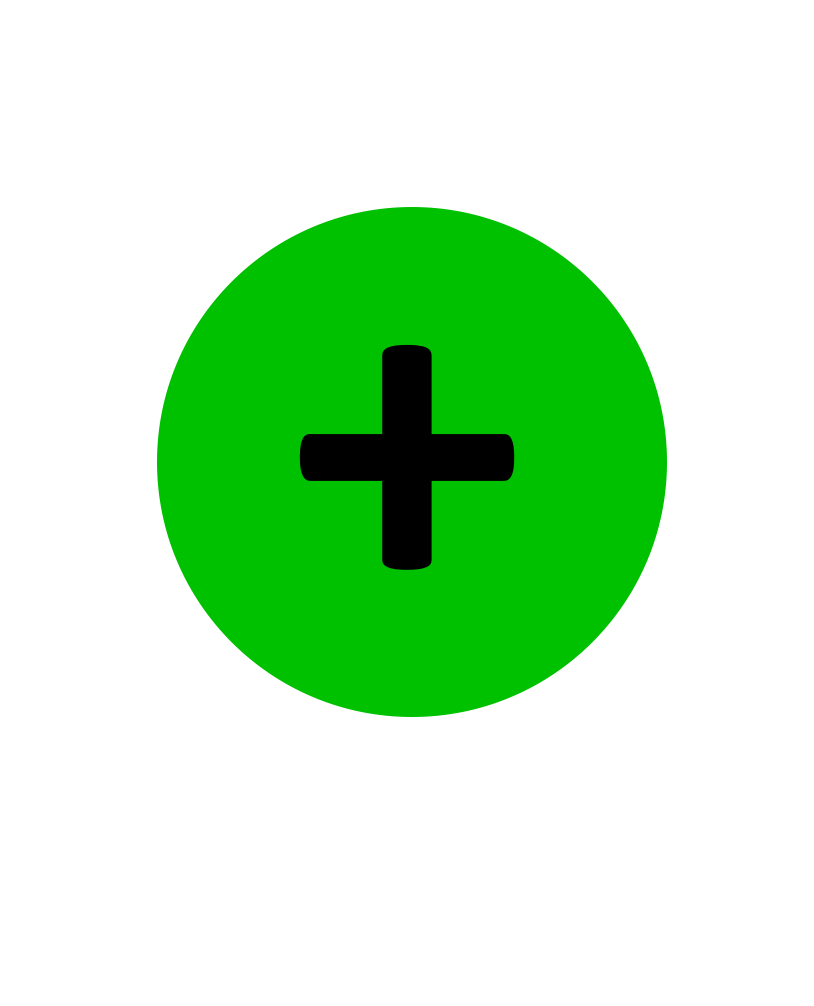 | 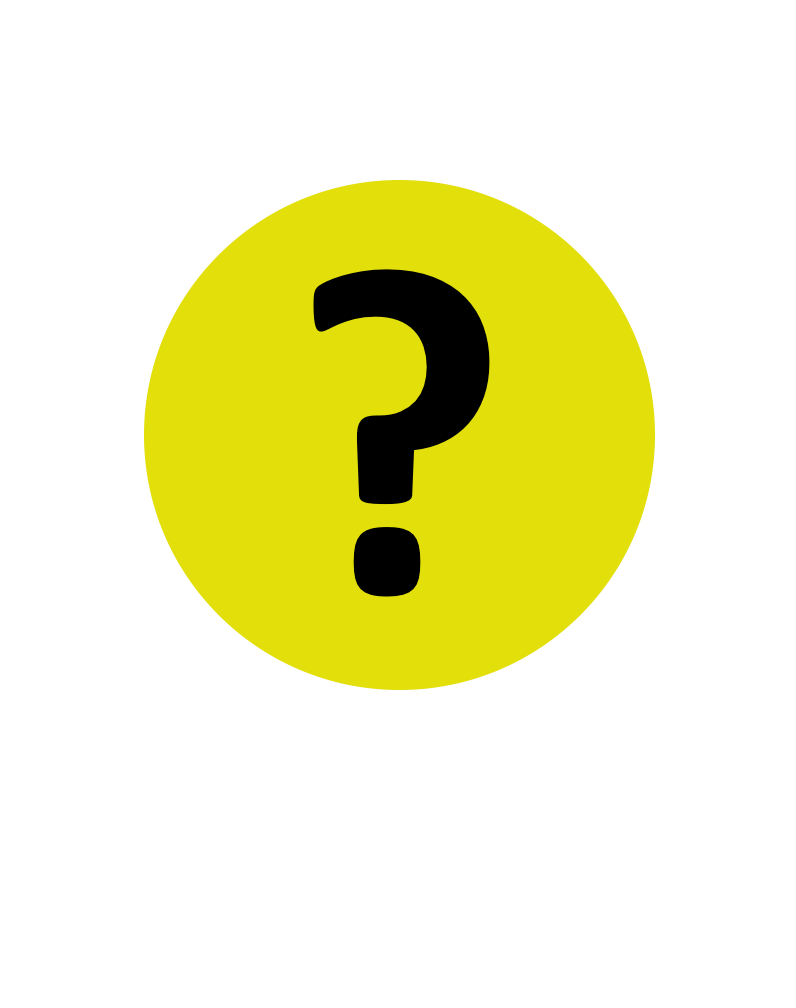 | 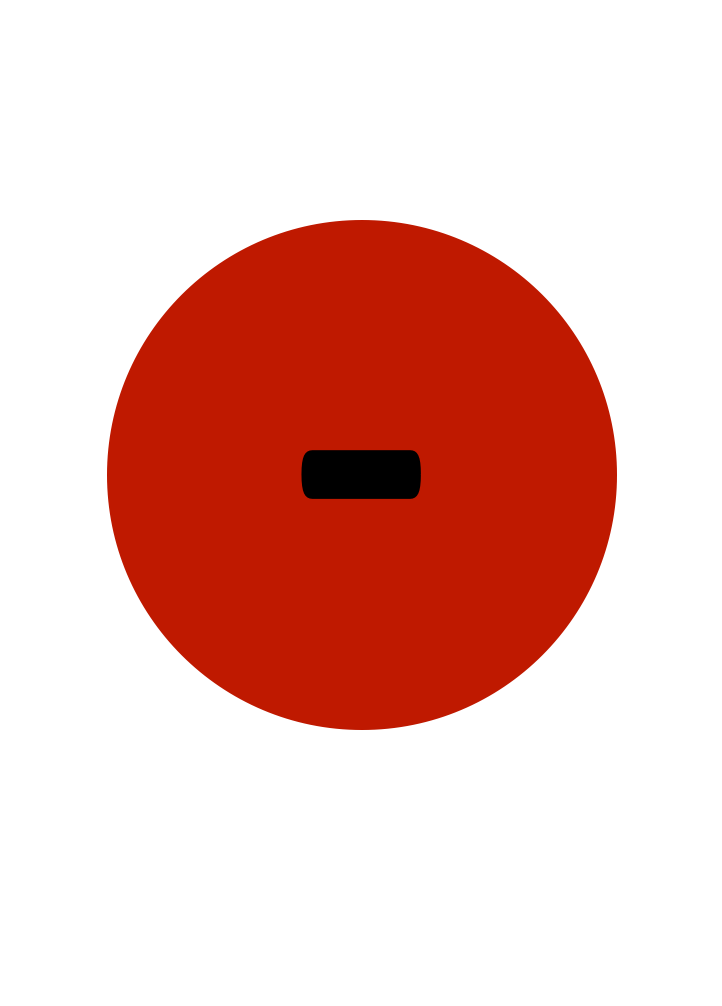 | 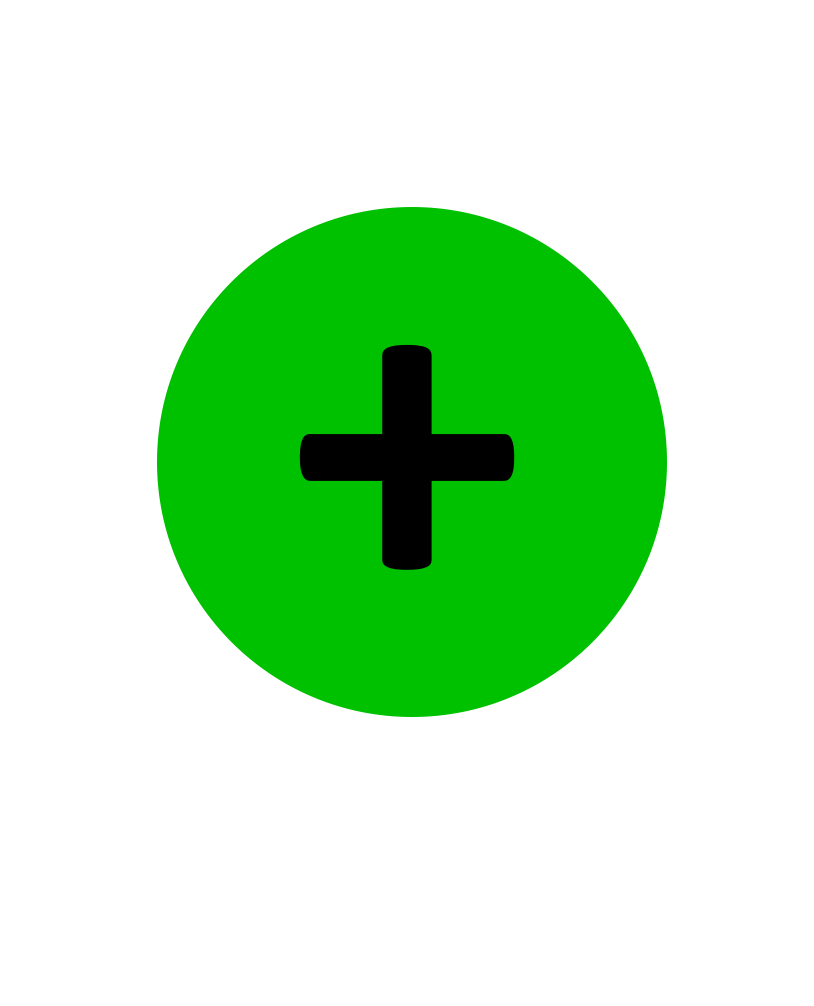 | 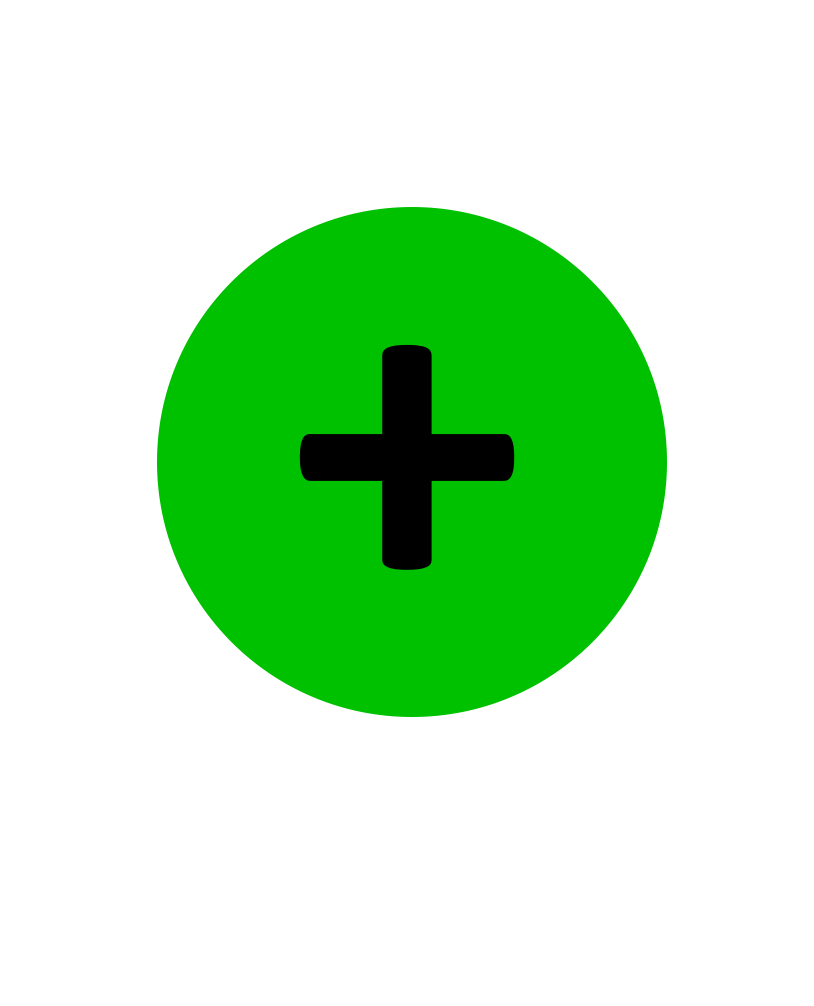 | 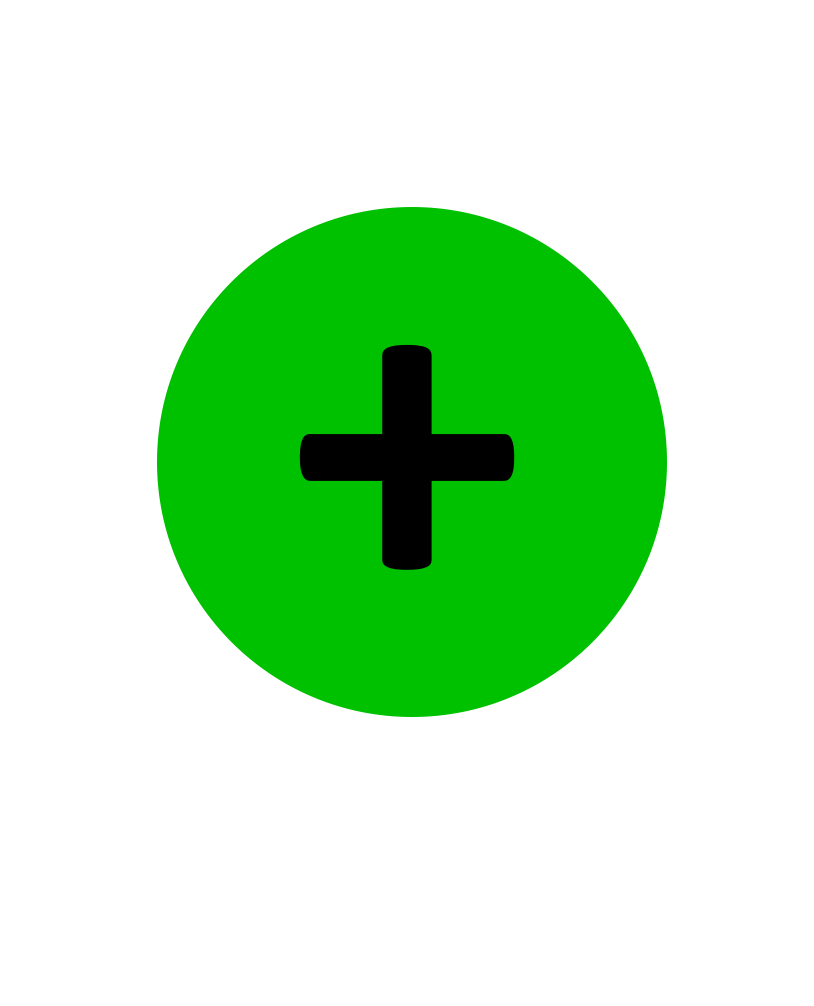 |
| **Lee, 2013** [18] | | 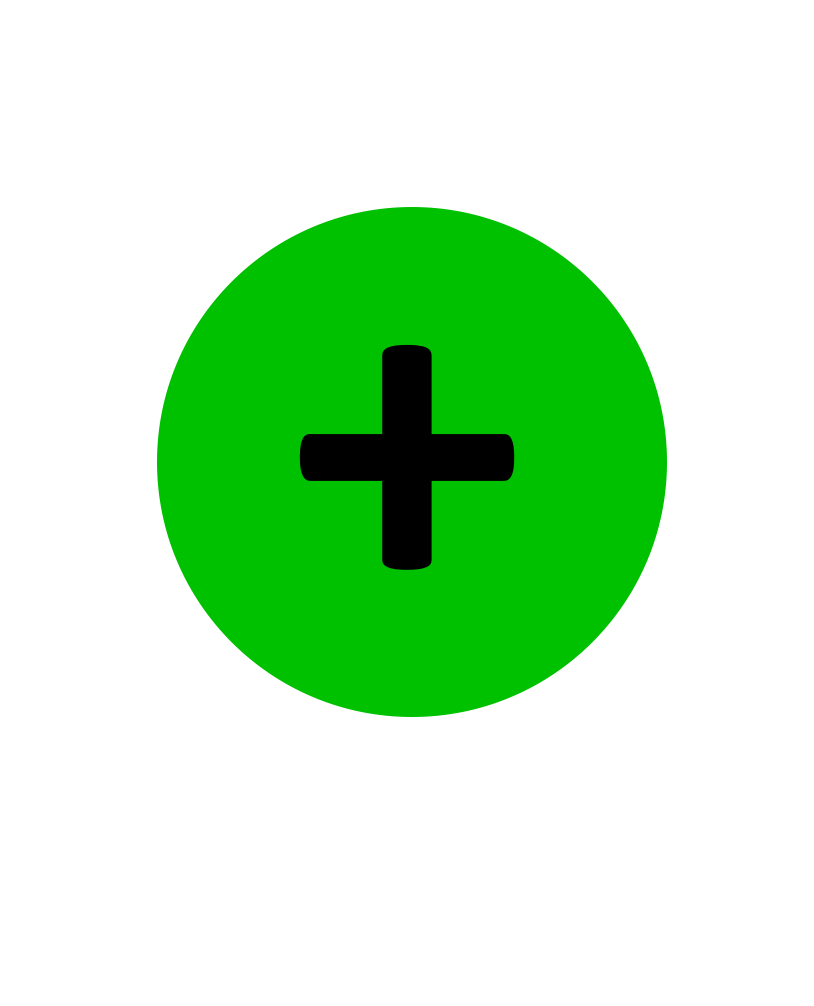 | 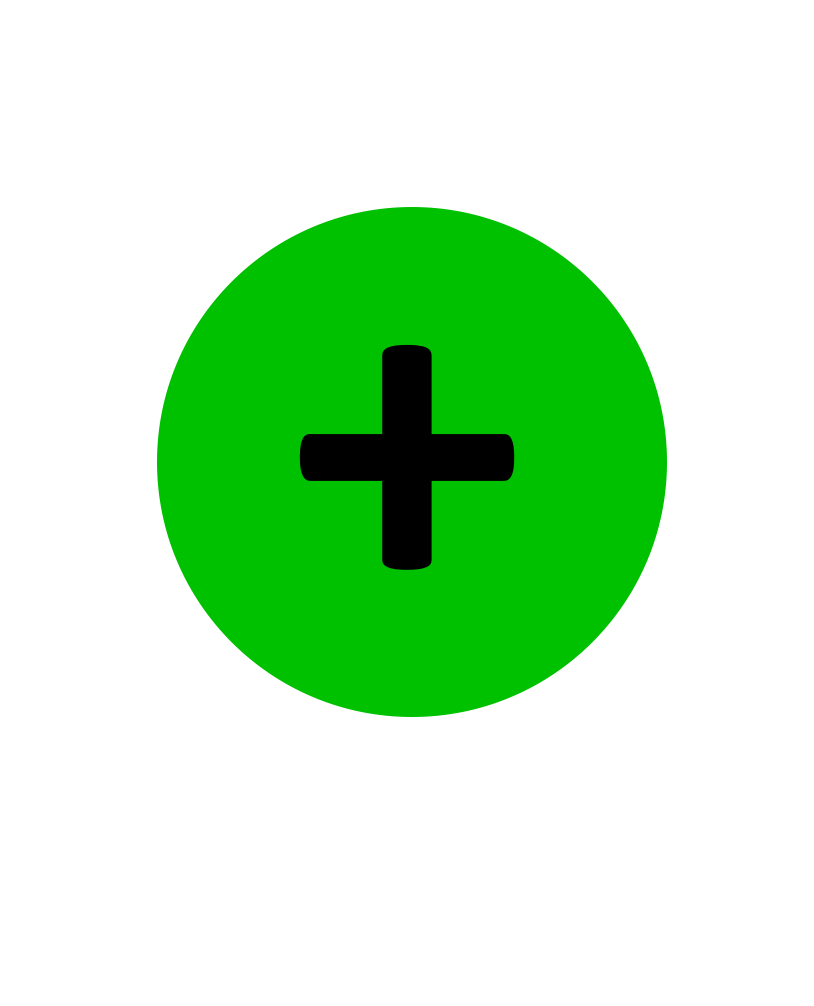 | 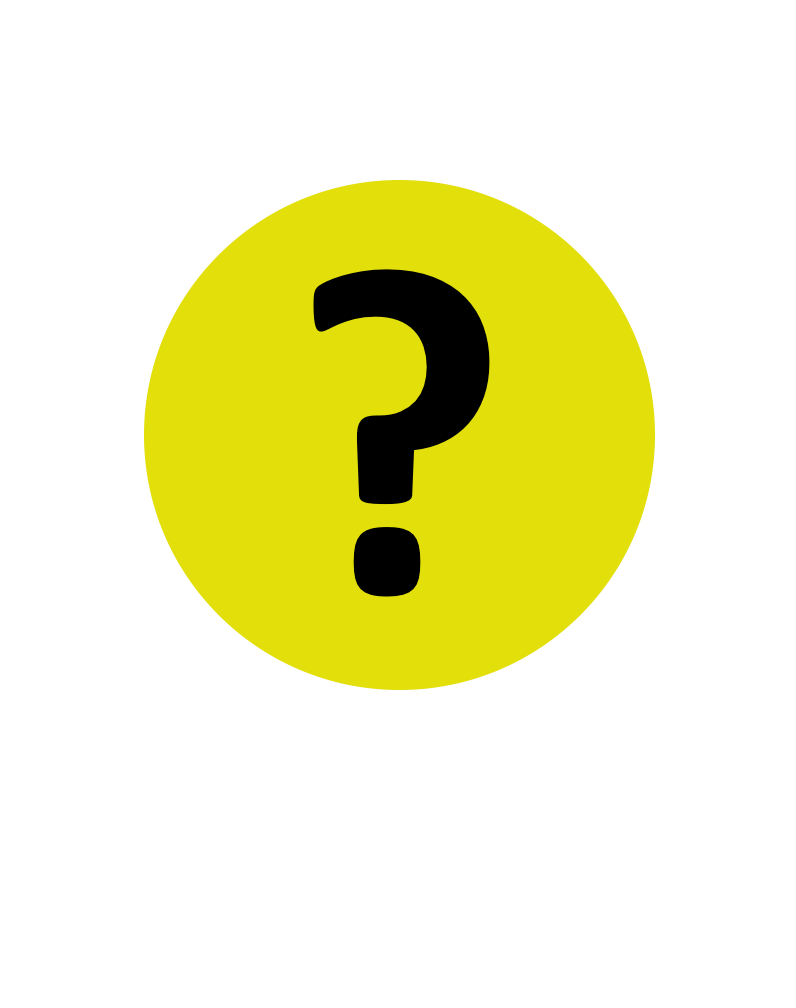 | 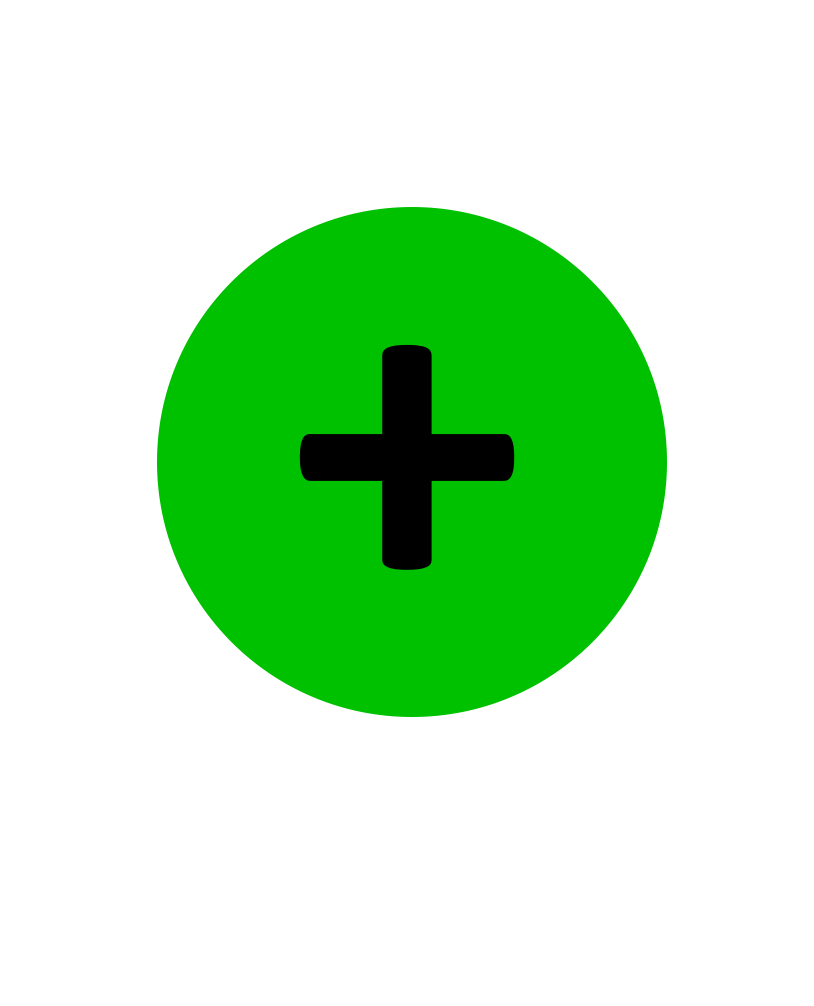 | 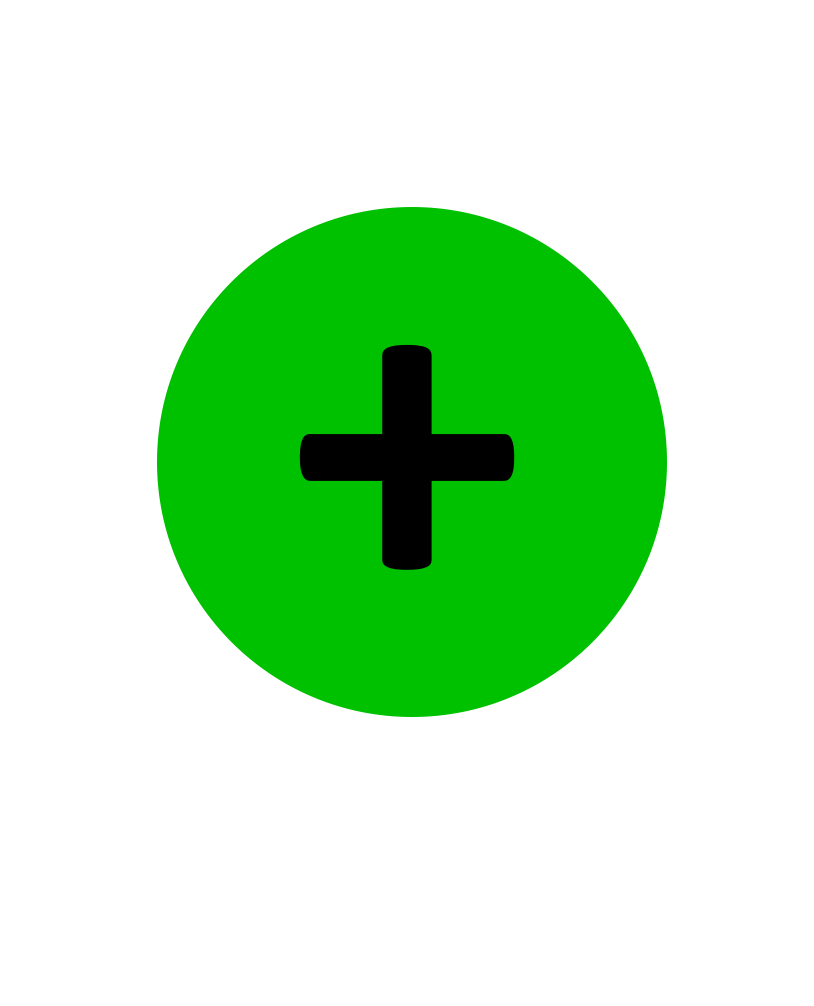 | 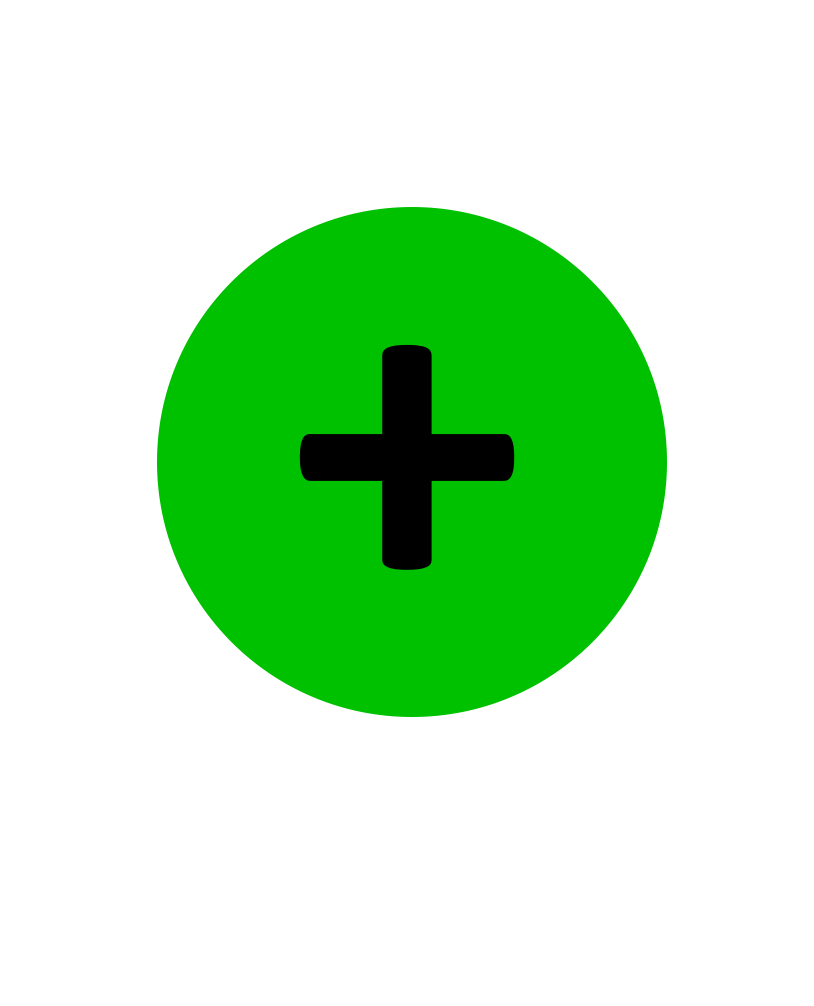 | 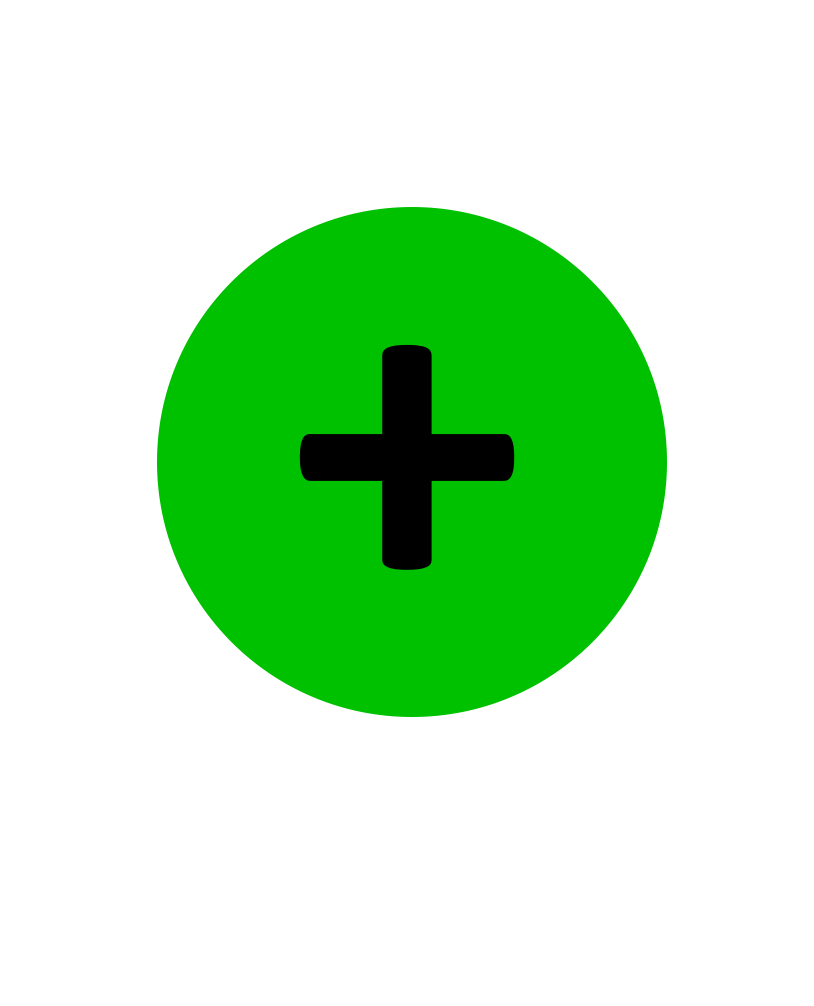 |
| **Lindgren, 2014** [19] | | 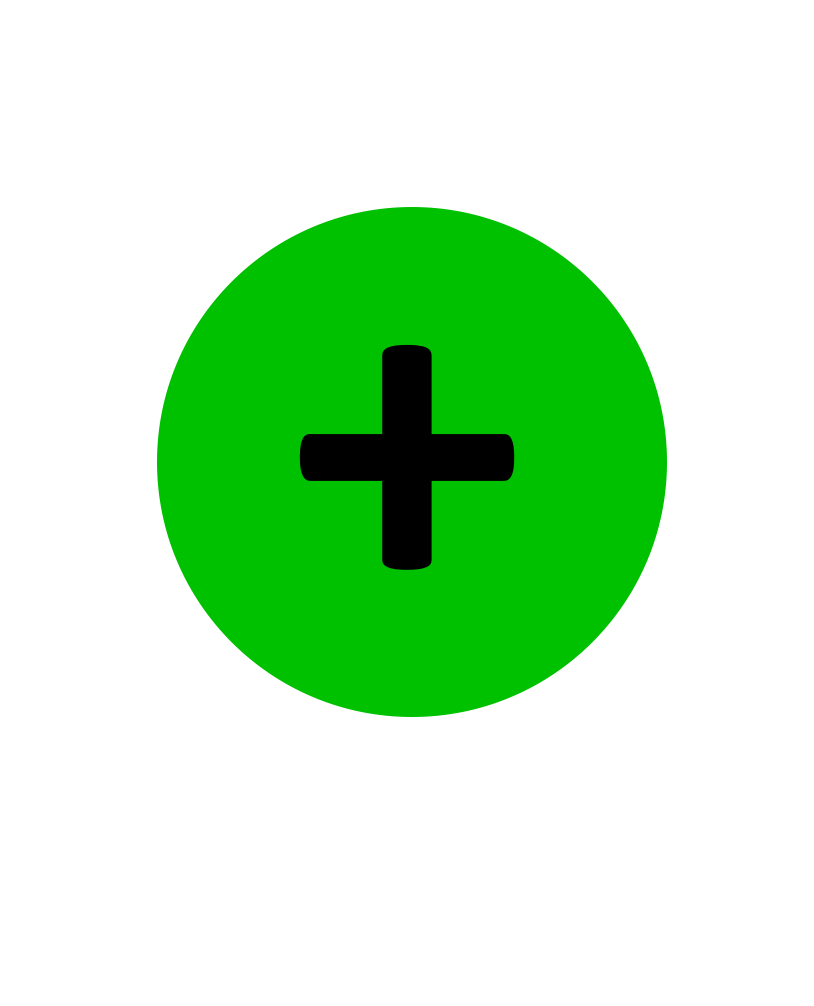 | 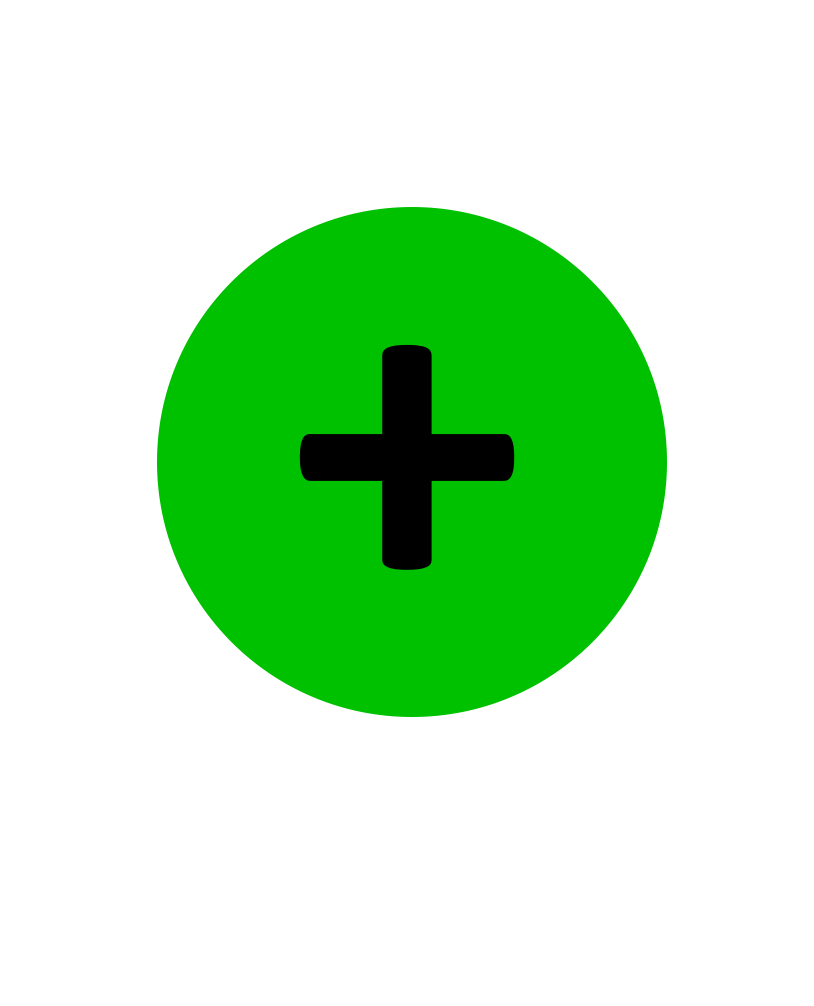 | 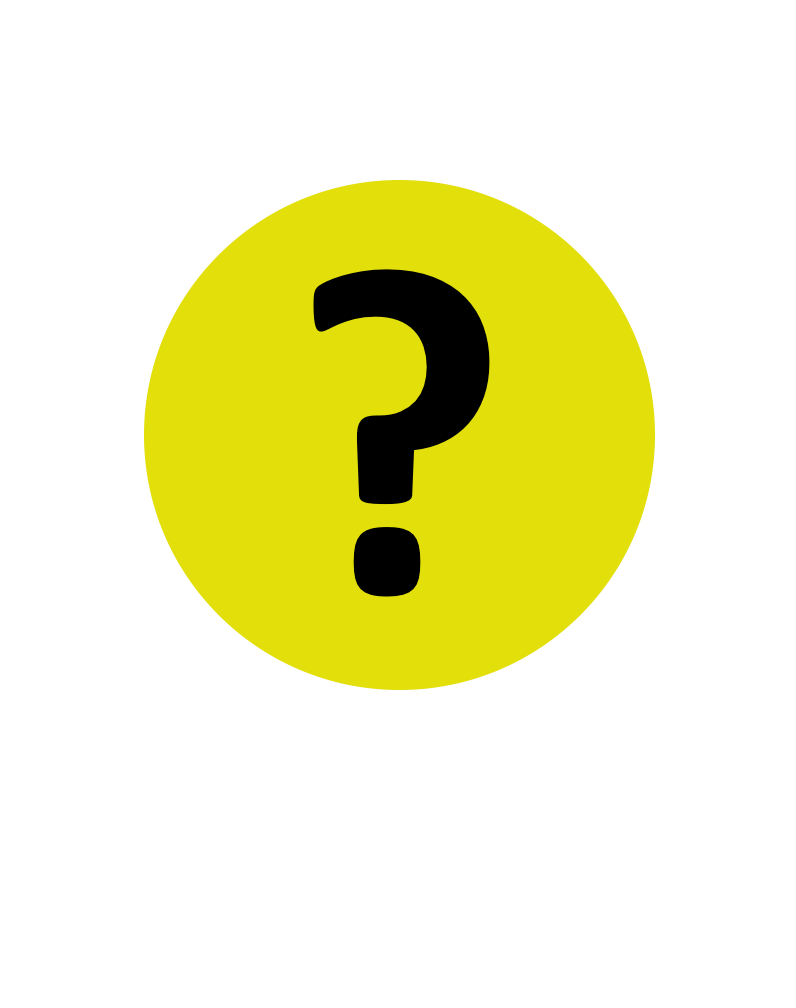 | 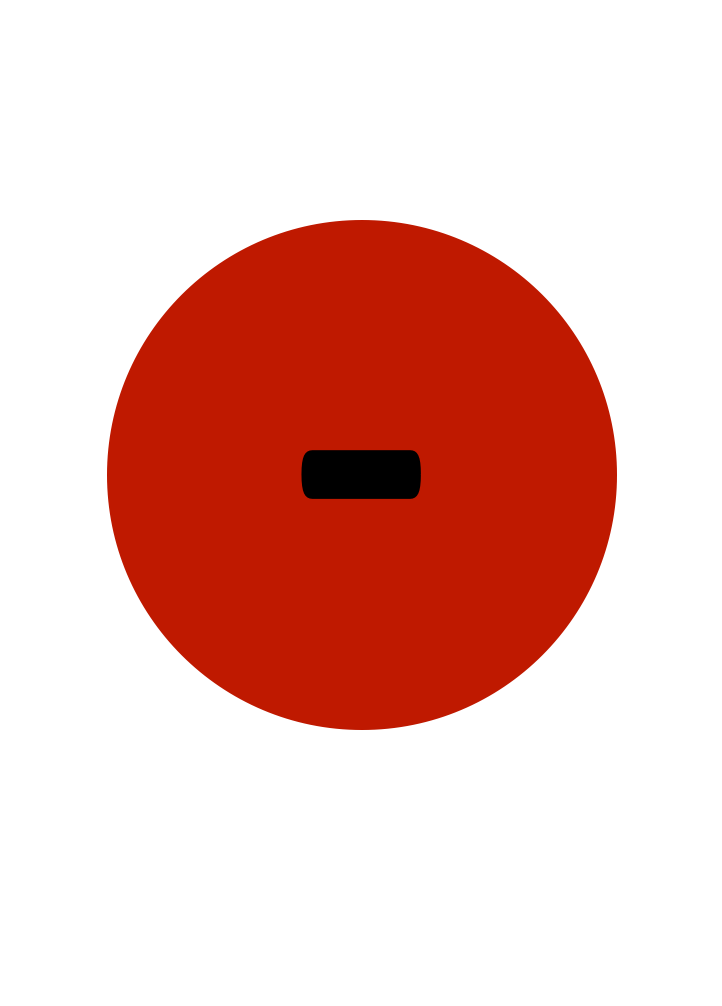 | 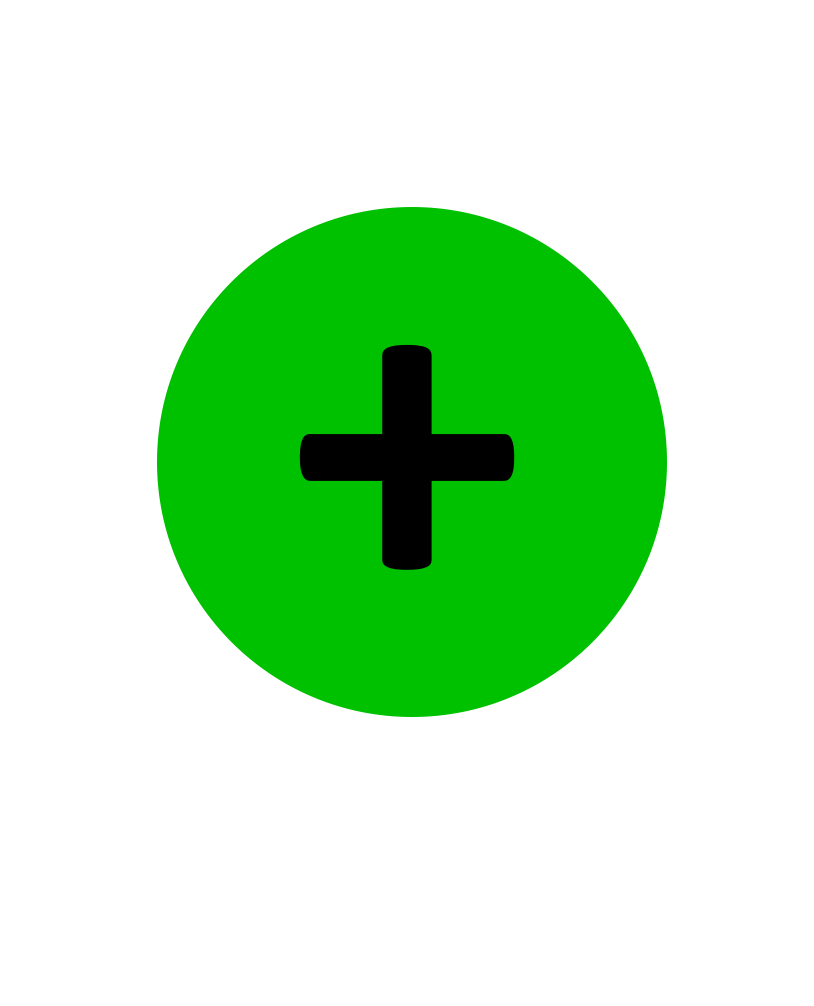 | 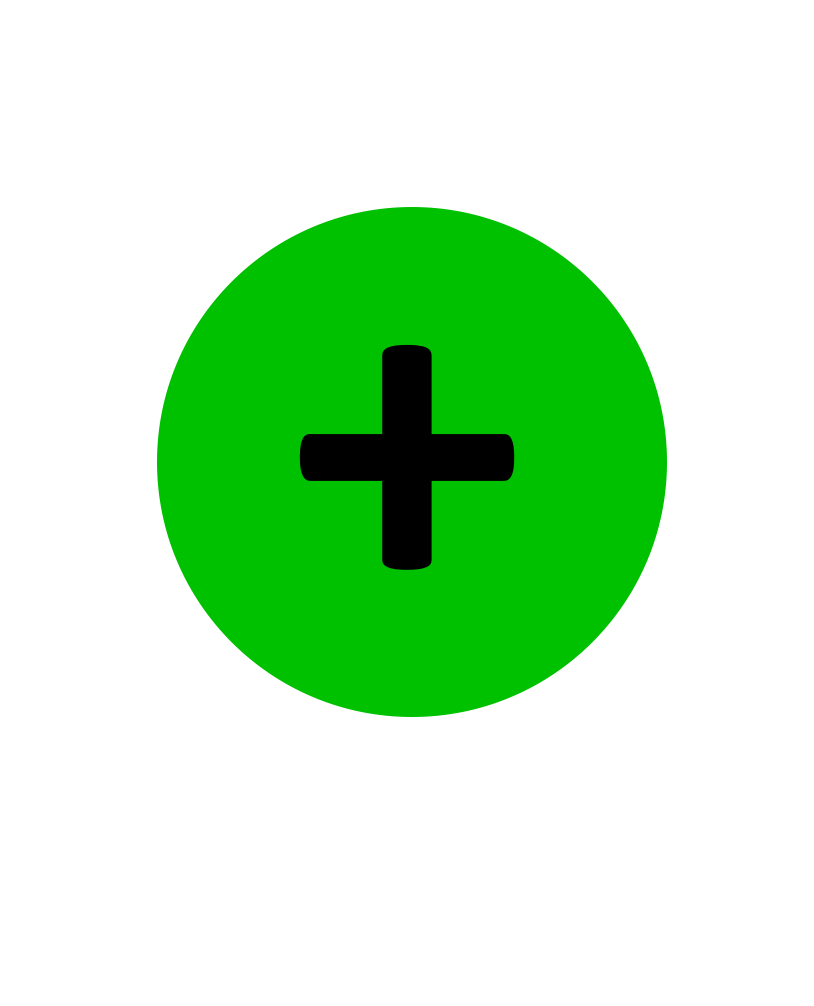 | 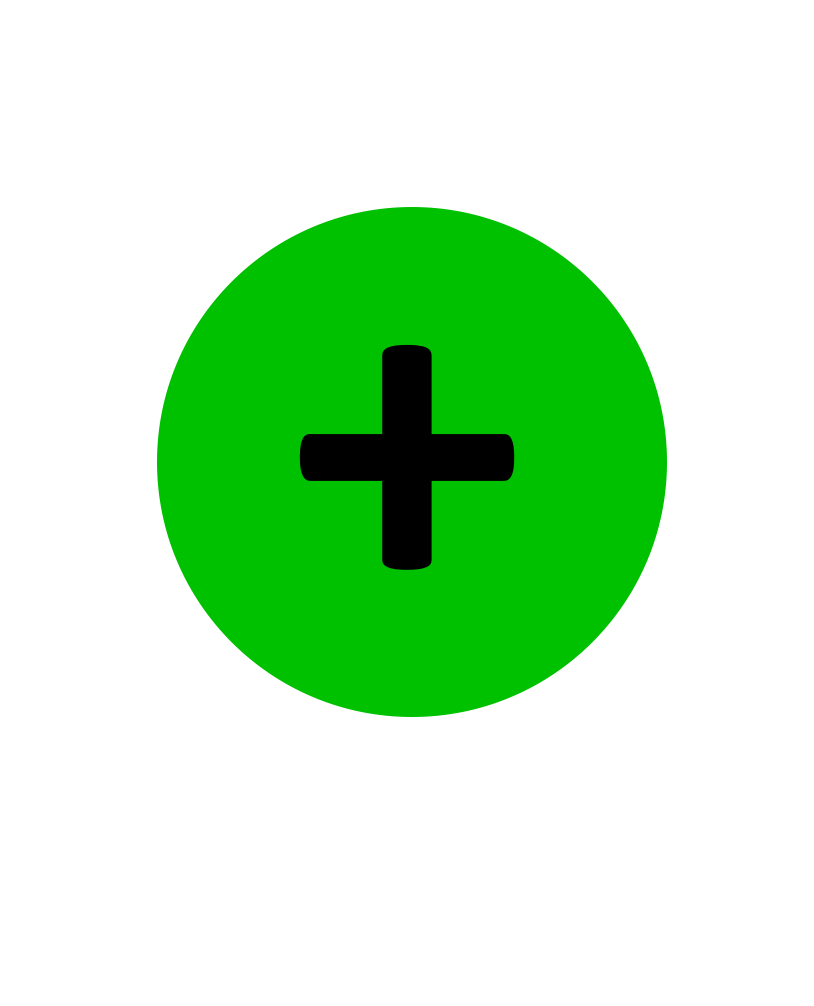 |
| **Schultze-Lutter, 2014** [20] | | 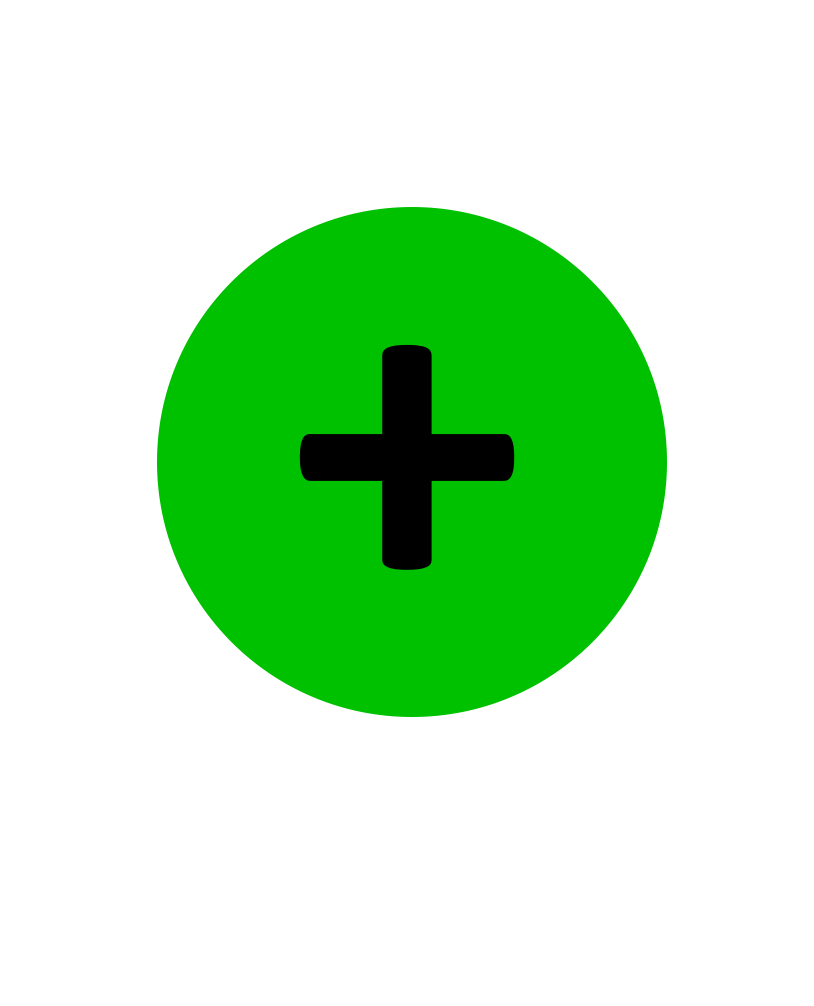 | 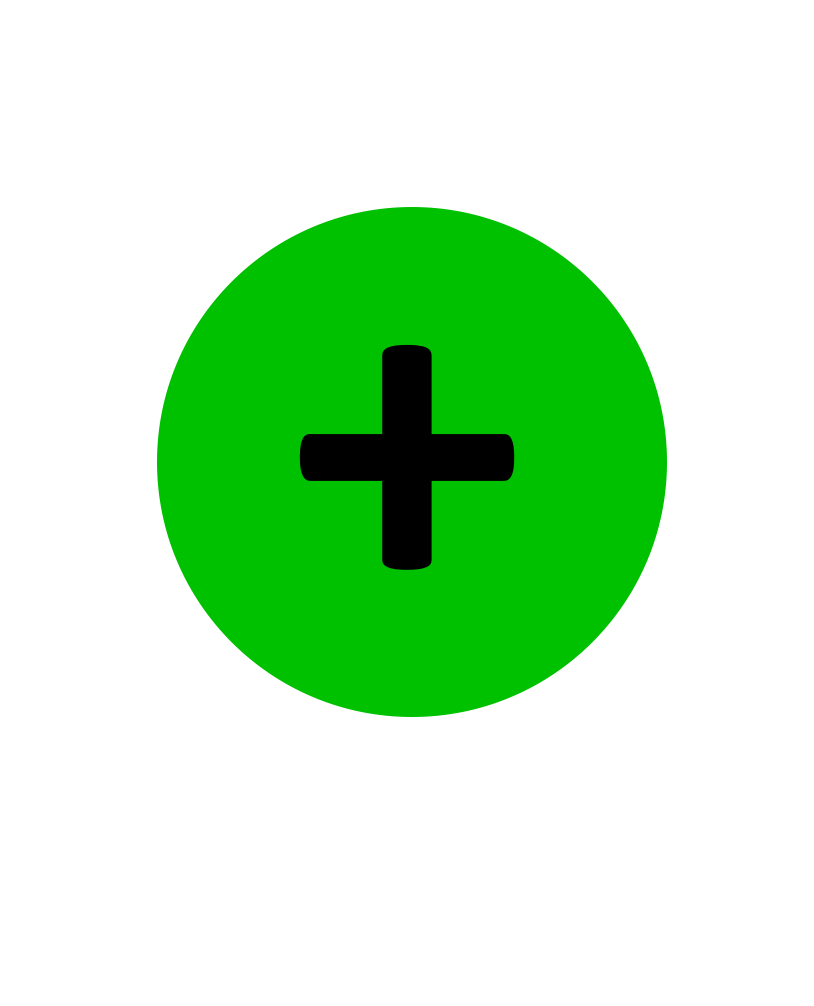 | 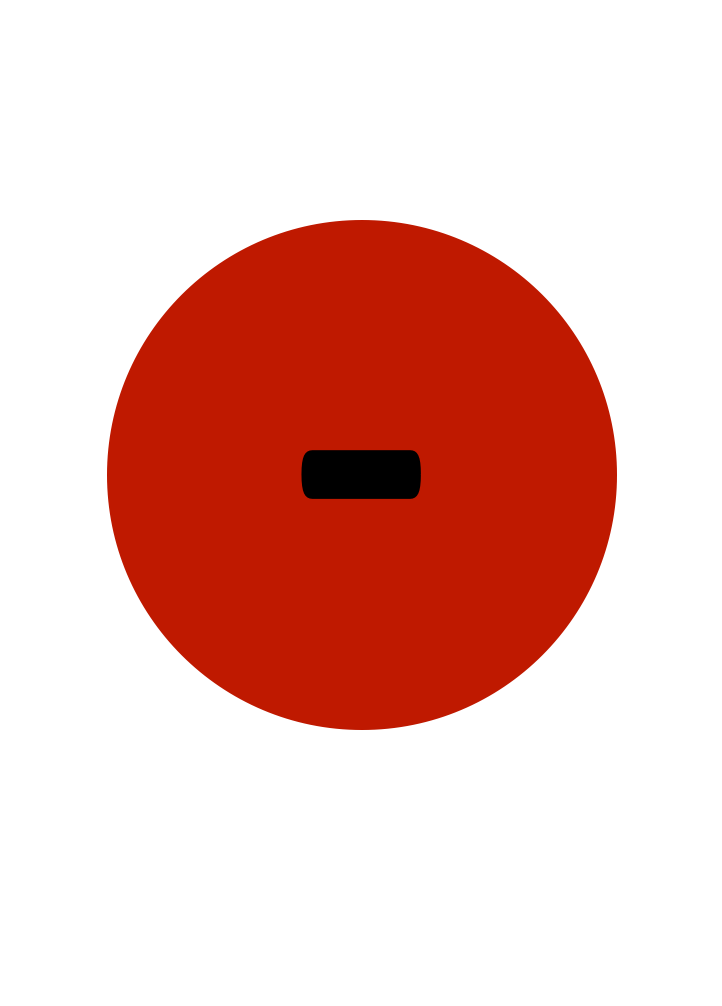 | 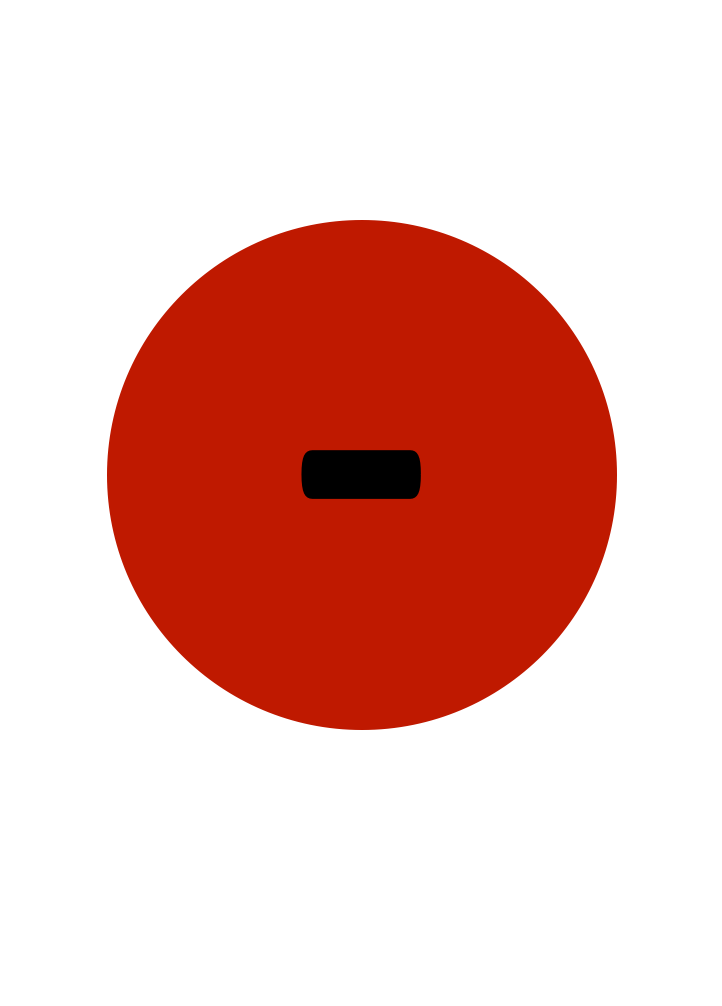 | 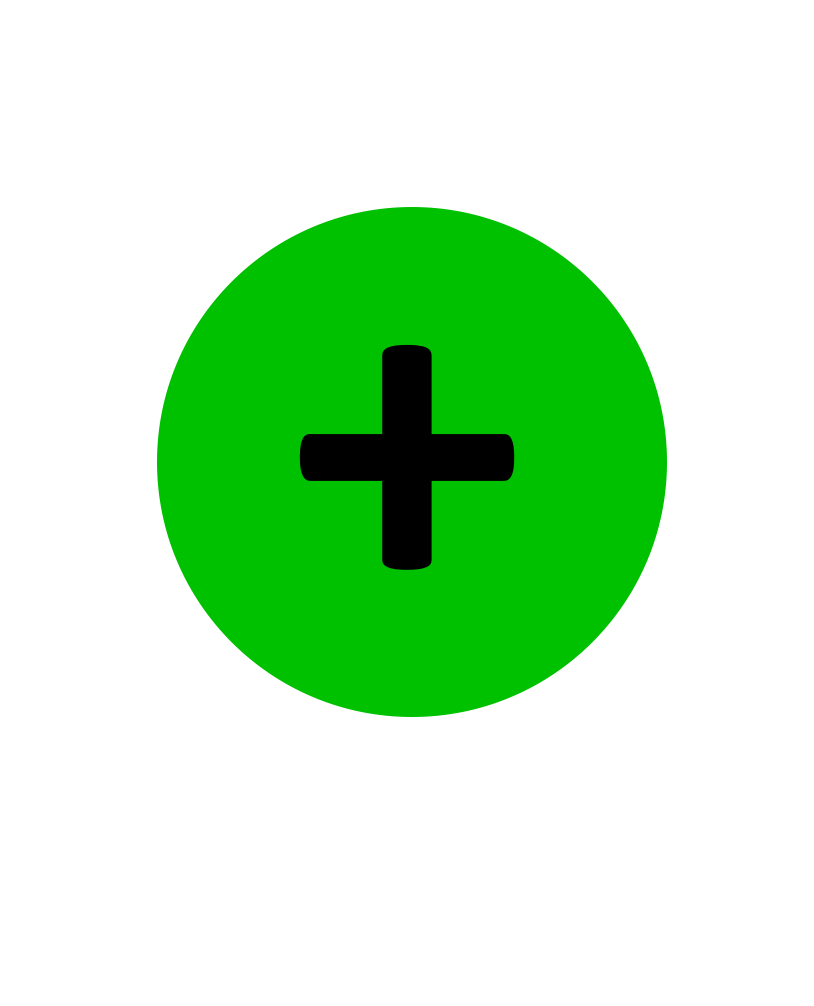 | 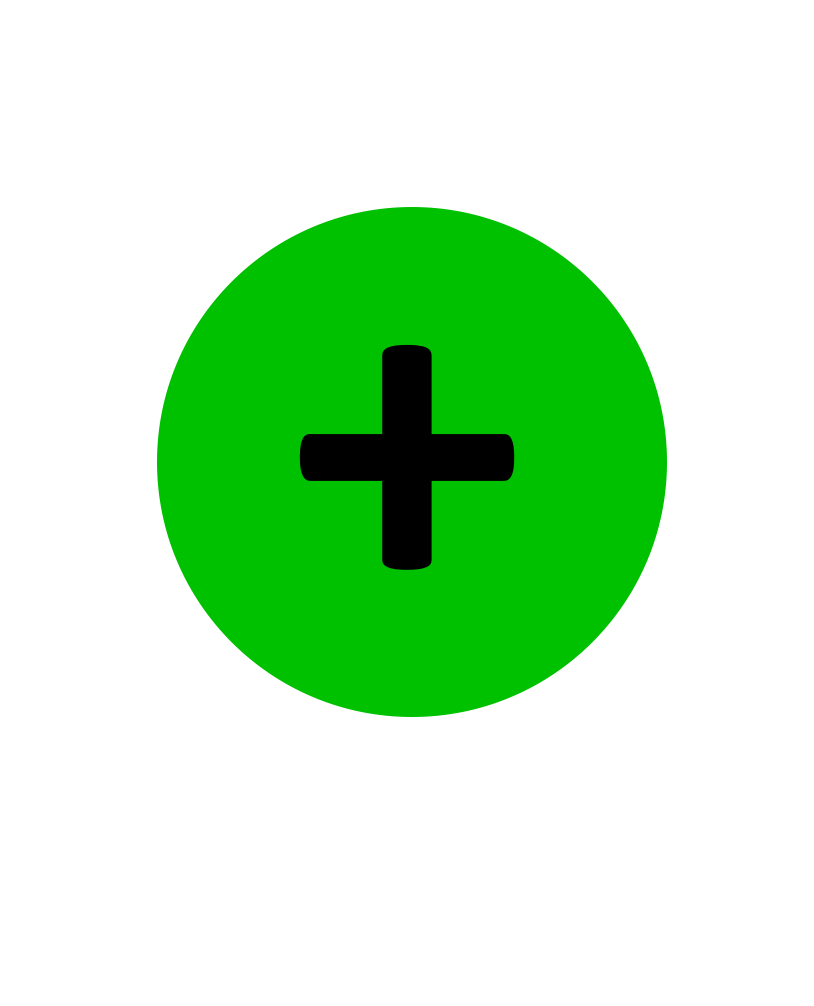 | 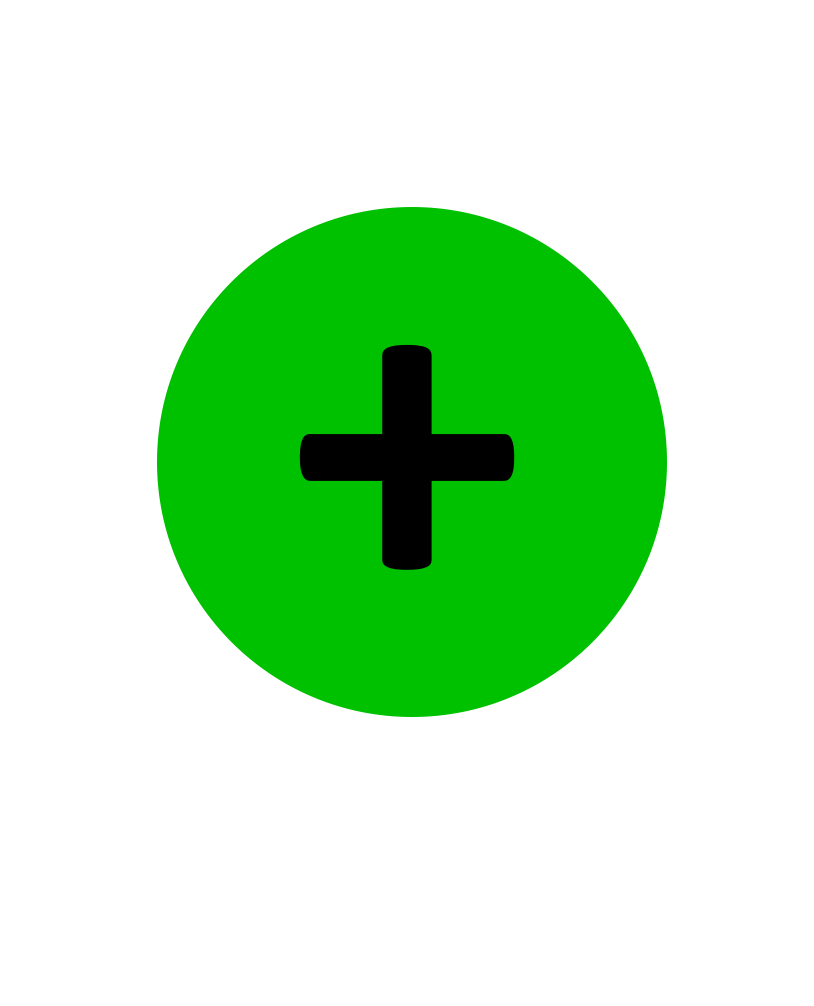 |
| **Kline, 2015** [21] | | 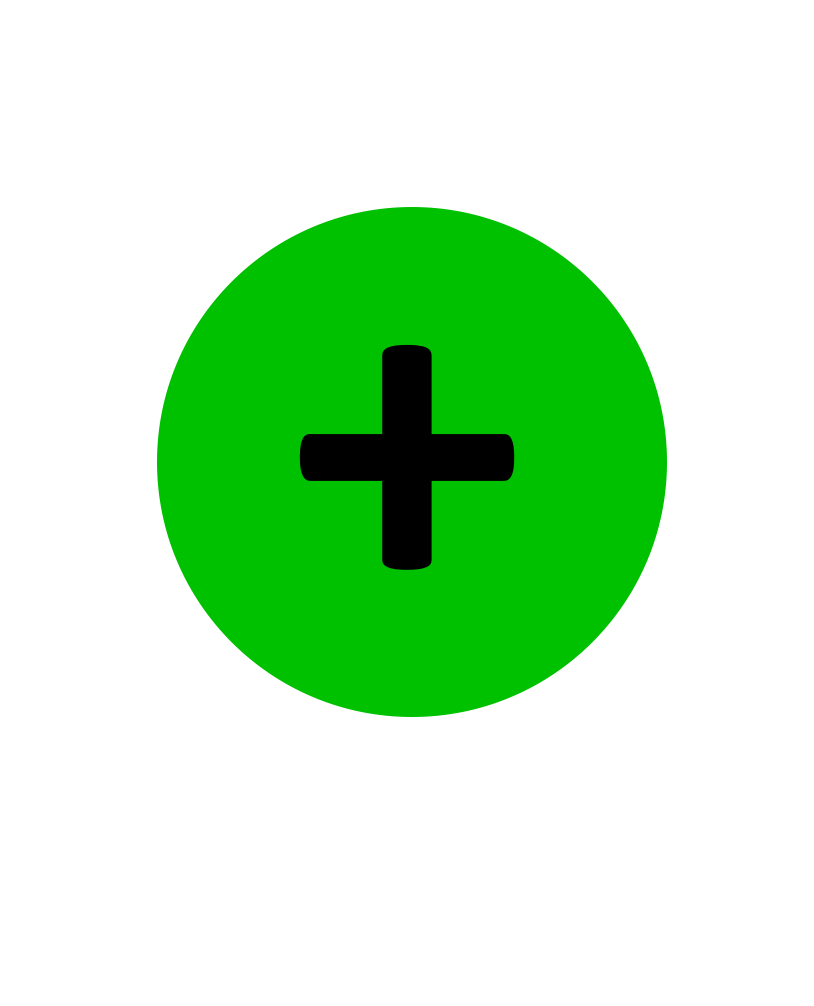 | 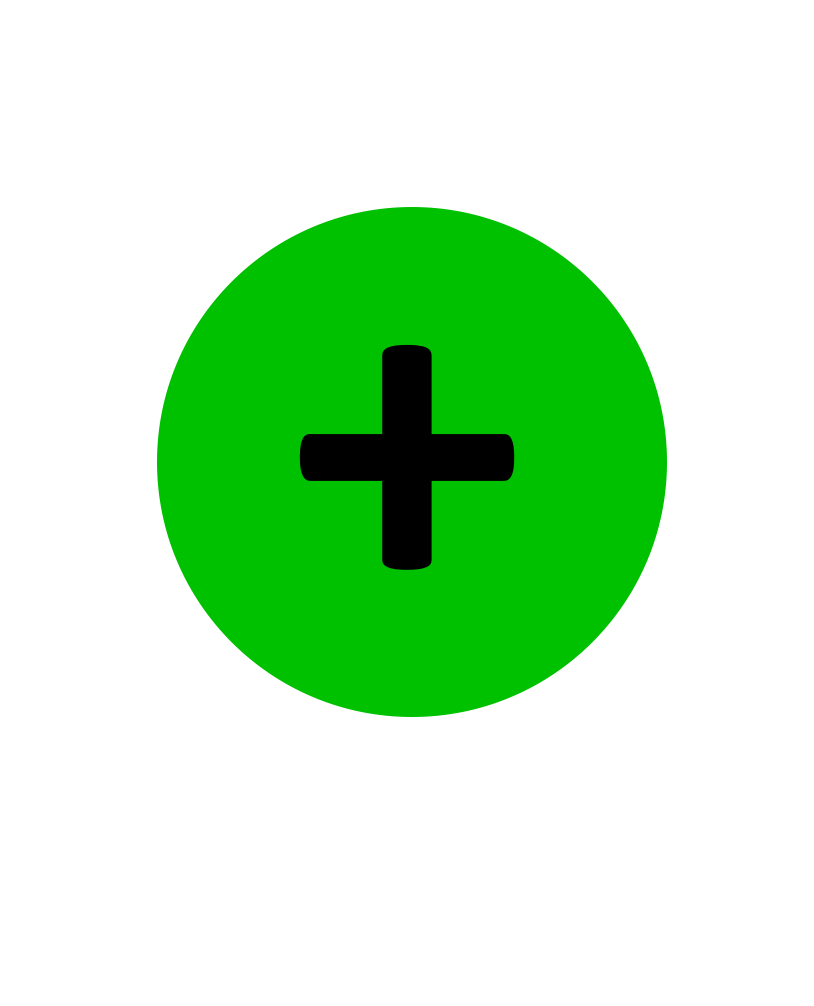 | 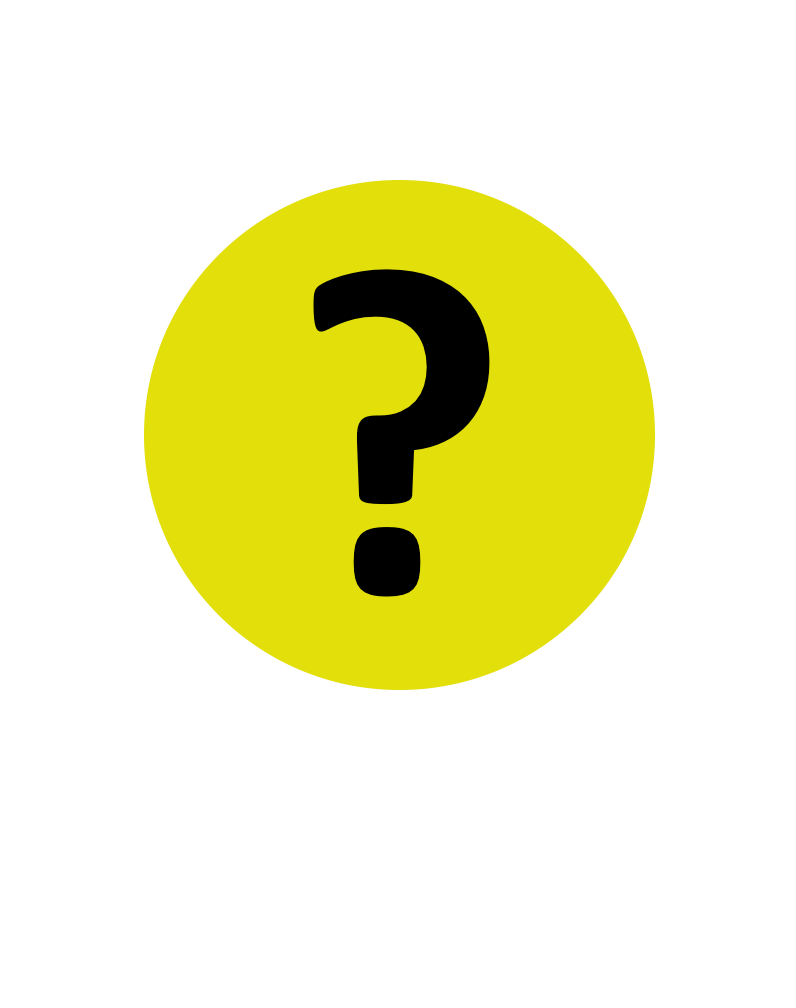 | 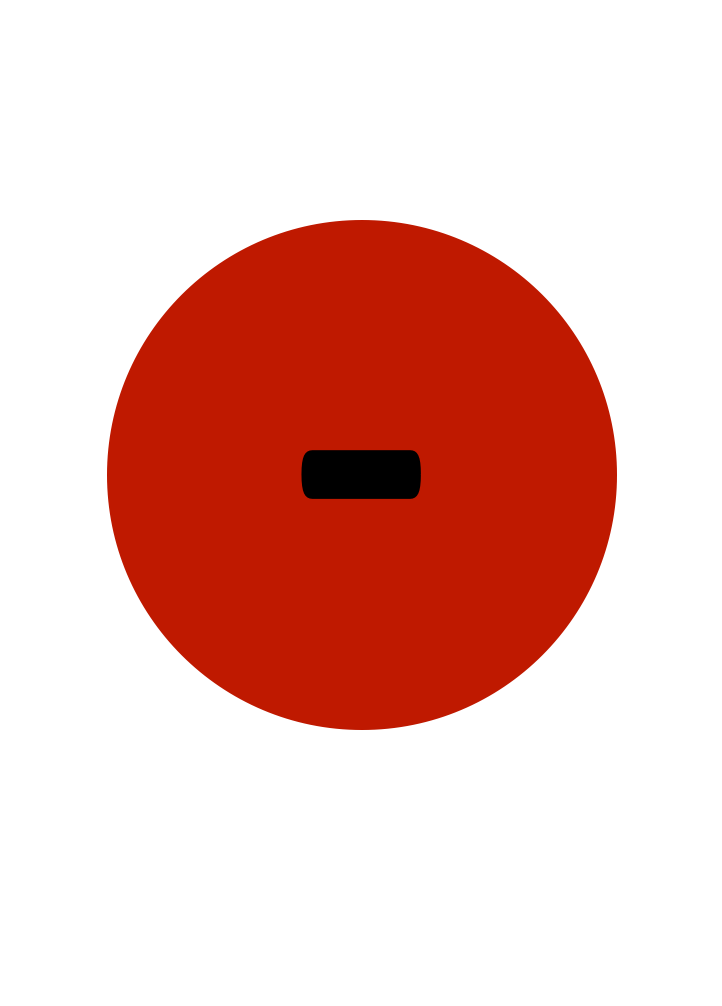 | 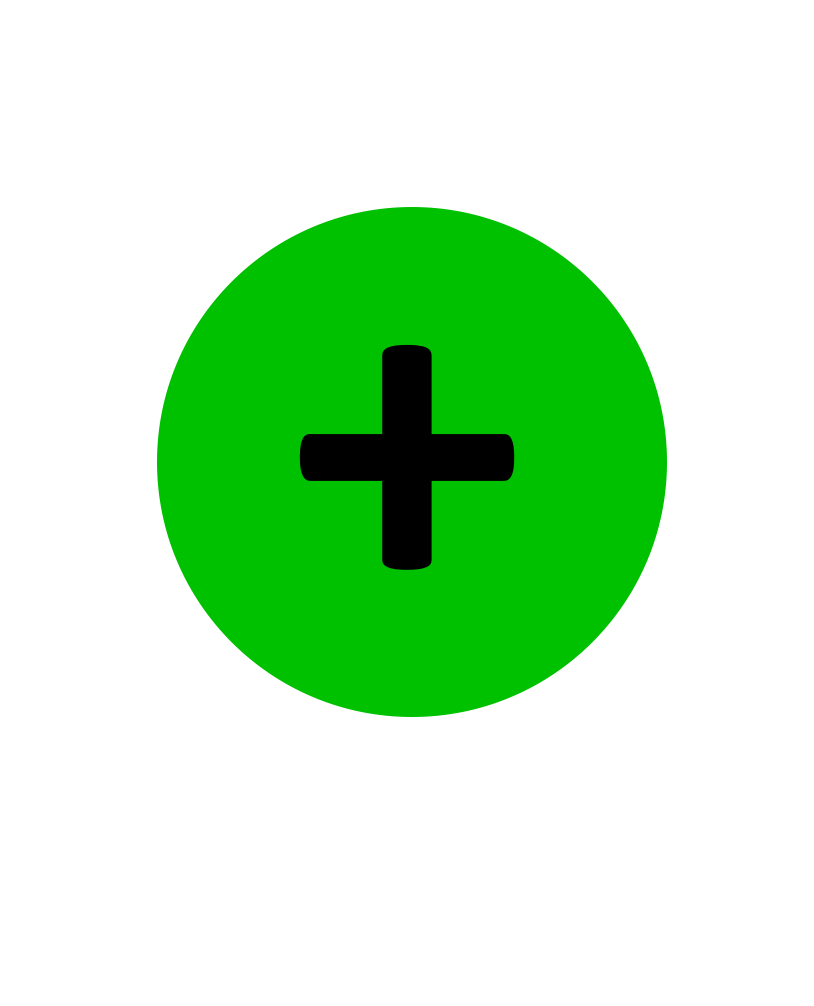 | 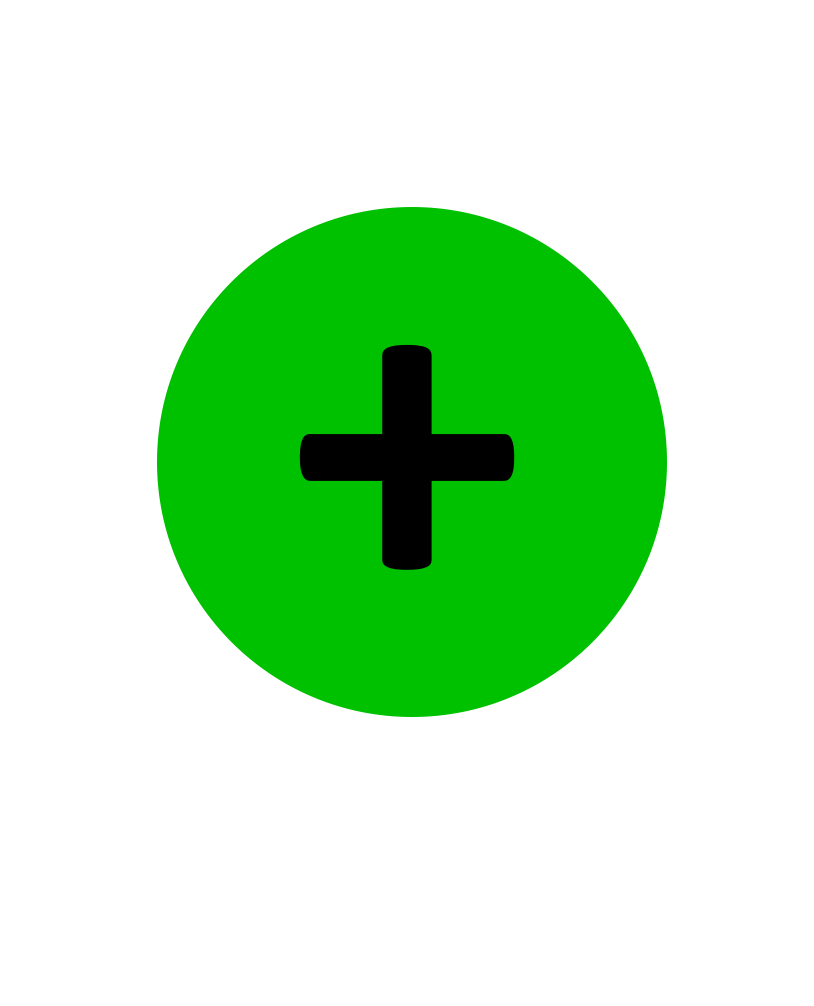 | 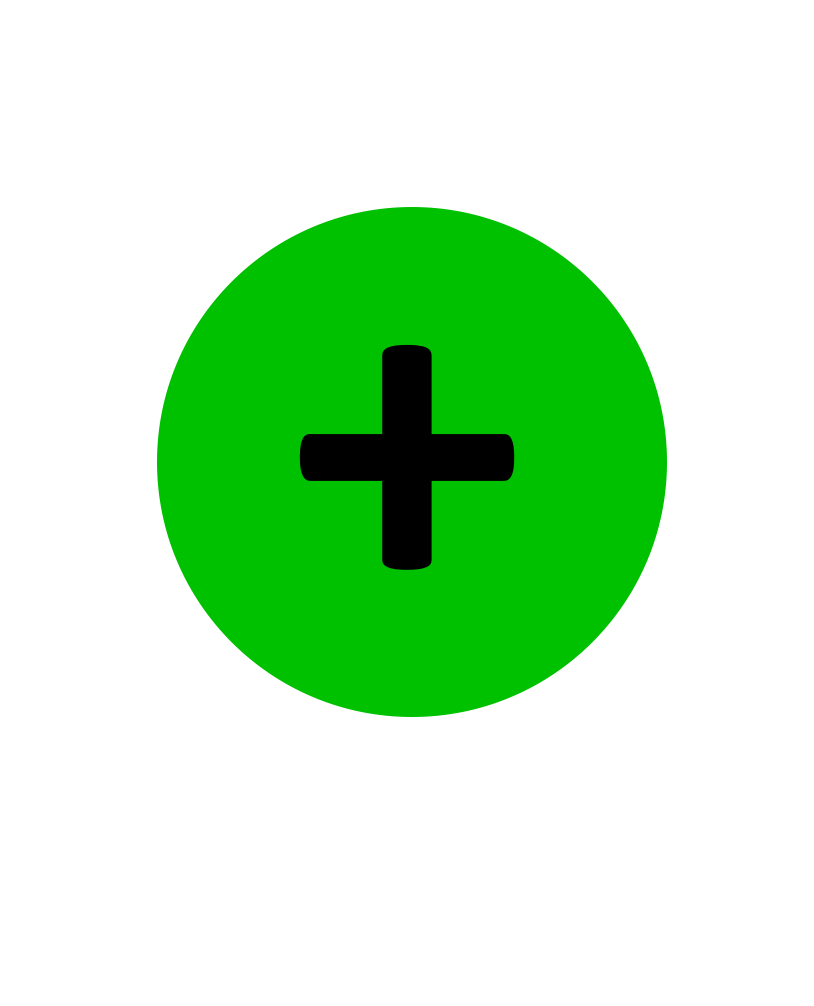 |
| **Kotlicka-Antczak, 2015** [22] | | 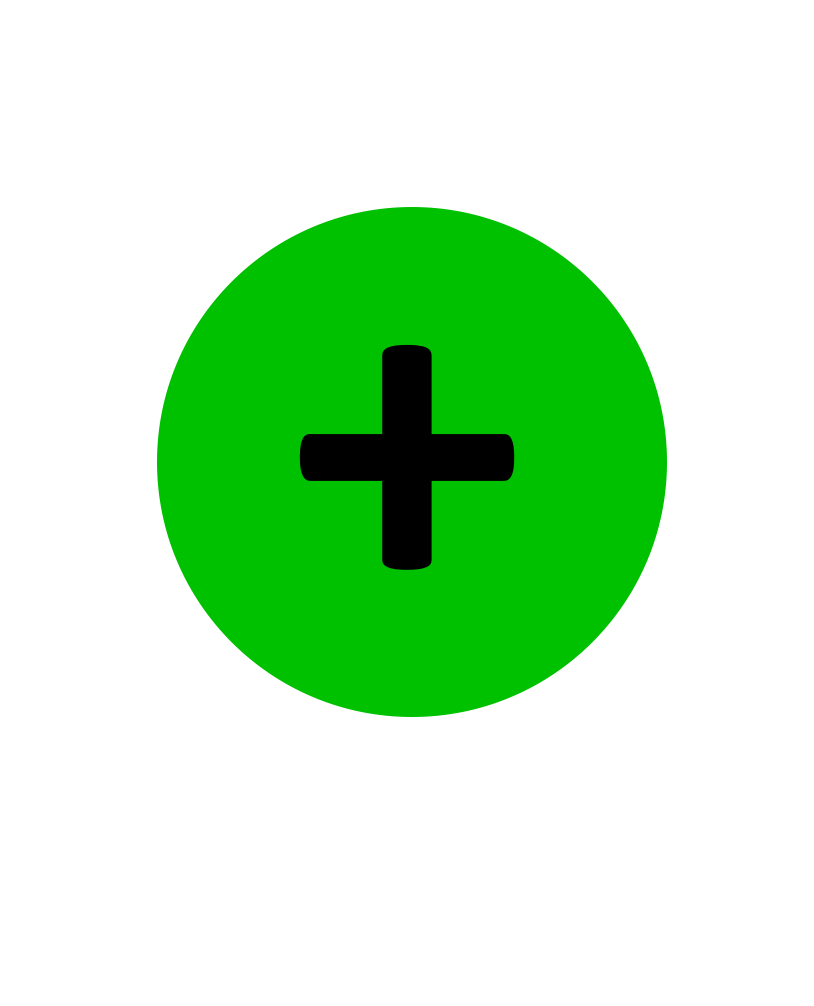 | 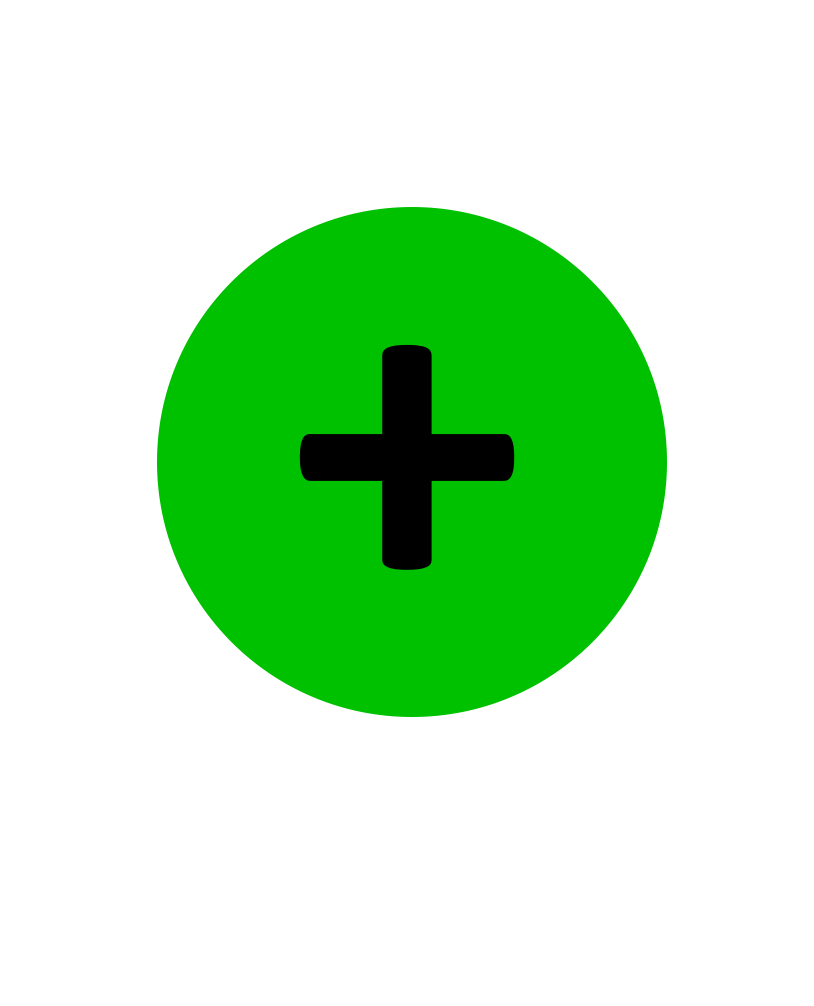 | 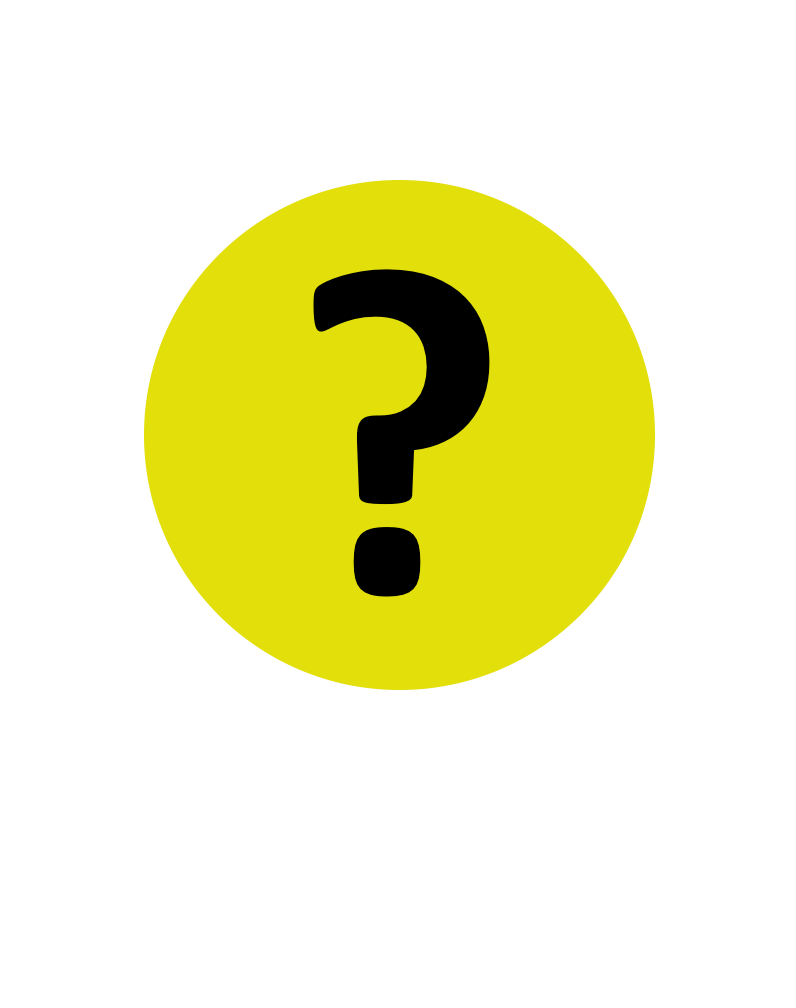 | 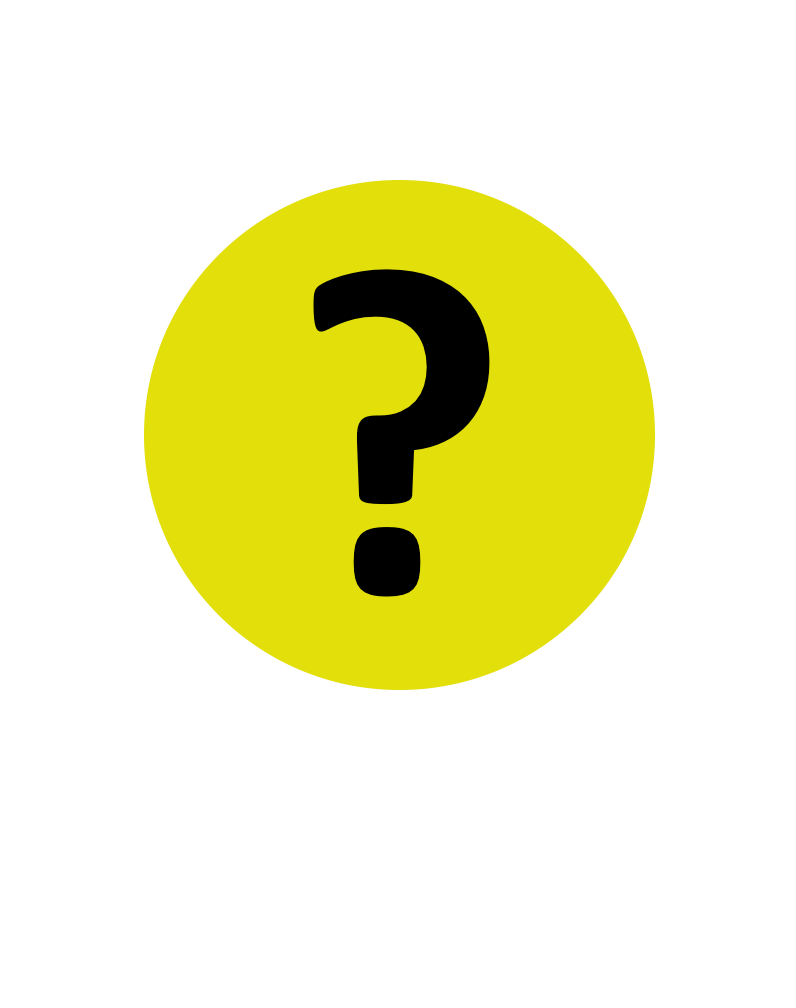 | 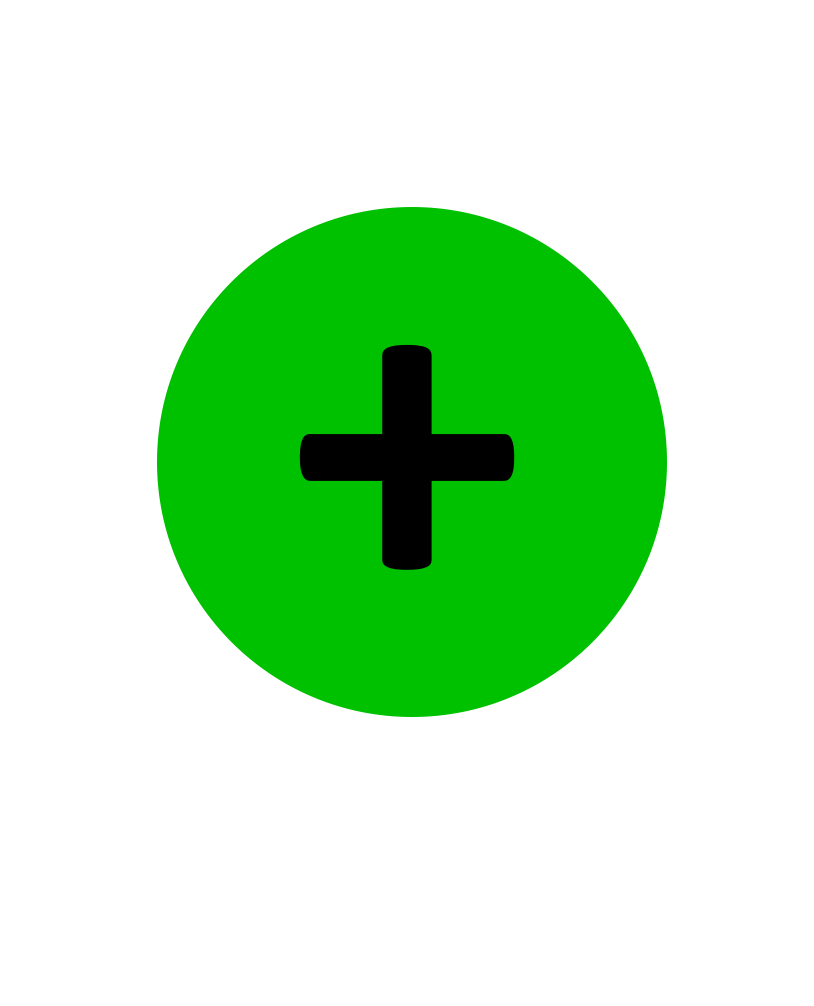 | 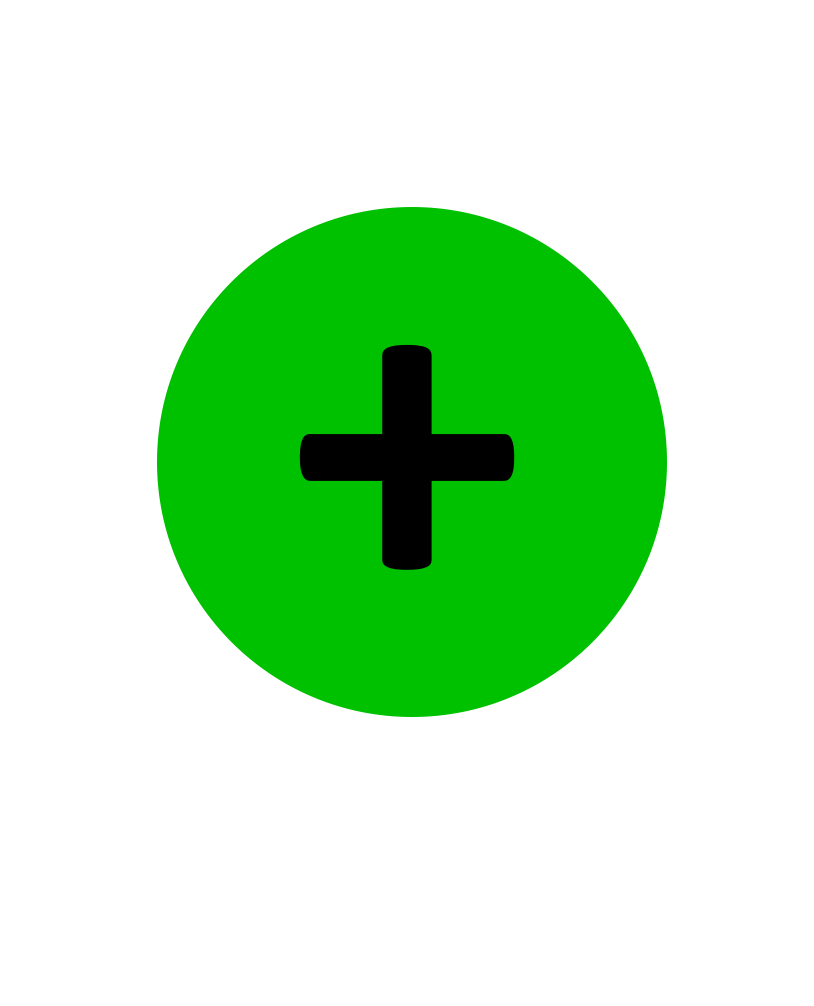 | 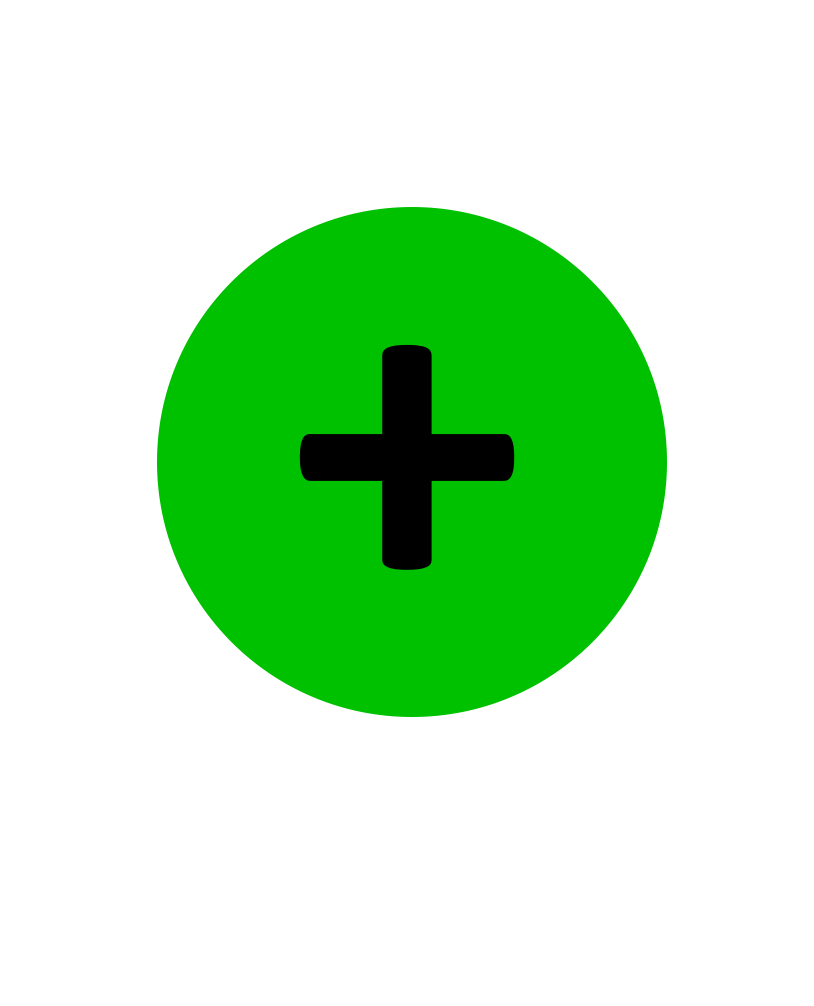 |
| **Francesconi, 2017** [23] | | 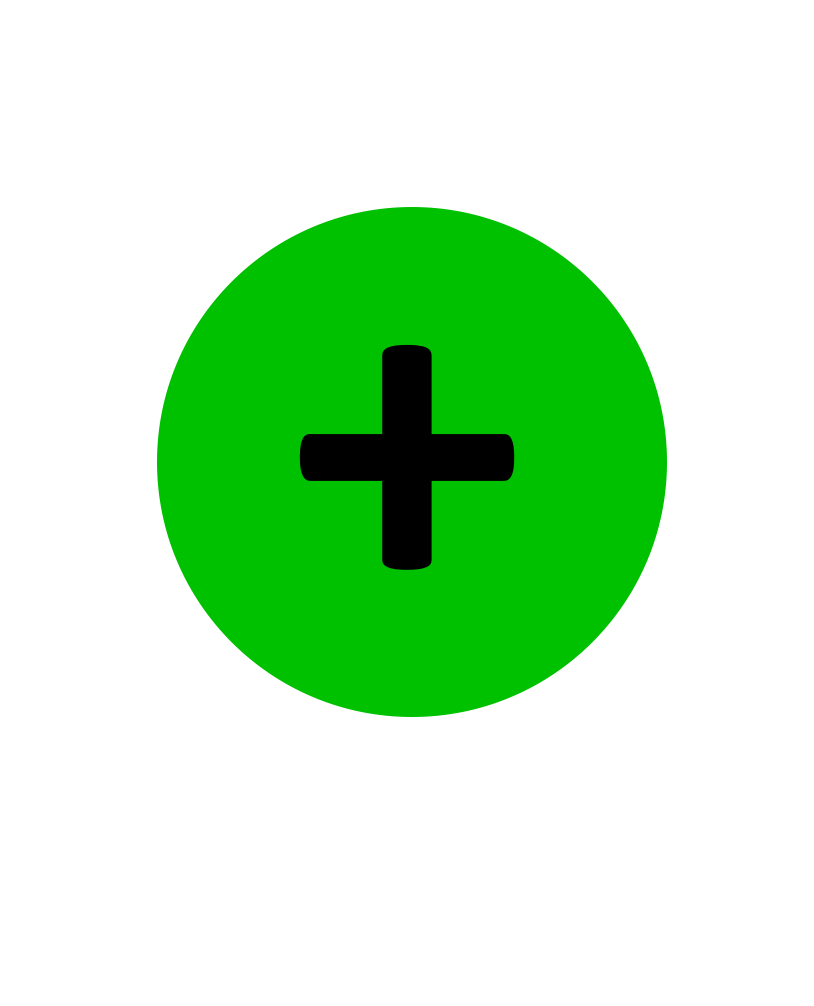 | 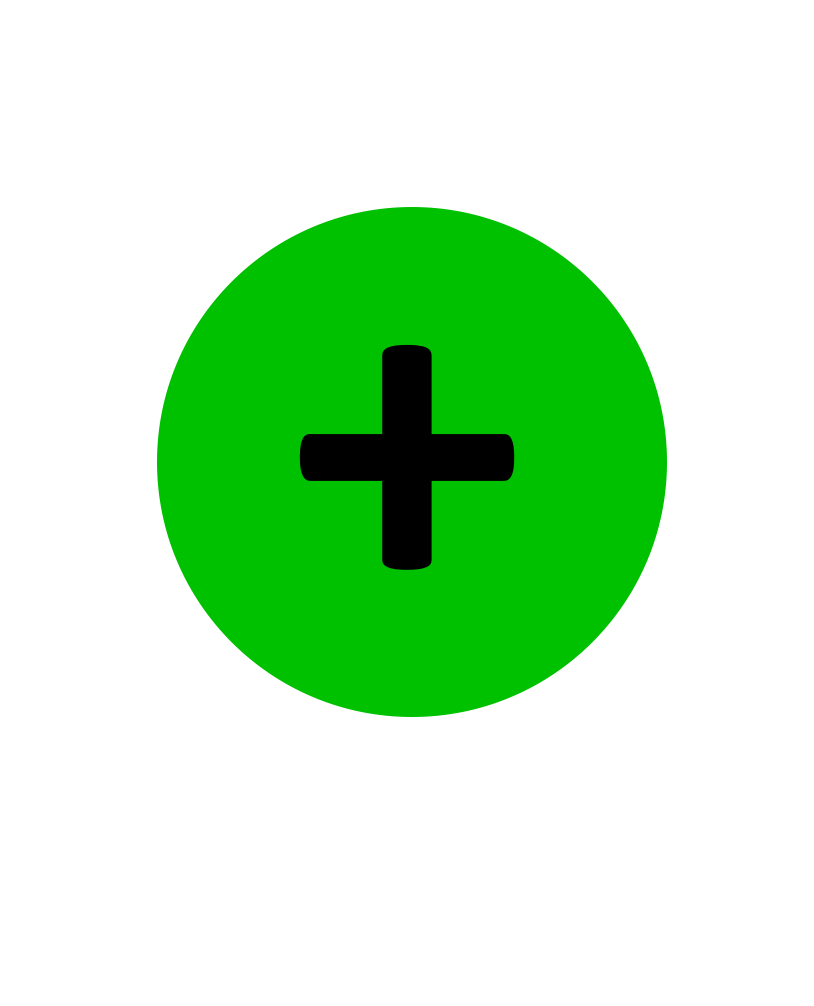 | 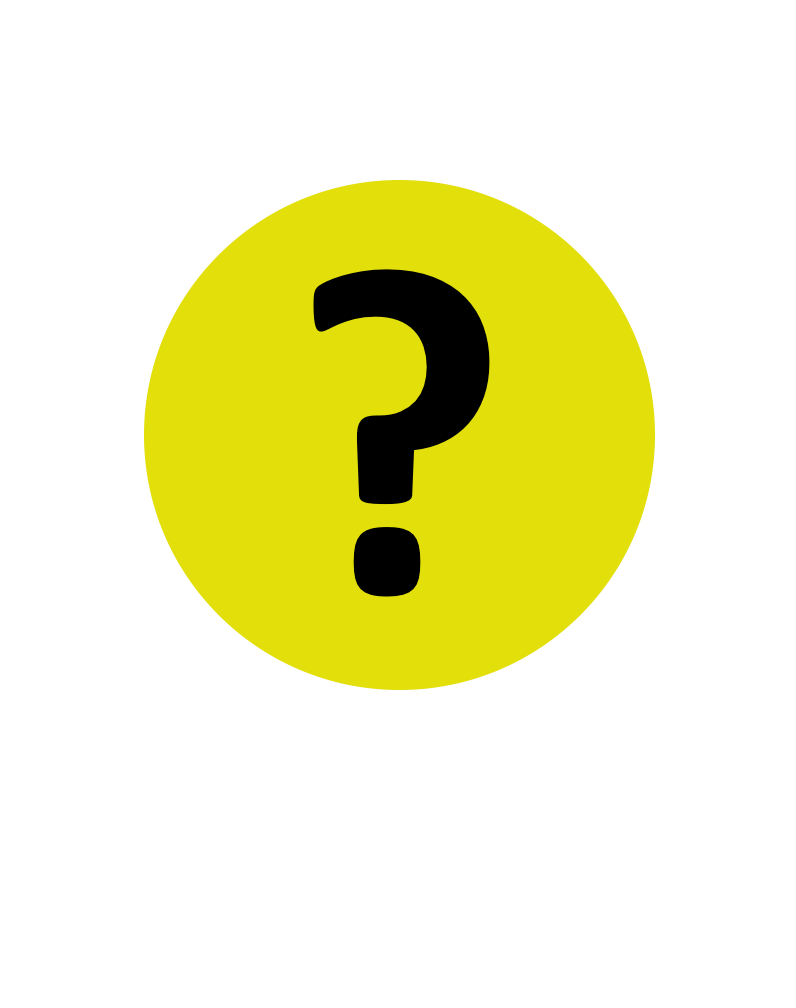 | 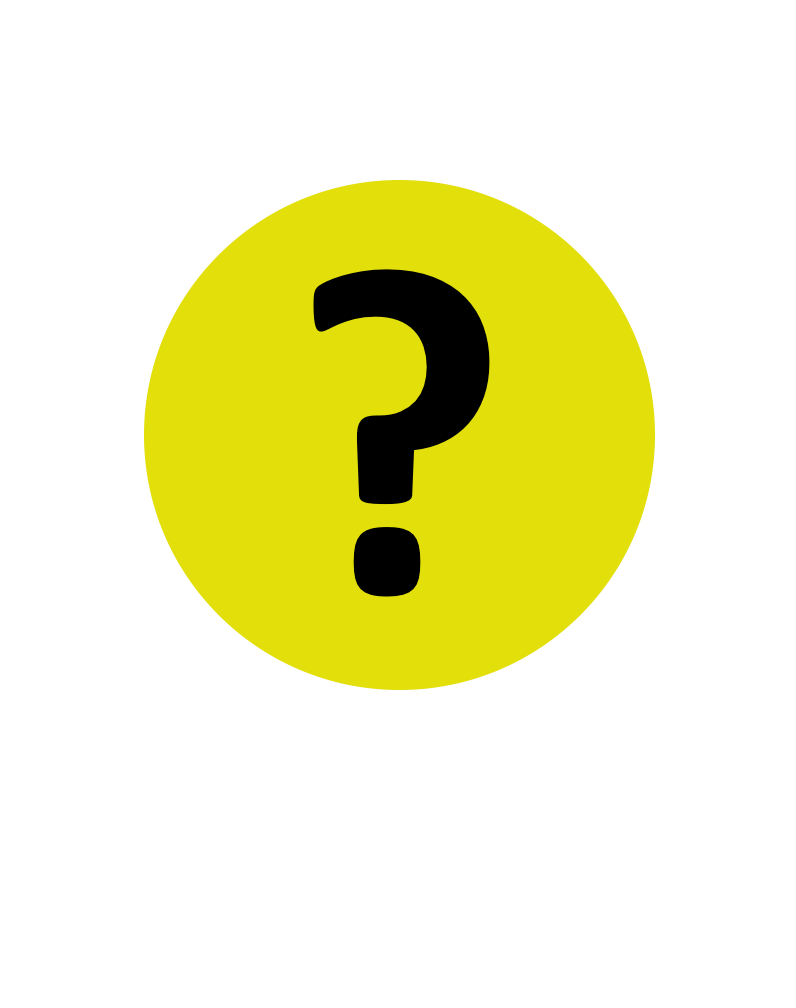 | 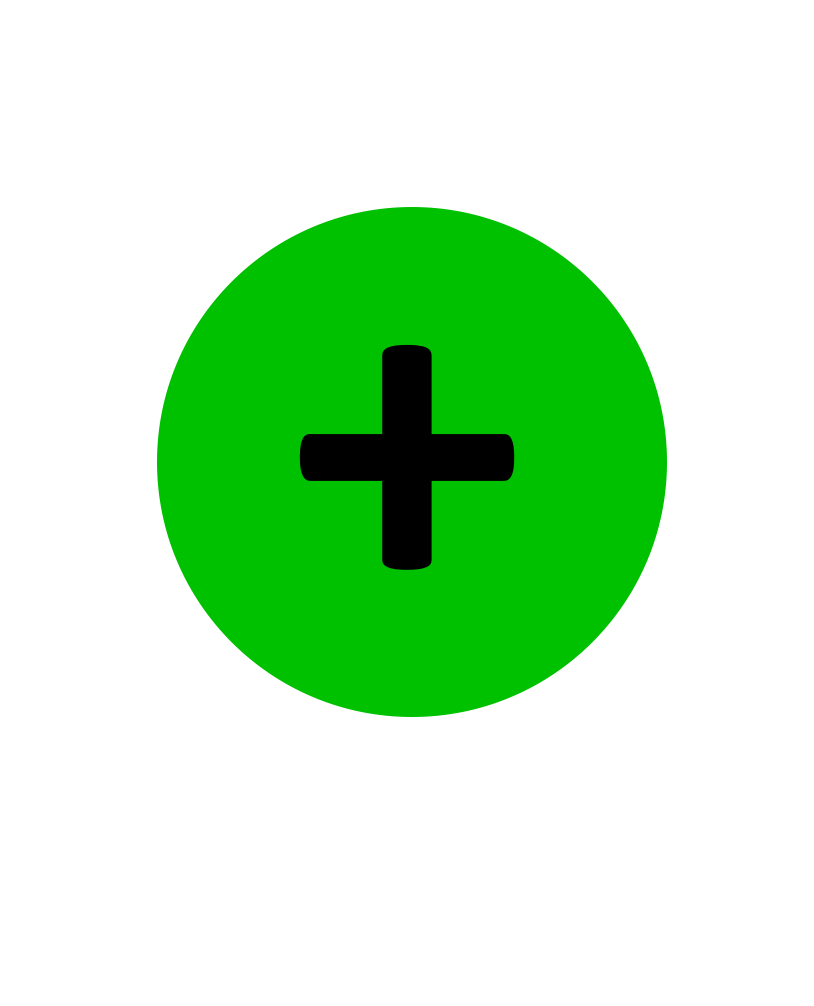 | 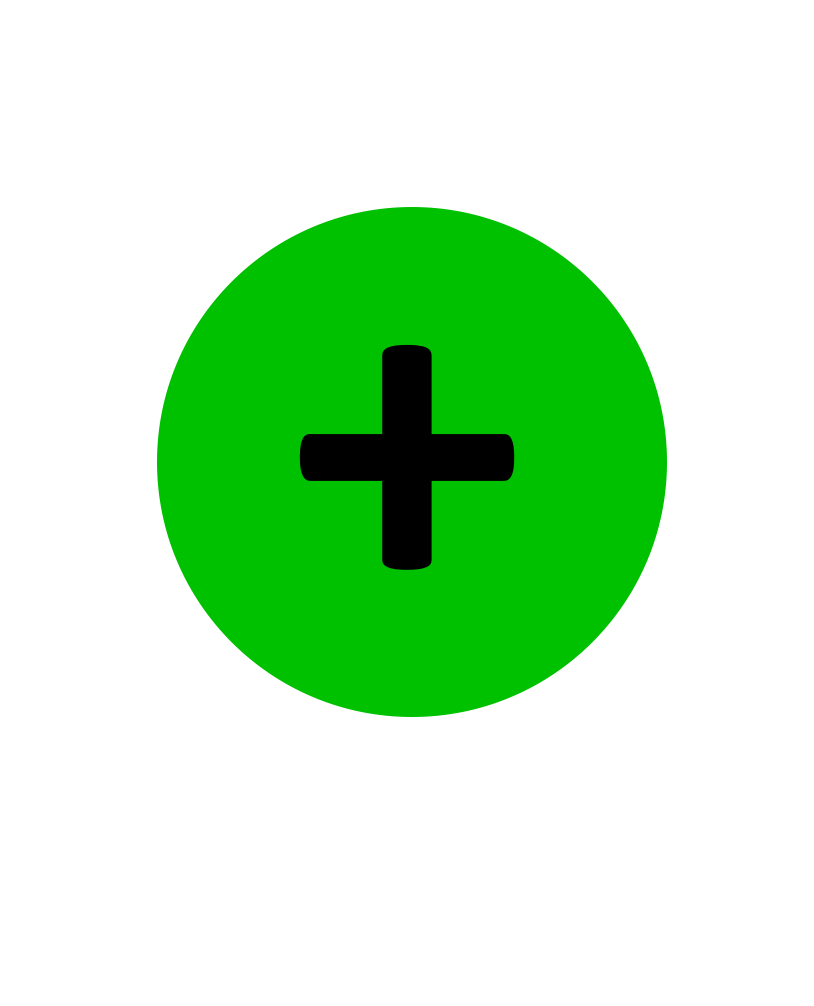 | 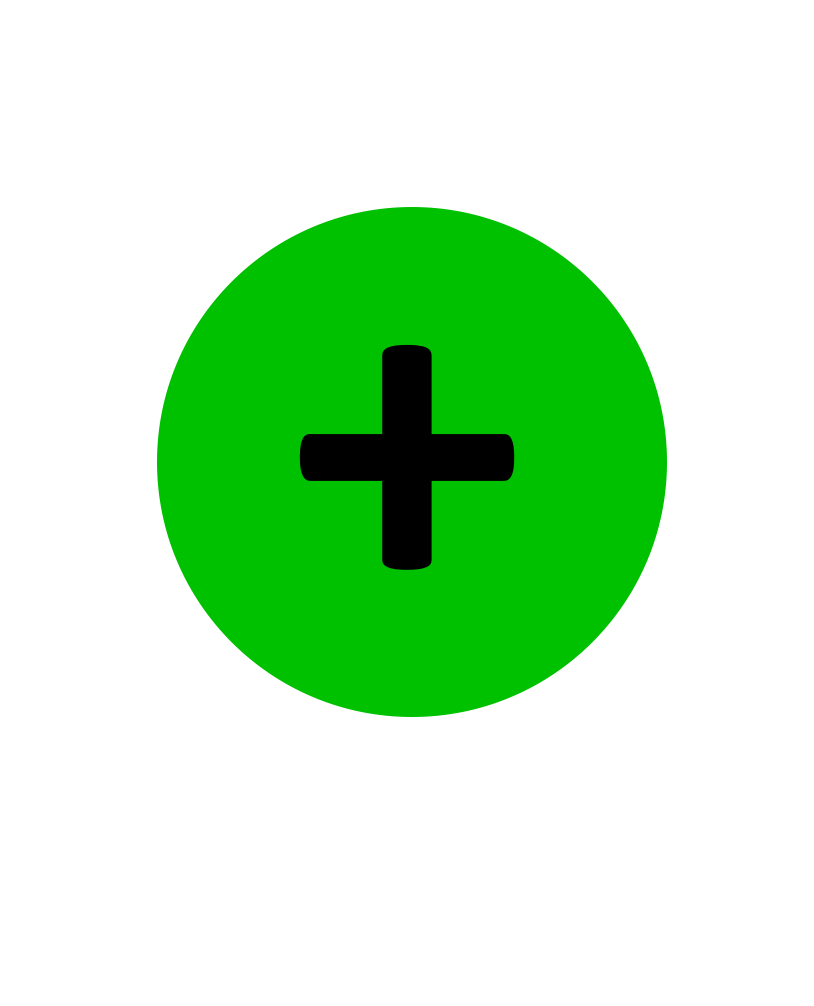 |
| **Fusar-Poli, 2017** [24] | | 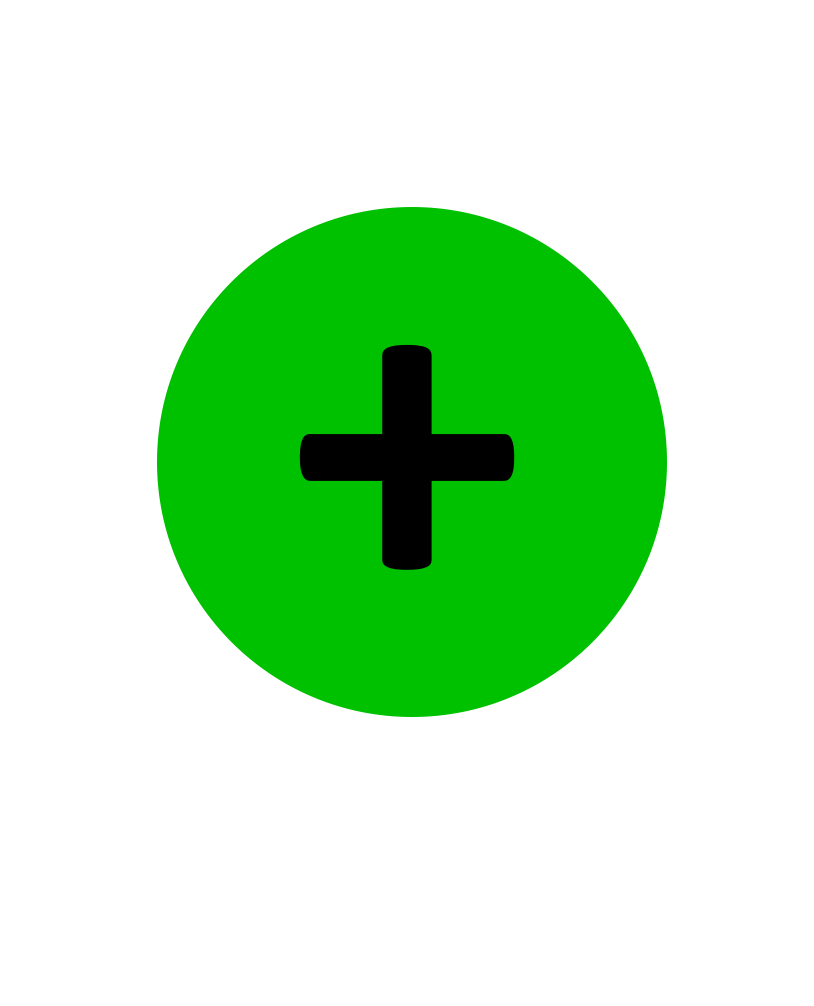 | 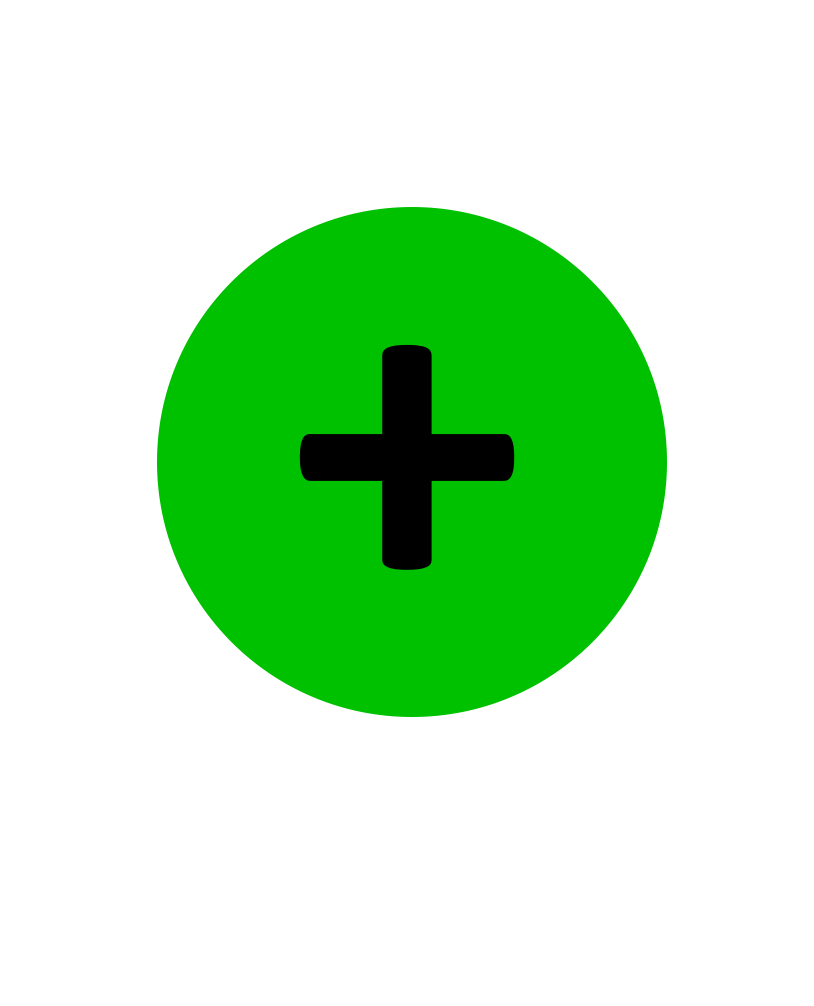 | 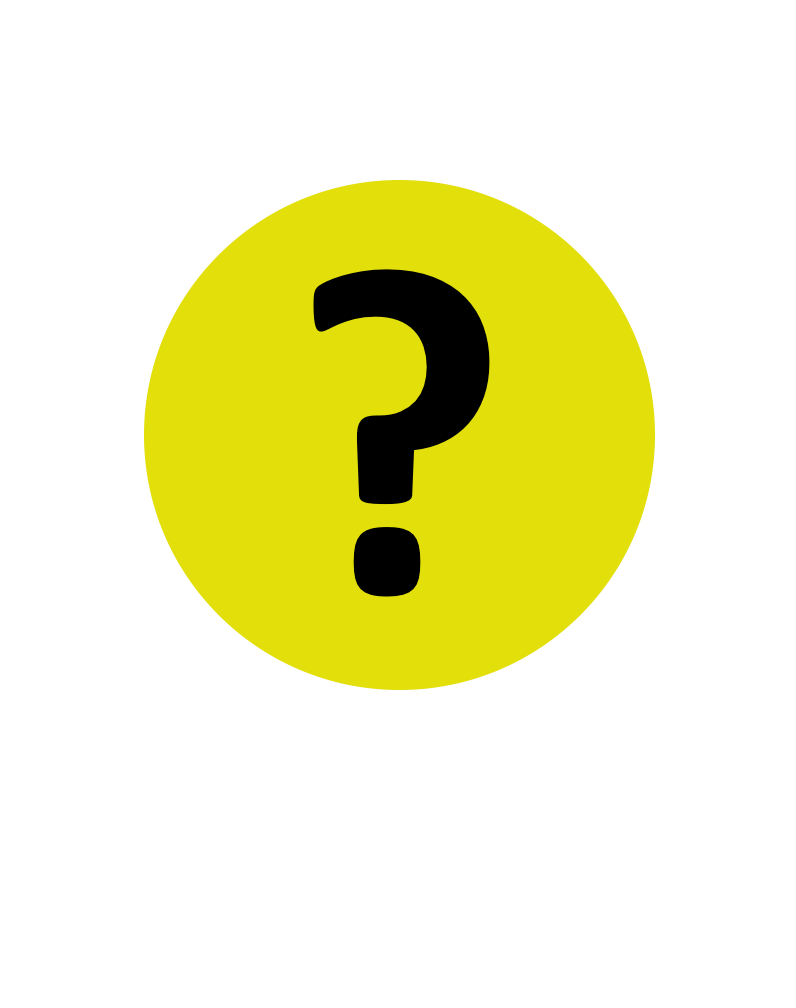 | 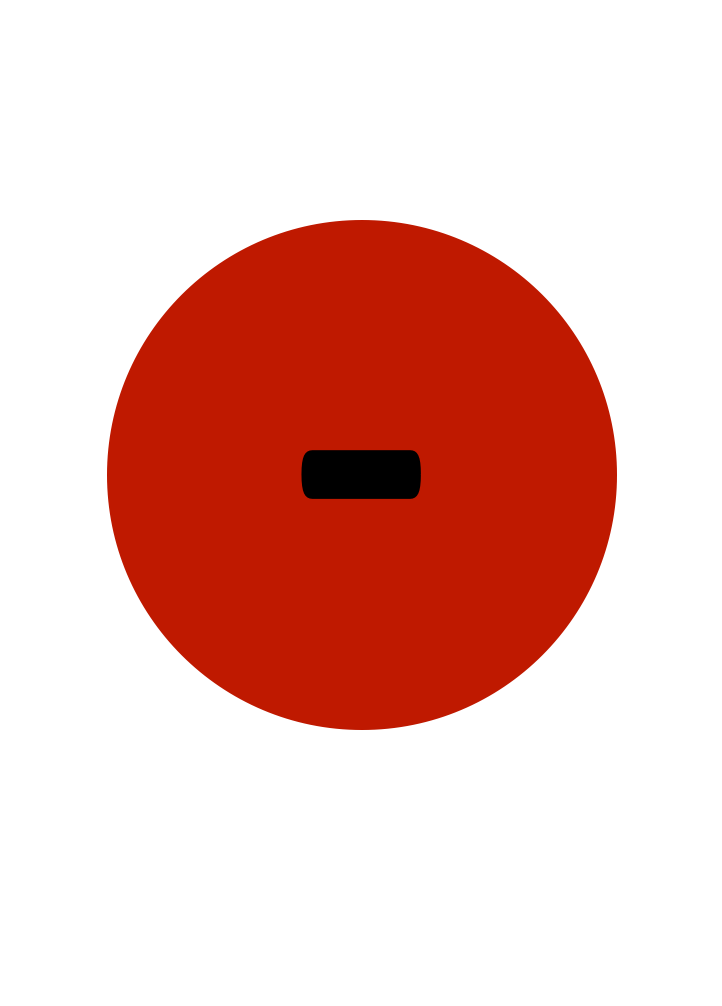 | 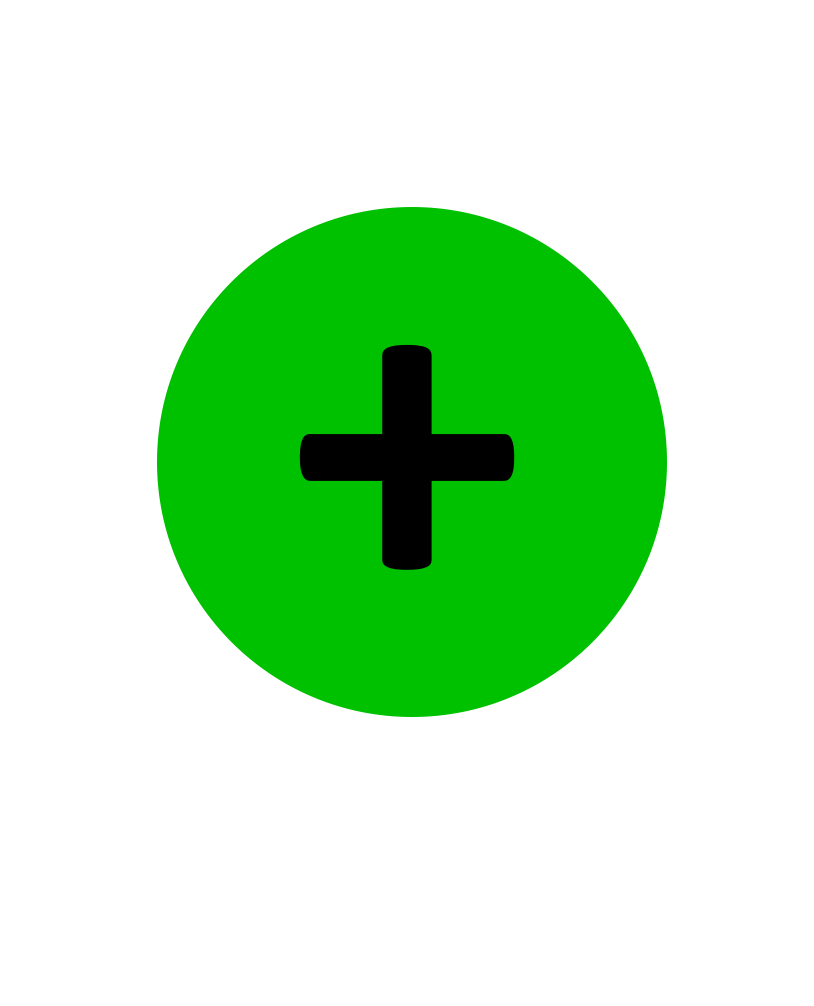 | 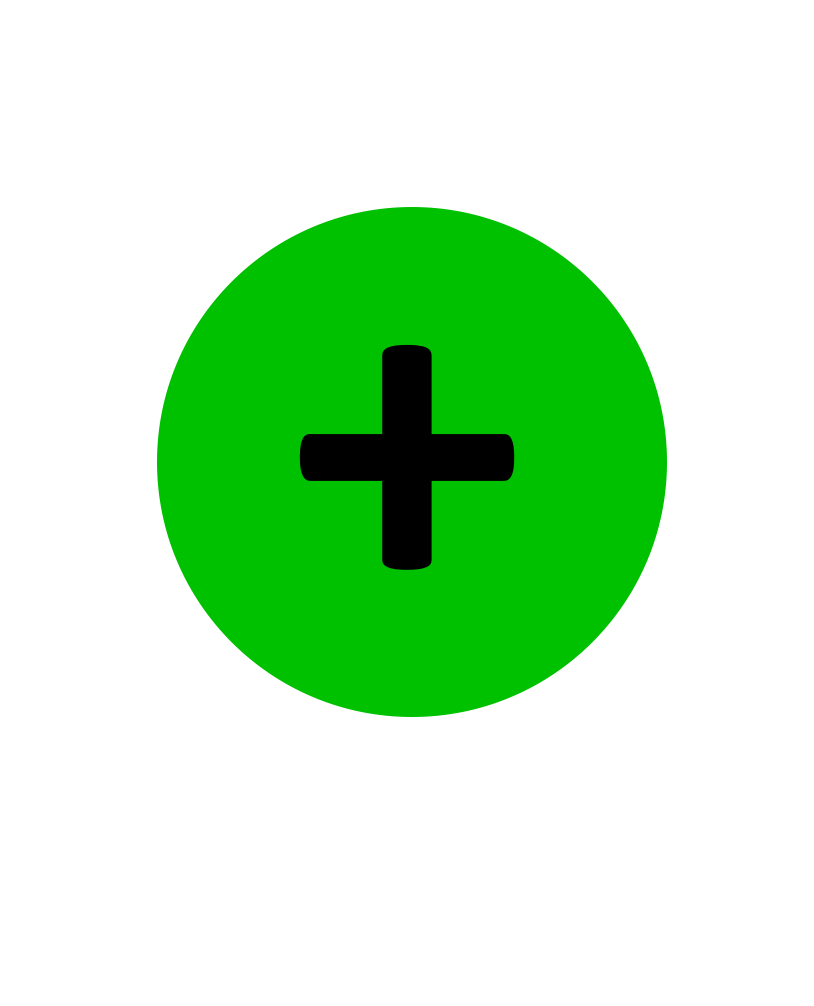 | 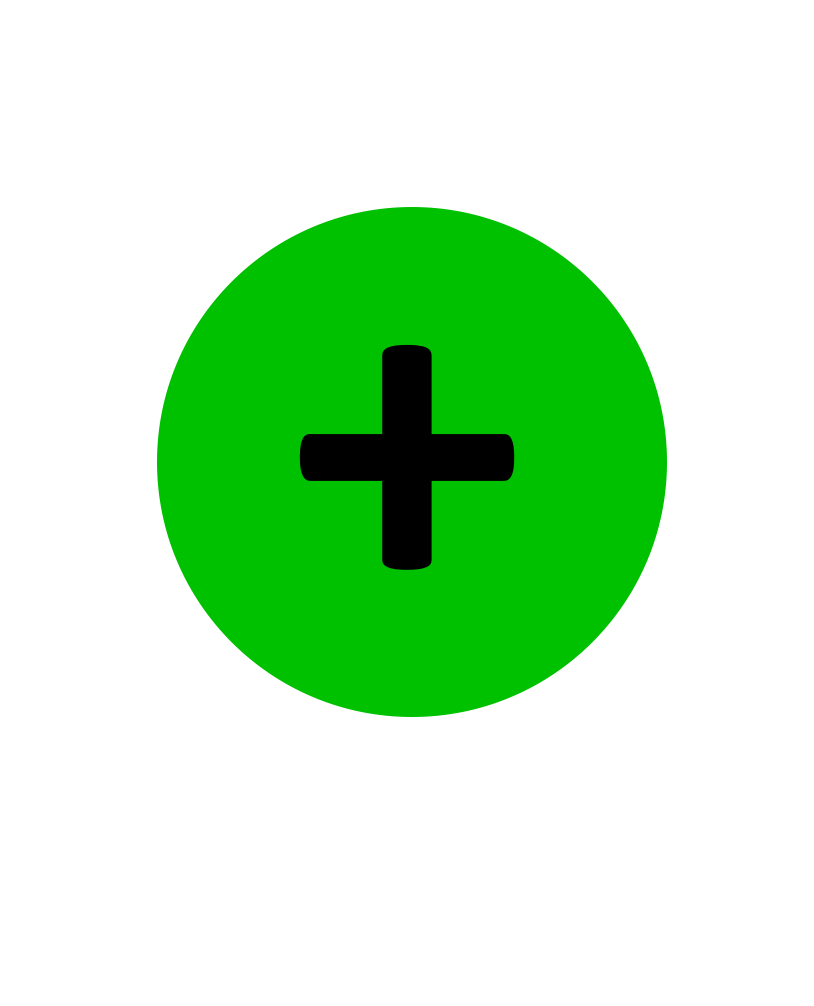 |
| **Masillo, 2018** [25] | | 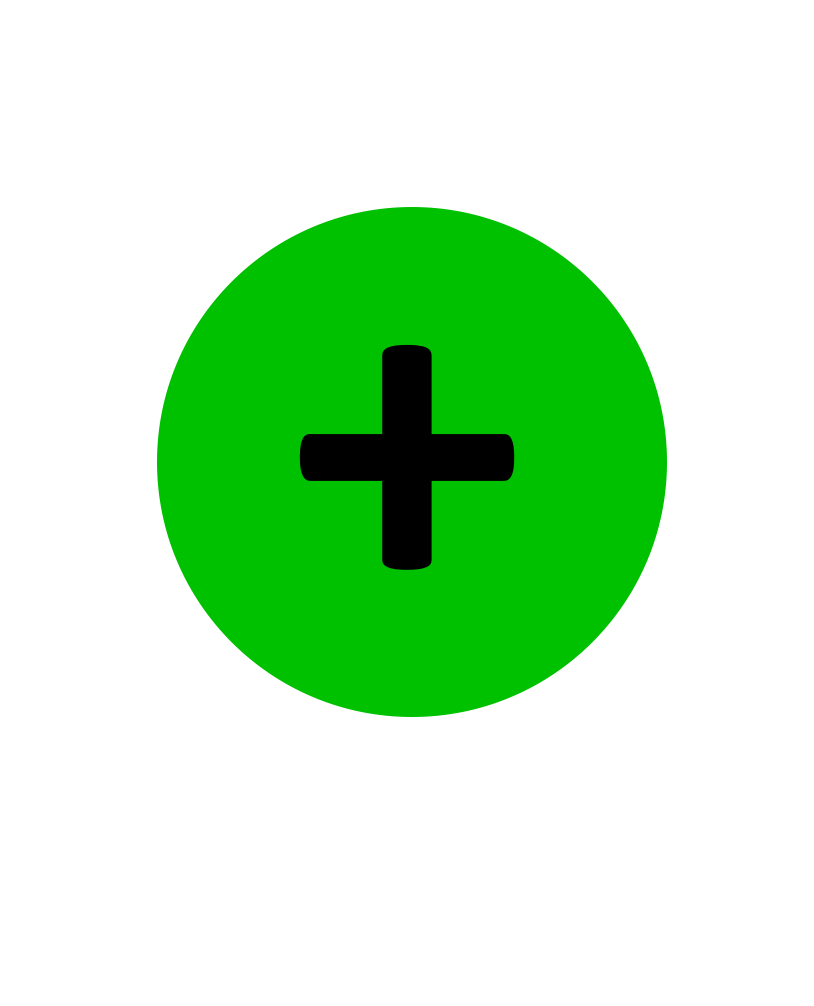 | 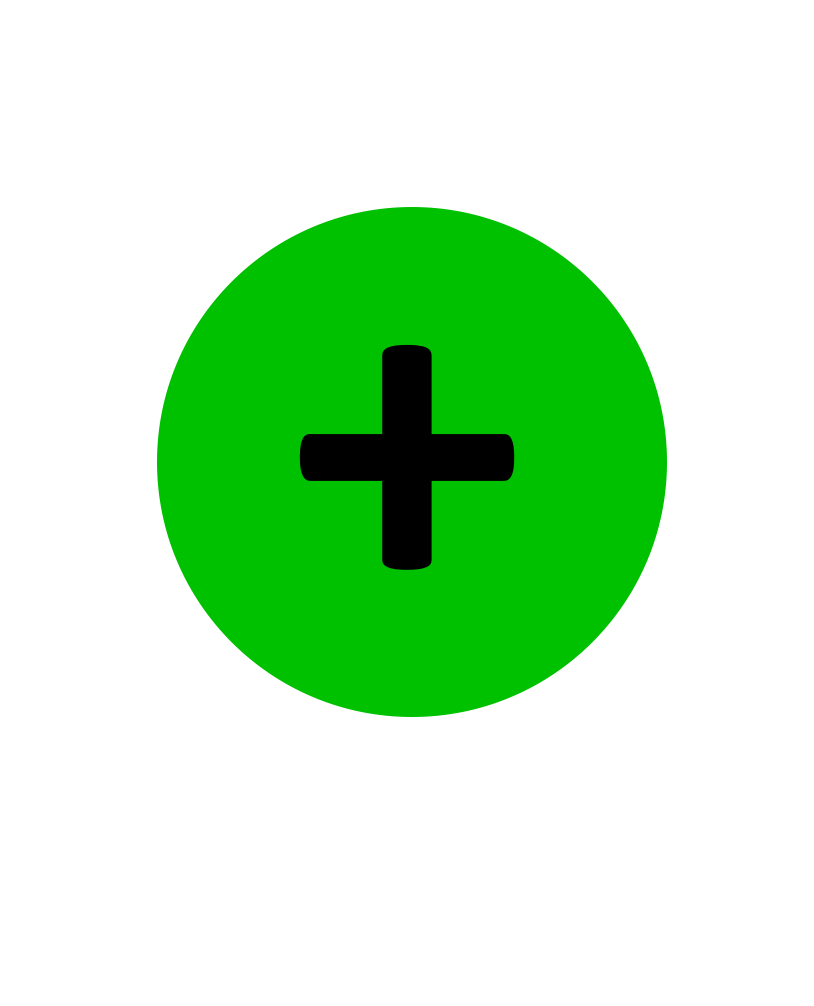 |  |  |  |  |  |
| **Papmeyer, 2018** [26] | |  |  |  |  |  |  |  |
| **Xu, 2018** [27] | |  |  |  |  |  |  |  |
| **Pelizza, 2019** [28] | |  |  |  |  |  |  |  |
| **Schneider, 2019** [29] | |  |  |  |  |  |  |  |
| **Mensi, 2021** [30] | |  |  |  |  |  |  |  |
| **NON-CLINICAL SAMPLES** | | | | | | | | |
| **Manninen, 2014** [31] | |  |  |  |  |  |  |  |
| **DIAGNOSTIC CRITERIA** | | | | | | | | |
| **Fusar-Poli, 2018** [32] | | **Low**  **Unclear**  **High** |  |  |  |  |  |  |
|  |  |  |  |  |  |  |  |  |

**eFigure 1** Summary of risk of bias and applicability concerns (QUADAS-2) in studies using CHR-P psychometric instruments on clinical samples.

Risk of bias and applicability concerns regarding the index test were unclear in one study [26] due to a potential lack of predefined threshold. Risk of bias relating to the reference standard was unclear in 17 studies [12, 14–19, 21–23, 25–30, 32] due to a lack of clarity about blinding of assessors at follow-up. Similarly, three studies [20, 24, 31] were considered to have a high risk of bias for the reference standard as medical records were used to inform the presence or absence of the outcome. 17 studies [11–13, 15–17, 19–21, 23, 25, 26, 28–32] were scored as having a high risk of bias relating to patient flow and timing, due to attrition over the follow-up period or a short follow-up period (less than two years). Patient flow and timing methods were considered unclear in a further four studies [14, 22, 24, 27], where not all patients may have been assessed with the same reference standard.

**eFigure 2** Forest plot showing study-specific and meta-analytic summary sensitivity (Se) and specificity (Sp) with corresponding heterogeneity statistics

**eFigure 3** Model diagnostics. One study [12] was detected as an outlier and reached the high influence threshold.

**eFigure 4** Meta-analytical summary receiver operating characteristic (SROC) curve of clinical high risk for psychosis (CHR-P) psychometric instruments in clinical samples at an average follow-up time of 34 months. Influential observations [12] as detected in the model diagnostics (eFigure 3 above) have been removed. Drop-outs are assumed to have equal risk of transitioning as individuals followed-up. N.B. x-axis for Sp runs reversed. Se – sensitivity, Sp – specificity, AUC – area under the curve, 1 – Klosterkötter et al [11], 2 – EXCLUDED Kobayashi et al [12], 3 – Yung et al [13], 4 – Woods et al [14], 5– Liu et al [15], 6 – Addington et al [16], 7 – Simon et al [17], 8 – Lee et al [18], 9 – Lindgren et al [19], 10 – Schultze-Lutter et al [20], 11 – Kline et al [21], 12 – Kotlicka-Antczak et al [22], 13 - Fusar-Poli et al [24], 14 – Francesconi et al [23], 15 – Pelizza et al, [28] 16 – Xu et al [27], 17 – Papmeyer et al [26], 18 - Masillo et al [25], 19 – Schneider et al [29], 20 - Mensi et al [30]

**eFigure 5** Deeks’ funnel plot for small-study effects/publication bias

**eResults 1** Sensitivity analyses assessing impact of follow-up time

The AUCs ranged from good to outstanding at each time point: at 6 months (k=9 [12–14, 16, 18, 20, 21, 26, 30], AUC=0.98, 95%CI: 0.96-0.99), at 12 months (k=10 [14, 16–20, 23, 26, 28, 30], AUC=0.82, 95%CI: 0.78-0.85), at 24 months (k=11 [14–18, 20, 26, 27, 30, 32], AUC=0.90, 95%CI: 0.87-0.92), and at ≥30 months (k=10 [11, 14, 16, 18–20, 22, 24, 29, 30], AUC=0.80, 95%CI: 0.76-0.83).

| **eTable 4** Sensitivity analyses estimating the prognostic accuracy of psychometric CHR-P instruments in clinical samples under different assumptions for individuals lost at follow-up (drop-outs) | | | | | | | | | |
| --- | --- | --- | --- | --- | --- | --- | --- | --- | --- |
| **Follow-up** | **Drop-out assumption** | **k** | **n** | **AUC** | **AUC 95% CI** | **Se** | **Se 95% CI** | **Sp** | **Sp 95% CI** |
| **Longest follow-up (mean 34 months)**[11–30] | Drop-outs excluded | 20 | 4,314 | 0.85 | 0.81-0.87 | 0.92 | 0.87-0.96 | 0.58 | 0.50-0.65 |
|  | Drop-outs no transition | 20 | 4,819 | 0.85 | 0.82-0.88 | 0.92 | 0.87-0.96 | 0.57 | 0.49-0.65 |
|  | Equal risk of transition in drop-outs and non-drop-outs | 20 | 4,819 | 0.85 | 0.81-0.88 | 0.93 | 0.87-0.96 | 0.58 | 0.50-0.66 |
|  | Drop-outs all transition | 20 | 4,819 | 0.70 | 0.66-0.74 | 0.81 | 0.67-0.90 | 0.58 | 0.50-0.65 |
| **6 months**[12–14, 16, 18, 20, 21, 26, 30] | Drop-outs excluded | 9 | 2,299 | 0.98 | 0.96-0.99 | 0.98 | 0.91-0.99 | 0.53 | 0.40-0.67 |
|  | Drop-outs no transition | 9 | 2,374 | 0.97 | 0.96-0.99 | 0.98 | 0.91-0.99 | 0.51 | 0.37-0.66 |
|  | Equal risk of transition in drop-outs and non-drop-outs | 9 | 2,374 | 0.98 | 0.96-0.99 | 0.98 | 0.91-0.99 | 0.54 | 0.40-0.68 |
|  | Drop-outs all transition | 9 | 2,374 | 0.71 | 0.66-0.74 | 0.86 | 0.61-0.96 | 0.54 | 0.40-0.67 |
| **12 months**[14, 16–20, 23, 26, 28, 30] | Drop-outs excluded | 10 | 2,293 | 0.84 | 0.81-0.87 | 0.94 | 0.87-0.97 | 0.50 | 0.39-0.60 |
|  | Drop-outs no transition | 10 | 2,489 | 0.83 | 0.79-0.86 | 0.94 | 0.87-0.97 | 0.47 | 0.36-0.58 |
|  | Equal risk of transition in drop-outs and non-drop-outs | 10 | 2,489 | 0.82 | 0.78-0.85 | 0.94 | 0.87-0.98 | 0.48 | 0.37-0.58 |
|  | Drop-outs all transition | 10 | 2,489 | 0.66 | 0.61-0.70 | 0.88 | 0.73-0.95 | 0.47 | 0.37-0.57 |
| **24 months**[13–18, 20, 26, 27, 30] | Drop-outs excluded | 11 | 2,703 | 0.86 | 0.83-0.89 | 0.94 | 0.88-0.97 | 0.51 | 0.41-0.62 |
|  | Drop-outs no transition | 11 | 2,927 | 0.94 | 0.91-0.95 | 0.95 | 0.91-0.97 | 0.50 | 0.38-0.62 |
|  | Equal risk of transition in drop-outs and non-drop-outs | 11 | 2,927 | 0.90 | 0.87-0.92 | 0.95 | 0.90-0.98 | 0.50 | 0.39-0.62 |
|  | Drop-outs all transition | 11 | 2,927 | 0.68 | 0.64-0.72 | 0.87 | 0.70-0.95 | 0.50 | 0.39-0.60 |
| ≥**30 months**[11, 14, 16, 18–20, 22, 24, 29, 30] | Drop-outs excluded | 10 | 3,122 | 0.80 | 0.76-0.83 | 0.90 | 0.82-0.95 | 0.53 | 0.42-0.64 |
|  | Drop-outs no transition | 10 | 3,180 | 0.79 | 0.76-0.83 | 0.90 | 0.82-0.95 | 0.53 | 0.42-0.63 |
|  | Equal risk of transition in drop-outs and non-drop-outs | 10 | 3,180 | 0.80 | 0.76-0.83 | 0.89 | 0.81-0.94 | 0.53 | 0.42-0.64 |
|  | Drop-outs all transition | 10 | 3,180 | 0.74 | 0.70-0.78 | 0.87 | 0.75-0.94 | 0.53 | 0.42-0.64 |
| Abbreviations: k, number of studies; n, sample size; AUC, area under the curve; Se, sensitivity; Sp, specificity | | | | | | | | | |

**eFigure 6** Sensitivity analyses estimating the prognostic accuracy of psychometric CHR-P instruments in clinical samples under different assumptions for individuals lost at follow-up (drop-outs)

**eTable 5** Sensitivity analyses assessing impact of single studies on meta-analytical summary estimates through leave-one-out analysis

| **Study removed** | **AUC** | **AUC**  **95%CI** | **Se** | **Se**  **95%CI** | **Sp** | **Sp**  **95%CI** |
| --- | --- | --- | --- | --- | --- | --- |
| **Klosterkötter, 2001** [11] | 0.85 | 0.82-0.88 | 0.91 | 0.86-0.95 | 0.57 | 0.49-0.65 |
| **Kobayashi, 2008** [12] | 0.83 | 0.80-0.86 | 0.92 | 0.87-0.96 | 0.55 | 0.48-0.63 |
| **Yung, 2008** [13] | 0.85 | 0.81-0.88 | 0.93 | 0.87-0.96 | 0.57 | 0.48-0.65 |
| **Woods, 2009** [14] | 0.85 | 0.82-0.88 | 0.92 | 0.86-0.95 | 0.58 | 0.50-0.66 |
| **Liu, 2011** [15] | 0.85 | 0.82-0.88 | 0.92 | 0.86-0.95 | 0.57 | 0.49-0.66 |
| **Addington, 2012** [16] | 0.85 | 0.82-0.88 | 0.93 | 0.87-0.96 | 0.58 | 0.50-0.66 |
| **Simon, 2012** [17] | 0.85 | 0.82-0.88 | 0.92 | 0.86-0.95 | 0.59 | 0.50-0.66 |
| **Lee, 2013** [18] | 0.84 | 0.80-0.87 | 0.92 | 0.86-0.96 | 0.56 | 0.48-0.64 |
| **Lindgren, 2014** [19] | 0.87 | 0.84-0.89 | 0.93 | 0.88-0.96 | 0.57 | 0.48-0.65 |
| **Schultze-Lutter, 2014** [20] | 0.84 | 0.81-0.87 | 0.92 | 0.86-0.96 | 0.59 | 0.51-0.66 |
| **Kline, 2015** [21] | 0.85 | 0.82-0.88 | 0.92 | 0.87-0.95 | 0.57 | 0.48-0.65 |
| **Kotlicka-Antczak, 2015** [22] | 0.85 | 0.82-0.88 | 0.93 | 0.87-0.96 | 0.58 | 0.49-0.66 |
| **Francesconi, 2017** [23] | 0.86 | 0.82-0.89 | 0.93 | 0.87-0.96 | 0.57 | 0.49-0.66 |
| **Fusar-Poli, 2017** [24] | 0.87 | 0.84-0.90 | 0.93 | 0.88-0.96 | 0.58 | 0.50-0.66 |
| **Masillo, 2018** [25] | 0.85 | 0.81-0.88 | 0.92 | 0.87-0.95 | 0.57 | 0.48-0.65 |
| **Papmeyer, 2018** [26] | 0.85 | 0.82-0.88 | 0.92 | 0.86-0.95 | 0.59 | 0.51-0.66 |
| **Xu, 2018** [27] | 0.84 | 0.80-0.87 | 0.93 | 0.87-0.96 | 0.56 | 0.48-0.64 |
| **Pelizza, 2019** [28] | 0.85 | 0.82-0.88 | 0.92 | 0.87-0.95 | 0.57 | 0.49-0.66 |
| **Schneider, 2019** [29] | 0.85 | 0.82-0.88 | 0.93 | 0.88-0.96 | 0.57 | 0.49-0.65 |
| **Mensi, 2021** [30] | 0.85 | 0.82-0.88 | 0.92 | 0.87-0.96 | 0.57 | 0.49-0.66 |
| Abbreviations: AUC, area under the curve; CI, confidence interval; Se, sensitivity; Sp, specificity | | | | | | |

**eFigure 7** Meta-regression results for age, gender, follow-up time, sample size and pre-screening

**eFigure 8** Meta-regression of CHR-P instrument on prognostic accuracy in clinical samples employing CAARMS and SIPS

**eDiscussion** Prognostic accuracy in DSM-5-APS samples

Only two eligible studies reported data on using diagnostic criteria (i.e. DSM-5-APS). One study had full agreement between the DSM-5-APS and the CAARMS samples [30], suggesting similar, if not equivalent prognostic accuracy and clinical utility in DSM-5-APS samples. This study [30] reported similar performance metrics to our summary metrics (Se = 0.96, 95%CI: 0.78-1.00; Sp = 0.53, 95%CI: 0.46-0.61).

However, the other had a lower proportion of individuals at risk according to the DSM-5-APS compared to the CAARMS [24, 32]. This is most likely due to a higher number of individuals meeting BLIPS and GRD (CAARMS) criteria, who would not meet DSM-5-APS criteria. This study [32] reported Se (0.65, 95%CI: 0.38-0.86) that appeared to be lower in the DSM-5-APS sample compared to the CAARMS sample (Se=0.78, 95%CI: 0.65-0.88). However, Sp (0.68, 95%CI: 0.59-0.76) was substantially higher in DSM-5-APS sample compared to the CAARMS sample (Sp=0.44, 95%CI: 0.41-0.48) with 95%CIs not overlapping.

**eReferences**

1. Salazar de Pablo G, Catalan A, Fusar-Poli P. Clinical Validity of DSM-5 Attenuated Psychosis Syndrome: Advances in Diagnosis, Prognosis, and Treatment. JAMA Psychiatry. 2020;77:311–320.

2. Fusar-Poli P. The Clinical High-Risk State for Psychosis (CHR-P), Version II. Schizophrenia Bulletin. 2017;43:44–47.

3. Fusar-Poli P, Salazar de Pablo G, Correll CU, Meyer-Lindenberg A, Millan MJ, Borgwardt S, et al. Prevention of psychosis: advances in detection, prognosis, and intervention. JAMA Psychiatry. 2020;77:755–765.

4. Fusar-Poli P, Cappucciati M, Rutigliano G, Schultze-Lutter F, Bonoldi I, Borgwardt S, et al. At risk or not at risk? A meta-analysis of the prognostic accuracy of psychometric interviews for psychosis prediction. World Psychiatry : Official Journal of the World Psychiatric Association (WPA). 2015;14:322–332.

5. Higgins JPT, Thompson SG, Deeks JJ, Altman DG. Measuring inconsistency in meta-analyses. BMJ (Clinical Research Ed). 2003;327:557–560.

6. Dwamena B. MIDAS: computational and graphical routines for meta-analytical integration of diagnostic accuracy studies in Stata. Ann Arbor: Division of Nuclear Medicine, Department of Radiology, University of Michigan Medical School,; 2007.

7. Li J, Fine JP, Safdar N. Prevalence-dependent diagnostic accuracy measures. Statistics in Medicine. 2007;26:3258–3273.

8. Janda S, Shahidi N, Gin K, Swiston J. Diagnostic accuracy of echocardiography for pulmonary hypertension: a systematic review and meta-analysis. Heart. 2011;97:612–622.

9. Hosmer W, Lemeshow S. Applied Survival Analysis: Regression Modeling of Time to Event Data. New York, NY: Wiley & Sons; 1999.

10. Macaskill P, Gatsonis C, Deeks JJ, Harbord RM, Takwoingi Y. Chapter 10: Analysing and Presenting Results. Cochrane Handbook for Systematic Reviews of Diagnostic Test Accuracy Version 1.0., The Cochrane Collaboration.

11. Klosterkötter J, Hellmich M, Steinmeyer EM, Schultze-Lutter F. Diagnosing Schizophrenia in the Initial Prodromal Phase. Arch Gen Psychiatry. 2001;58:158.

12. Kobayashi H, Nemoto T, Koshikawa H, Osono Y, Yamazawa R, Murakami M, et al. A self-reported instrument for prodromal symptoms of psychosis: Testing the clinical validity of the PRIME Screen—Revised (PS-R) in a Japanese population. Schizophrenia Research. 2008;106:356–362.

13. Yung AR, Nelson B, Stanford C, Simmons MB, Cosgrave EM, Killackey E, et al. Validation of ‘prodromal’ criteria to detect individuals at ultra high risk of psychosis: 2 year follow-up. Schizophrenia Research. 2008;105:10–17.

14. Woods SW, Addington J, Cadenhead KS, Cannon TD, Cornblatt BA, Heinssen R, et al. Validity of the prodromal risk syndrome for first psychosis: findings from the North American Prodrome Longitudinal Study. Schizophrenia Bulletin. 2009;35:894–908.

15. Liu C-C, Lai M-C, Liu C-M, Chiu Y-N, Hsieh MH, Hwang T-J, et al. Follow-up of subjects with suspected pre-psychotic state in Taiwan. Schizophrenia Research. 2011;126:65–70.

16. Addington J, Piskulic D, Perkins D, Woods SW, Liu L, Penn DL. Affect recognition in people at clinical high risk of psychosis. Schizophrenia Research. 2012;140:87–92.

17. Simon AE, Grädel M, Cattapan-Ludewig K, Gruber K, Ballinari P, Roth B, et al. Cognitive functioning in at-risk mental states for psychosis and 2-year clinical outcome. Schizophrenia Research. 2012;142:108–115.

18. Lee J, Rekhi G, Mitter N, Bong YL, Kraus MS, Lam M, et al. The Longitudinal Youth at Risk Study (LYRIKS)–an Asian UHR perspective. Schizophrenia Research. 2013;151:279–283.

19. Lindgren M, Manninen M, Kalska H, Mustonen U, Laajasalo T, Moilanen K, et al. Predicting psychosis in a general adolescent psychiatric sample. Schizophr Res. 2014;158:1–6.

20. Schultze-Lutter F, Klosterkötter J, Ruhrmann S. Improving the clinical prediction of psychosis by combining ultra-high risk criteria and cognitive basic symptoms. Schizophrenia Research. 2014;154:100–106.

21. Kline E, Thompson E, Demro C, Bussell K, Reeves G, Schiffman J. Longitudinal validation of psychosis risk screening tools. Schizophrenia Research. 2015;165:116–122.

22. Kotlicka-Antczak M, Pawełczyk T, Rabe-Jabłońska J, Pawełczyk A. PORT (Programme of Recognition and Therapy): the first Polish recognition and treatment programme for patients with an at-risk mental state. Early Intervention in Psychiatry. 2015;9:339–342.

23. Francesconi M, Minichino A, Carrión RE, Delle Chiaie R, Bevilacqua A, Parisi M, et al. Psychosis prediction in secondary mental health services. A broad, comprehensive approach to the ‘at risk mental state’ syndrome. European Psychiatry. 2017;40:96–104.

24. Fusar-Poli P, Rutigliano G, Stahl D, Davies C, De Micheli A, Ramella-Cravaro V, et al. Long-term validity of the At Risk Mental State (ARMS) for predicting psychotic and non-psychotic mental disorders. European Psychiatry. 2017;42:49–54.

25. Masillo A, Brandizzi M, Valmaggia LR, Saba R, Lo Cascio N, Lindau JF, et al. Interpersonal sensitivity and persistent attenuated psychotic symptoms in adolescence. Eur Child Adolesc Psychiatry. 2018;27:309–318.

26. Papmeyer M, Aston J, Everts-Graber J, Heitz U, Studerus E, Borgwardt SJ, et al. Outcome of individuals “not at risk of psychosis” and prognostic accuracy of the Basel Screening Instrument for Psychosis (BSIP): PAPMEYER et al. Early Intervention in Psychiatry. 2018;12:907–914.

27. Xu L, Wang Y, Cui H, Tang Y, Wang J, Tang X, et al. Identification and prediction of clinical high risk of psychosis in Chinese outpatients using two-stage screening. Schizophrenia Research. 2018;202:284–290.

28. Pelizza L, Paterlini F, Azzali S, Garlassi S, Scazza I, Pupo S, et al. The approved Italian version of the comprehensive assessment of at-risk mental states (CAARMS-ITA): Field test and psychometric features. Early Intervention in Psychiatry. 2019;13:810–817.

29. Schneider M, Armando M, Schultze-Lutter F, Pontillo M, Vicari S, Debbané M, et al. Prevalence, course and psychosis-predictive value of negative symptoms in 22q11.2 deletion syndrome. Schizophrenia Research. 2019;206:386–393.

30. Mensi MM, Molteni S, Iorio M, Filosi E, Ballante E, Balottin U, et al. Prognostic Accuracy of DSM-5 Attenuated Psychosis Syndrome in Adolescents: Prospective Real-World 5-Year Cohort Study. Schizophrenia Bulletin. 2021:sbab041.

31. Manninen M, Lindgren M, Therman S, Huttunen M, Ebeling H, Moilanen I, et al. Clinical high-risk state does not predict later psychosis in a delinquent adolescent population. Early Interv Psychiatry. 2014;8:87–90.

32. Fusar-Poli P, De Micheli A, Cappucciati M, Rutigliano G, Davies C, Ramella-Cravaro V, et al. Diagnostic and Prognostic Significance of DSM-5 Attenuated Psychosis Syndrome in Services for Individuals at Ultra High Risk for Psychosis. Schizophrenia Bulletin. 2018;44:264–275.
